# Clinical effectiveness of procalcitonin‐ or C‐reactive protein‐guided antibiotic discontinuation protocols for adult patients who are critically ill with sepsis: a rapid systematic review and meta‐analysis

**DOI:** 10.1111/anae.70109

**Published:** 2026-01-08

**Authors:** Sonya Rafiq, Chunhu Shi, Shraboni Ghosal, Paul Dark, Tim Felton, Evangelos Kontopantelis, Jo Dumville

**Affiliations:** ^1^ Division of Nursing, Midwifery and Social Work, Faculty of Biology, Medicine and Health The University of Manchester Manchester UK; ^2^ Division of Informatics, Imaging and Data Sciences, Faculty of Biology, Medicine and Health The University of Manchester UK; ^3^ Division of Infection, Immunity and Respiratory Medicine, Faculty of Biology, Medicine and Health The University of Manchester UK; ^4^ Critical Care Unit Northern Care Alliance NHS Foundation Trust Manchester UK; ^5^ Intensive Care Unit Wythenshawe Hospital, Manchester University NHS Foundation Trust Manchester UK

**Keywords:** anti‐bacterial drugs, biomarkers, C‐reactive protein, procalcitonin, sepsis

## Abstract

**Introduction:**

Antibiotics are a first‐line treatment for sepsis, with guidelines recommending a 7‐ to 10‐day course. Prolonged antibiotic use carries significant risks, prompting growing interest in using inflammatory biomarkers, such as procalcitonin or C‐reactive protein, to guide clinical decision‐making on the duration of antibiotic therapy in patients who are critically ill. This rapid systematic review aims to assess the effectiveness and safety of using procalcitonin‐ or C‐reactive protein‐guided protocols for antibiotic discontinuation in patients who are critically ill with sepsis.

**Methods:**

We conducted systematic searches for articles published after January 2005 in relevant databases. Eligible studies included randomised controlled trials comparing procalcitonin‐ or C‐reactive protein‐guided protocols for antibiotic discontinuation with standard care, or with each other. Primary outcomes were duration of antibiotic therapy and mortality. Secondary outcomes included infection recurrences; secondary infections or superinfections; and the duration of intensive care and hospital stays.

**Results:**

We identified 21 eligible studies. Moderate certainty evidence from 19 trials, involving 6382 patients, indicated that procalcitonin‐guided protocols probably reduced antibiotic therapy by, on average, 2.0 days (95%CI ‐2.6 to ‐1.4) compared with standard care. Moderate certainty evidence from 18 trials, involving 6228 patients, suggested an average 5% reduction in mortality risk when using procalcitonin‐guided protocols compared with standard care (risk ratio 0.95, 95%CI 0.83–1.07). The evidence regarding C‐reactive protein‐guided protocols versus standard care approaches remained unclear, with very low to low certainty evidence available.

**Discussion:**

Combining relevant trials suggests that procalcitonin‐guided discontinuation protocols may be both safe and effective for patients who are critically ill with sepsis, with no increased risk in mortality. In contrast, the evidence supporting C‐reactive protein‐guided protocols is limited. These findings underscore the potential importance of utilising procalcitonin to inform antimicrobial stewardship practices, particularly in critical care settings.

## Plain Language Summary

**What we did:**

We looked at several research studies to see if using special blood tests called procalcitonin or C‐reactive protein could help health professionals decide when it is safe to stop giving antibiotics to very sick people with sepsis. We collected and summarised results from these studies and considered how well they were conducted.

**Why we did it:**

People with sepsis often need antibiotics but taking them for too long can cause problems. Health professionals want to know if these blood tests can help them decide to stop antibiotics earlier without putting patients at risk.

**What we found:**

Using procalcitonin blood tests helped healthcare professionals stop antibiotics about two days earlier, on average, compared to when the test was not used. Stopping antibiotics earlier did not increase patients' risk of dying. However, it is still not clear whether using procalcitonin tests affects people from getting sick again. There was not enough good research to know whether C‐reactive protein tests work well or are safe. Overall, using procalcitonin blood tests seems helpful and safe for guiding health professionals on when to stop antibiotics in very sick patients with sepsis.

## Introduction

Sepsis is a life‐threatening condition caused by a dysregulated immune response to infection, potentially resulting in tissue damage, organ dysfunction and death [1]. Sepsis is a significant global cause of morbidity and mortality, with an estimated 48.9 million cases and 11 million sepsis‐related deaths in 2017, accounting for approximately 19.7% of all global deaths [2].

Antibiotic therapy is a first‐line treatment for sepsis, with international guidelines recommending a 7‐ to 10‐day course [3], adjusted based on clinical severity and daily assessment of treatment responses [4]. However, prolonged or inappropriate antibiotic use carries significant risks [5, 6, 7]. There is growing interest in using inflammatory biomarkers, such as procalcitonin (PCT) or C‐reactive protein (CRP), to guide clinical decision‐making on the duration of antibiotic therapy in people with sepsis.

Procalcitonin and CRP are circulating protein biomarkers of systemic inflammation produced by various cells in the body [8]. In healthy individuals, their levels are low but rise significantly in response to inflammatory stimuli (including systemic infection). Measured in blood samples using laboratory assays, these inflammatory biomarkers can help monitor disease progression and guide clinical decision‐making. For example, in the setting of bacterial infections and sepsis, elevated levels suggest ongoing infection, while marked decreases indicate treatment resolution, aiding safe antibiotic discontinuation in patients who are critically ill with sepsis [8]. Previous systematic reviews and meta‐analyses found that biomarker‐guided discontinuation protocols, compared with standard care approaches, may reduce the duration of antibiotic therapy without adversely affecting clinical outcomes in patients who are critically ill with sepsis [9, 10, 11]. However, evidence synthesis focused on antibiotic discontinuation is limited due to heterogeneity with marked variation in biomarker‐based protocols, biomarker algorithms and measurement timings [12]. Currently, due to insufficient evidence, the National Institute for Health and Care Excellence has not recommended the routine use of PCT or CRP in clinical settings to guide decision‐making on antibiotic discontinuation in patients with sepsis [13].

Recently, important randomised controlled trials have generated new evidence addressing biomarker‐guided antibiotic discontinuation [14, 15]. To date, these emerging data have not been synthesised comprehensively. This review aimed to evaluate the effectiveness and safety of PCT or CRP‐guided antibiotic discontinuation protocols in patients who are critically ill with sepsis compared with standard care practices. Specifically, this review addresses two key research questions. First, how effective and safe are PCT‐ and CRP‐guided protocols for discontinuing antibiotics compared with standard care in reducing: antibiotic usage; mortality rates; infection recurrence; new infections/superinfections; and duration of ICU stay and hospitalisation in adults critically ill with sepsis? Second, what are the optimal biomarker‐guided protocols for the discontinuation of antibiotic therapy in adult patients who are critically ill with sepsis?

By integrating the most recent randomised controlled trial data, this review aimed to inform clinical decision‐making and evidence‐based antibiotic stewardship strategies, thereby supporting more personalised, safe and resource‐efficient care for patients with sepsis [16, 17].

## Methods

This systematic review followed the rapid review guidance from the Cochrane Rapid Reviews Methods Group [18], given the time and resource constraints. The review is reported following PRISMA 2020 guidelines [19].

We implemented a sensitive search strategy in the MEDLINE, Embase Cumulative Index to Nursing and Allied Health Literature (CINAHL) and the Cochrane Central Register of Controlled Trials (CENTRAL) databases (see online Supporting Information Figure S1 for Medline search terms). We searched from January 2005 to April 2025 to identify articles published over the past two decades, following a rapid review methodology to ensure both accuracy and workload management [20]. There were no restrictions by language or publication status. In addition, we searched ClinicalTrials.gov and the World Health Organization International Clinical Trials Registry Platform to identify ongoing trials. Finally, we also reviewed the reference lists of pertinent recent systematic reviews [9] for potentially relevant studies and utilised the automation tool ‘citationchaser’ [21] to conduct backwards and forward citation searches, identifying additional relevant references.

Two reviewers (SR and SG or CS) screened all titles and abstracts independently to identify potentially eligible studies. These reviewers then screened the full‐text articles of these studies independently using Rayyan (Rayyan Systems, Cambridge, MA, USA). At each stage, the reviewers resolved any conflicts through discussion and consulted a third reviewer (JD, PD or TF) to arbitrate any unresolved queries and discrepancies when necessary.

Trials were eligible if they included patients aged ≥ 16 years old with confirmed or highly suspected sepsis, requiring antibiotic and organ support therapy and receiving treatment in critical or intensive care settings. As the definition of sepsis can vary (both over time and geography), where the criteria of a study were ambiguous, we used a sequential organ failure assessment (SOFA) score of ≥2 [1] and consulted with clinicians (PD and TF) to determine eligibility. Included trials evaluated protocols for PCT‐ or CRP‐guided antibiotic discontinuation, including those approaches that integrate decision or advice support tools with biomarker‐guided protocols. Studies were also required to use standard approaches to antibiotic discontinuation (without the use of PCT‐ or CRP‐guided protocols) as a comparator. We also included studies that compared PCT‐guided protocols directly with CRP‐guided protocols. Only randomised controlled trials were eligible, including parallel, crossover and cluster trials.

We did not include studies that: comprised mixed patients with and without infection; had no patients in critical care or intensive care settings; involved neonatal, paediatric or adolescent patients; and that used PCT‐ or CRP‐guided protocols to initiate antibiotic treatments or to guide a broader antibiotic management plan which included both antibiotic initiation and discontinuation decision‐making.

The primary effectiveness outcome was the duration of antibiotic therapy, and the primary safety outcome was mortality, defined as death from any cause. We considered mortality in the short (≤ 30 days), medium (1–2 months) and long term (> 2 months) post‐randomisation. Secondary outcomes were infection recurrences; secondary infections or superinfections; and duration of hospital and ICU stays. Infection recurrence was defined as any instance of relapse or reinfection requiring further antibiotic treatment. Secondary infections or superinfections were defined as new infections occurring at different anatomical sites.

Following the Cochrane rapid review guidelines [18], a single reviewer (SR, CS or SG) conducted data extraction using a predefined, piloted data extraction form. A second reviewer subsequently verified the accuracy and completeness of the extracted data. When necessary, we contacted study authors to obtain missing data that was not reported in the published articles.

We used the Cochrane Collaboration's Risk of Bias tool, version 2 (RoB2) to assess the risk of bias in included studies [22]. We followed a streamlined approach consistent with rapid review guidelines [18]. For each of the outcomes, the risk of bias was assessed across the following domains: randomisation; deviations from the intended intervention; missing outcome data; outcome measurement; and selection of reported results. Each study was categorised as having a low risk of bias, some risk of bias or a high risk of bias for both primary and secondary outcomes, and traffic light plots of domain‐level judgements were visualised using the ‘robvis’ package [23].

We acknowledge the challenges clinical staff face in remaining blinded to treatment allocation in randomised controlled trials focused on decision‐making strategies, which risks performance bias from unintended interventions or differential co‐interventions affecting outcome measures [24]. Thus, when assessing this risk of bias domain, we examined the blinding strategies used in included studies to mitigate performance bias when making our judgements.

We summarised the characteristics of the included studies, using descriptive statistics, in terms of study designs, patients and the PCT‐ or CRP‐guided protocols and standard care approaches used. We assessed clinical and methodological heterogeneity for these study characteristics and overall study‐level risk of bias [25]. We pooled study data if these characteristics were sufficiently similar and synthesised evidence narratively when these characteristics were substantially heterogeneous.

All statistical analyses were conducted in R version 4.5.1 (R Foundation, Vienna, Austria) using the ‘meta’ [26] and ‘metafor’ [27] packages. The ‘dmetar’ package [28] was used to support meta‐analysis procedures and conduct additional outlier and influence analyses (e.g. Baujat plots [29] and leave‐one‐out analyses). For binary outcomes, pooled effect sizes are reported as risk ratios (RRs) with 95%CIs. A random‐effects model was applied using the Mantel–Haenszel method. Between‐study variance (τ^2^) was estimated using the Paule‐Mandel estimator to account for small event rates, as recommended by Harrer et al. [28]. While the Mantel–Haenszel method was developed originally as a fixed‐effects model, in this analysis it was used within a random‐effects framework, with pooled estimates calculated using inverse variance weighting. This approach aligns with the methodology used in Review Manager 5 (Cochrane Collaboration, London, UK) and adheres to recommendations for random‐effects meta‐analysis using Mantel–Haenszel pooling [25, 28]. For continuous outcomes, such as duration of antibiotic therapy measured in days, the mean difference was used as the effect size, as the scale was consistent across studies. When medians were reported instead of means and standard deviations, these were converted using the ‘meta‐analysis accelerator’ software [30], which uses the formula by Wan et al. [31]. Continuous outcomes were pooled using inverse variance weighting, with between‐study heterogeneity estimated via the restricted maximum likelihood method [27], as suggested by Harrer et al. [28]. The Knapp‐Hartung adjustment [32] was applied to estimate the confidence intervals around the pooled effects. Forest plots were generated to present all primary outcome data visually.

Statistical heterogeneity was quantitatively assessed using the χ^2^ test and the Higgins and Thompson I^2^ statistics; a value of ≥ 75% suggests high heterogeneity [33]. For analyses involving ≥ 10 studies, prediction intervals were calculated to estimate the expected range of effects in future studies [25]. Publication bias was evaluated through visual inspection of funnel plots and Egger's regression test, with statistical significance set at p < 0.050, when > 10 studies were available [25].

To explore heterogeneity in the analyses of the primary outcomes, where ≥10 studies were available [25], we planned to conduct six predefined subgroup analyses, including univariable meta‐regression analyses where appropriate: patient sepsis severity (using baseline SOFA scores as a proxy measure of illness severity); timing of biomarker measurements, such as daily or non‐daily measurements; setting of sepsis acquisition, such as community‐ or hospital‐acquired; site of the sepsis infection, such as respiratory or abdominal sepsis; level of reported adherence to discontinuation protocols; and patient comorbidities (using baseline APACHE scores as a proxy measure of comorbidities). The choice of these study characteristics was driven by existing literature on study characteristics that may influence the relative effectiveness of biomarker‐guided decision‐making on antibiotic use in this population [9]. However, the availability of data only allowed us to perform subgroup analysis for patient sepsis severity; timings; and patient comorbidities. We also performed two post hoc subgroup analyses stratified by PCT absolute and relative threshold levels, as well as by study‐level risk of bias (low/some vs. high). Sensitivity analyses assessed the sensitivity of primary findings to the exclusion of studies that used converted means or varied outcome measure definitions (e.g. ICU, in‐hospital or all‐cause mortality).

A single reviewer (SR) conducted the GRADE assessments to evaluate the certainty of evidence for each outcome, rating it as high, moderate, low or very low certainty [34]. A second reviewer (CS) verified all assessments, with the involvement of a third reviewer (JD) as required (see online Supporting Information Appendix S1 for extended methods). The results of the GRADE assessment for each outcome are presented in the ‘Summary of Findings’ tables generated by GRADEpro (Evidence Prime, Hamilton, ON, Canada).

## Results

**Figure 1:**
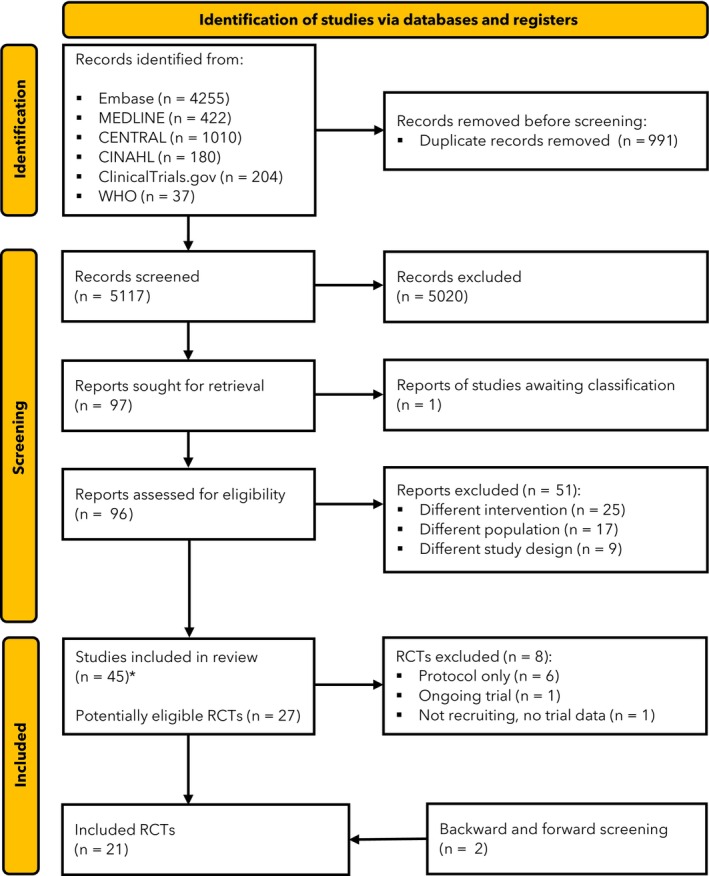
Study flow diagram. *Reports included in the review which included protocols and conference proceedings of eligible randomised controlled trials (RCTs).

The database search identified a total of 6108 records. From these, 29 randomised controlled trials were eligible, represented by 45 reports. Of the included randomised controlled trials, one had not yet started recruiting [35], one was ongoing with only a trial protocol published [36] and for six others, we only had limited information from trial registry records and no links to full publications [37, 38, 39, 40, 41, 42]; therefore, we did not consider them further. As a result, data from a maximum of 21 studies were included in our meta‐analyses (Fig. 1).

Of these 21 studies, 18 compared PCT‐guided antibiotic discontinuation protocols to standard care only [15, 43, 44, 45, 46, 47, 48, 49, 50, 51, 52, 53, 54, 55, 56, 57, 58, 59]. One trial had three groups, comparing both PCT‐ and CRP‐guided protocols to standard care [14]. Additionally, one trial compared a CRP‐guided protocol with standard care [60], and another compared a PCT‐ with a CRP‐guided protocol for discontinuation of antibiotics [61].

Baseline characteristics of each eligible study are reported in Table 1 and online Supporting Information Tables S1 and S2. Most included studies were parallel randomised controlled trials conducted between 2008 and 2025. In studies comparing PCT‐guided antibiotic discontinuation with standard care, the median of the average age was 65 y, and the median of the reported averages for the baseline SOFA and APACHE scores was 6.66 and 20.55, respectively. Sepsis definitions varied, with most studies using Sepsis‐1 [43, 44, 45, 47, 50]; Sepsis‐2 [49, 51]; or Sepsis‐3 criteria [14, 56, 57, 58, 60]. Five studies used unspecified criteria [15, 48, 53, 54, 59]; one was unclear [61]; and three met the eligibility criteria for highly suspected sepsis [46, 52, 55].

**Table 1:** Summary of the baseline characteristics in the included studies. Values are number (proportion).

	PCT vs. standard care	CRP vs. standard care	PCT vs. CRP
	(n = 19)	(n = 2)	(n = 2)
Total sample size	6415	1972	1936
Sex; female	2586 (41%)	799 (41%)	1150 (60%)
Severity			
Septic shock	2560 (46%)	944 (48%)	958 (50%)
Sepsis	2958 (54%)	1012 (52%)	969 (50%)
Infection site			
Lungs	3000 (47%)	956 (40%)	941 (40%)
Abdomen	1312 (21%)	435 (18%)	440 (18%)
Urinary tract	617 (10%)	247 (10%)	242 (10%)
Skin and soft tissue	290 (5%)	166 (7%)	142 (6%)
Blood stream	251 (4%)	174 (7%)	174 (7%)
Catheter‐related	51 (1%)	30 (1%)	28 (1%)
Central nervous system	110 (2%)	54 (2%)	63 (3%)
Eye, nose and throat	62 (1%)	48 (2%)	38 (2%)
Thoracic or surgical wound	85 (2%)	‐	‐
Unknown or other	616 (10%)	297 (12%)	312 (13%)
Place of infection			
Community associated	2267 (61%)	1234 (68%)	1264 (69%)
Healthcare associated	1057 (29%)	577 (32%)	580 (31%)
ICU‐associated	389 (10%)	‐	‐

PCT, procalcitonin‐guided care; CRP, C‐reactive protein‐guided care.

In studies comparing PCT‐ or CRP‐guided care with standard care, antibiotic discontinuation in the control group was guided by various approaches: international, national or local guidelines for infection types or antibiotic stewardship [14, 43, 46, 47, 49, 50, 51, 55, 56, 57]; clinical judgement alone [54]; clinical judgement informed by routine laboratory blood test, patient clinical outcome, local guidelines and/or microbiological cultures [15, 44, 45, 52, 58, 60]; and unspecified methods [48, 53, 59]. Full details of both intervention and control protocols are in online Supporting Information Table S3.

The risk of bias assessment results for individual studies and their outcomes are shown in online Supporting Information Figures S2–S18. For duration of antibiotic therapy (79% of studies), duration of ICU stay (80%) and duration of hospital stay (77%), most studies were judged to have a high risk of bias (online Supporting Information Table S4). This was attributable primarily to concerns in detection bias, where blinding of outcome assessors (typically clinical staff delivering the intervention) was challenging, potentially affecting the measurement of subjective outcomes. Only one study was rated as having a low risk of bias across all domains and outcomes [14]. For infection recurrence, 83% of studies were rated high risk due to a lack of blinding and inadequate outcome ascertainment. Among the seven studies reporting secondary infections or superinfections, three were deemed high risk and three raised some concerns for similar reasons. For mortality, a more objective outcome, 78% of studies were rated as having some concerns due to inadequate reporting of allocation concealment and insufficient information to assess deviations from intended interventions.

**Figure 2:**
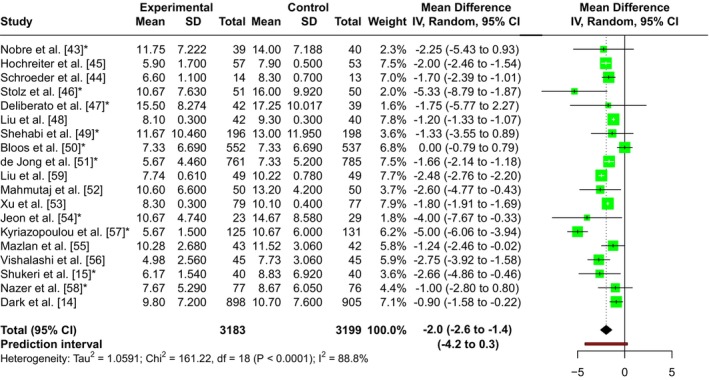
Duration of antibiotic therapy in patients receiving procalcitonin compared with standard care. *Studies where the median was converted to mean. Mean difference (days).

We included 19 randomised controlled trials (6382 patients) that reported duration of antibiotic therapy. There is moderate certainty evidence that the use of PCT‐guided discontinuation protocols may reduce duration of antibiotic therapy by, on average, 2.0 days compared with standard care (95%CI ‐2.6 to ‐1.4 days, p < 0.001, χ^2^ = 161.22, I^2^ = 88.8%; Table 2 and Fig. 2). No clear evidence of publication bias was found (online Supporting Information Figure S19 and Table S5).

**Table 2:** Summary of procalcitonin (PCT) compared with standard care for adult patients who were critically ill with sepsis. The risk in the intervention group (95%CI) is based on the assumed risk in the comparison group and the relative effect of the intervention (95%CI). Median durations of hospital stay, ICU stay, and antibiotic therapy are provided for the standard care group.

Outcomes	No. of patients (studies)	Certainty of evidence (GRADE)	Relative effect (95%CI)	Anticipated absolute effects	
				Risk with standard care	Risk difference with PCT
Duration of antibiotic therapy	6382 (19 RCTs)	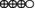 Moderate^a^	‐	10.2 days	MD 2.0 days lower (2.6 lower to 1.4 lower)
Short‐term mortality	6228 (18 RCTs)	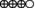 Moderate^e^	RR 0.95 (0.83–1.07)	224 per 1000	11 fewer per 1000 (38 fewer to 16 more)
Long‐term mortality	4974 (5 RCTs)	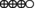 Moderate^e^	RR 0.95 (0.85–1.07)	454 per 1000	23 fewer per 1000 (68 fewer to 32 more)
Infection recurrence	4270 (11 RCTs)*	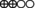 Low^a^, ^b^	RR 1.27 (0.96–1.68)	30 per 1000	8 more per 1000 (1 fewer to 20 more)
Secondary infection or superinfection	4083 (7 RCTs)	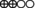 Low^b^, ^c^	RR 0.89 (0.53–1.49)	205 per 1000	23 fewer per 1000 (96 fewer to 100 more)
Duration of hospital stay	5699 (13 RCTs)	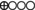 Very low^a^, ^b^, ^d^	‐	23.5 days	MD 1.0 days lower (3.8 lower to 1.9 higher)
Duration of ICU stay	5562 (15 RCTs)	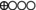 Very low^a^, ^b^, ^d^	‐	10.6 days	MD 1.2 days lower (3.7 lower to 1.3 higher)

MD, mean difference; RCT, randomised controlled trial; RR, risk ratio.

* For infection recurrence, 12 studies were identified, but the effect size data were generated based on data from 11 studies, as one study [54] included zero event data.

^a^ Downgraded by one level because most studies were rated as high risk of bias studies because of domain 4, but subgroup analysis found no difference between low and high‐risk‐of‐bias studies.

^b^ Downgraded by one level because of the wide confidence intervals.

^c^ Downgraded by one level because of moderate amounts of heterogeneity.

^d^ Downgraded by two levels because of a substantial amount of heterogeneity, and effect size estimates were in different directions.

^e^ Downgraded by one level as the confidence intervals cross the null threshold (null being a risk difference of 0%).

The effect estimates observed in the primary analysis were consistent across both sensitivity analyses, and we did not observe significant differences in effect estimates between risk of bias and biomarker measurement subgroups. For baseline SOFA scores, meta‐regression using data from 14 studies found that this moderator explained 70.2% of the between‐study variance. The regression coefficient indicated that for each one‐point increase in the SOFA score (suggesting more severe sepsis), the reduction in duration of antibiotic therapy decreased by 0.55 days (95%CI 0.18–0.92, p = 0.007). All sensitivity, subgroup and meta‐regression analyses are detailed in online Supporting Information Tables S6–S10.

It was unclear whether there is a difference between CRP‐guided antibiotic discontinuation protocols and standard care in terms of reducing duration of antibiotic therapy successfully (two randomised controlled trials (1927 patients) mean difference ‐0.6 days, 95%CI ‐11.0–9.9) and between PCT‐ and CRP‐guided antibiotic discontinuation protocols (two trials (1916 patients) mean difference ‐0.1 days, 95%CI ‐10.8–10.6). For both comparisons, the evidence was of very low certainty; see online Supporting Information Tables S11 and S12 for all results and Figures S20 and S21 for corresponding forest plots.

**Figure 3:**
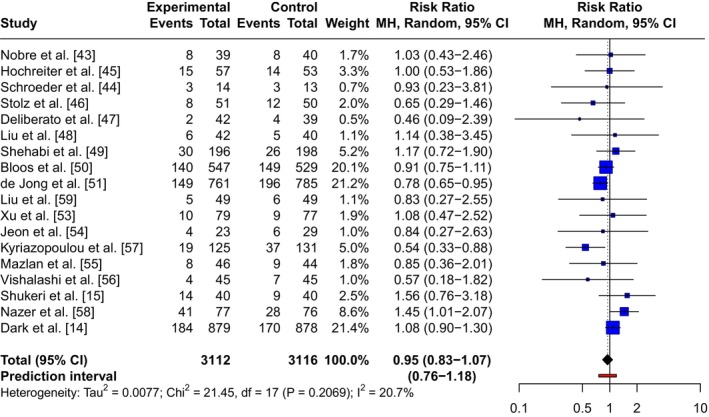
Mortality in the short‐term in patients receiving procalcitonin‐guided therapy compared with standard care. RR, risk ratio.

There were 18 included randomised controlled trials (6228 patients) that reported mortality over a short follow‐up period up to 30 days. Moderate certainty evidence suggested there was probably a 5% reduction in mortality risk, on average, when using PCT‐guided discontinuation protocols compared with standard care (pooled RR 0.95, 95%CI 0.83–1.07, χ^2^ = 21.45, I^2^ = 20.7%; Fig. 3). The confidence interval included the possibility of no effect and ranges from a 17% reduction to a 7% increase in mortality, in relative terms. Although the point estimate supports the intervention, due to imprecision, we cannot rule out the possibility of harm. Similar results were found for mortality measured over a longer follow‐up period of 90–365 days, based on 4974 patients, with a pooled RR of 0.95 (95%CI 0.85–1.07, χ^2^ = 7.57, I^2^ = 47.2%, moderate certainty evidence; online Supporting Information Figure S22). No clear evidence of publication bias was indicated (online Supporting Information Figure S23 and Table S5).

Sensitivity analyses conducted for 30‐day mortality reported consistent effect estimates for all three analyses. Subgroup analyses for 30‐day mortality showed no significant differences across the explored subgroups, with one exception: a significant difference was observed between subgroups stratified by PCT thresholds used for discontinuation decision‐making (p = 0.001). However, this finding should be interpreted cautiously due to the observational nature of the data and the small number of studies. In particular, one study may have influenced the subgroup result [58]; this study included patients with cancer and sepsis and reported a mortality risk of 45%, which was substantially higher than the average of 21% across the other included studies. When this study was removed from the analysis, the subgroup difference between PCT thresholds was no longer statistically significant (p = 0.090). Full details are in online Supporting Information Tables S8 and S9.

Two randomised controlled trials (1924 patients) compared CRP‐guided antibiotic discontinuation protocols with standard care. Whilst the point estimate reported a 10% increase in mortality risk on average in the CRP group, this was low certainty, imprecise evidence (RR 1.10, 95%CI 0.69–1.76). Two trials (1847 patients) reported a comparison of PCT‐ with CRP‐guided antibiotic discontinuation protocols, suggesting a 1% decrease in mortality risk in the PCT group with moderate certainty evidence, although the confidence interval included the possibility of no effect (RR 0.99, 95%CI 0.94–1.05). Corresponding forest plots are in online Supporting Information Figures S24 and S25.

In total, 11 studies (4270 patients) reported recurrence of infection. There was low certainty evidence about whether PCT‐guided antibiotic discontinuation protocols differ from standard care in terms of infection recurrence (RR 1.27, 95%CI 0.96–1.68, χ^2^ = 6.40, I^2^ = 0%; online Supporting Information Figure S26).

Seven randomised controlled trials (4083 patients) reported on the incidence of secondary infections or superinfections. There was low certainty evidence regarding a difference in the risk of secondary infections between PCT‐guided antibiotic discontinuation protocols and standard care (RR 0.89, 95%CI 0.53–1.49, χ^2^ = 12.37, I^2^ = 51.5%; online Supporting Information Figure S27).

Thirteen randomised controlled trials (5699 patients) reported on duration of hospital stay. Very low certainty evidence suggested that patients receiving PCT‐guided antibiotic discontinuation protocols may experience a reduction in hospital stay of 1.0 days compared with those receiving standard care (95%CI ‐3.8–1.9, χ^2^ = 61.29, I^2^ = 80.4; online Supporting Information Figure S28).

Fifteen randomised controlled trials (5562 patients) reported on duration of ICU stay. Low certainty evidence suggested that PCT‐guided antibiotic discontinuation protocols may reduce ICU stay by 1.2 days compared with standard care (95%CI ‐3.7–1.3, χ^2^ = 95.06, I^2^ = 85.3%; online Supporting Information Figure S29).

It was unclear whether there is a difference between CRP‐guided antibiotic discontinuation protocols and standard care in: infection recurrence; secondary infections or superinfections; duration of hospital and ICU stay; and between PCT‐ and CRP‐guided antibiotic discontinuation protocols. For all comparisons, the evidence was of very low to low certainty; online Supporting Information Tables S11 and S12 give all results and Figures S30–S36 give all forest plots.

## Discussion

We found that PCT‐guided discontinuation protocols reduced antibiotic therapy by, on average, 2.0 days (moderate certainty evidence) compared with standard care. These results align with findings from previous reviews, which have reported that PCT‐guided protocols consistently reduce the duration of antibiotic therapy in patients who are critically ill with sepsis by 1–3 days across various populations, including primary care [62, 63] and critical care [9, 11, 64, 65, 66]. However, many of these reviews combined heterogeneous PCT‐informed antibiotic decision protocols, such as initiation, intensification and discontinuation protocols [9, 64, 66, 67], making it difficult to isolate the effect of discontinuation protocols alone. This is an important limitation (that we have mitigated), as while discontinuation protocols are generally associated with reduced antibiotic use and improved outcomes, some evidence suggests initiation or intensification protocols may increase antibiotic exposure and morbidity in critically ill populations [67, 68].

Our meta‐regression findings identified baseline SOFA scores as explaining a significant amount of the between‐study heterogeneity for PCT‐guided protocols compared with standard care. This suggests that sepsis severity could be a significant moderator, with PCT‐guided discontinuation potentially having less impact on reducing duration of antibiotic therapy in patients who are more severely ill (when judged clinically). This finding aligns with some previous review findings [64, 67]. Potential explanations may be that clinicians in high‐acuity settings are generally less willing to adhere to PCT algorithms or there is more caution in antibiotic discontinuation in patients perceived as more seriously ill [69]. Whilst these findings are from a meta‐regression and need to be treated with caution given their observational nature [70], they highlight the importance of considering how clinical judgement and PCT‐guided protocols are intercalated in decision‐making during implementation activities.

We also found that PCT‐guided antibiotic discontinuation protocols may reduce the mortality risk by 5% on average, both in the short and long term, compared with standard care (moderate certainty evidence). Earlier reviews either reported no difference in mortality [10, 71] or reported a significant relative reduction of 13% and 18% in mortality risk among patients allocated to the PCT discontinuation group compared with standard care [9, 67]. These differences may be attributed to the sample size and specific protocols synthesised, as our review encompassed a larger sample size and focused on antibiotic discontinuation protocols (not including studies that involved both initiation and discontinuation protocols for antibiotic therapy).

The effects on sepsis recurrence, secondary infections/superinfections and duration of hospital or ICU stay remain inconclusive for PCT‐guided protocols compared with standard care, CRP compared with standard care, and PCT‐guided care compared with CRP‐guided care (low to very low certainty evidence).

It is important to recognise that PCT and CRP are not specific markers of infection alone and have diagnostic limitations in the setting of unselected acute inflammatory illness. For example, PCT is more specific for bacterial infections than CRP [11], but may be elevated in the context of non‐infectious states such as trauma or surgery, complicating interpretation [72]. Similarly, CRP is a non‐specific marker of inflammation that may be elevated in autoimmune diseases, malignancies and other inflammatory states [73]. In addition, PCT concentrations are known to increase earlier and normalise more rapidly than CRP when tracking inflammation caused by bacterial infection and the responses to treatment [74]. These limitations highlight the need to integrate biomarker‐guided protocols with clinical judgement, as no single biomarker captures the complex patient response in sepsis fully or accounts for patient heterogeneity [72].

This review has several strengths that enhance the reliability and clinical applicability of our findings. Our review incorporated the most recent trial data, including a large randomised controlled trial published in 2025, the only included study assessed as having a low overall risk of bias due to the trial methods designed to reduce performance bias [14]. In other studies, outcome assessments were conducted either by unblinded health professionals or by blinded research staff, while unblinded clinicians delivered the intervention. Our review also followed the latest Cochrane Rapid Review guidance for assessing risk of bias and certainty of evidence, ensuring methodological rigour. Unlike previous reviews that pooled heterogeneous biomarker protocols and algorithms, this review focused specifically on antibiotic discontinuation protocols, allowing for more clinically actionable insights. As a result, the findings are well‐positioned to inform up‐to‐date antimicrobial stewardship practices, particularly in critical care settings.

The review has some limitations. The database search was restricted to studies published between 2005 and 2025 to maximise useful results. This approach, however, aligns with current guidance for updating reviews [20] and was mitigated by comprehensive forward and backward citation screening. Several pre‐specified subgroup analyses (e.g. intervention adherence and infection site) could not be conducted due to insufficient studies, limiting exploration of clinically important sources of heterogeneity. In addition, subgroup analyses should be interpreted with caution, as guidance suggests a minimum of 10 studies for each predictor, with a large number of studies yielding more power [70]. Several included studies were at high risk of bias, particularly for the outcomes of duration of antibiotic therapy and hospital/ICU stay (due to lack of blinding), and infection recurrence (due to inconsistent definitions and blinding issues). Although the ADAPT‐Sepsis study was assessed carefully and independently (as reviewers SR, SG, CS and JD were not involved in the trial execution), we acknowledge that these reviewers do work with ADAPT‐Sepsis authors. Nonetheless, methodological rigour and no significant differences in the primary outcomes between high and low/some risk of bias studies support the robustness of our conclusions. The included studies primarily involved older, predominantly male patients with community‐acquired sepsis, commonly involving respiratory, abdominal or urinary infections, which may limit generalisability to other critically ill populations. However, recent evidence from the ADAPT‐Sepsis trial suggests that factors such as infection site (e.g. urinary tract and intra‐abdominal) and sepsis acquisition (e.g. community‐ or hospital‐acquired) do not significantly affect the outcomes of PCT‐guided discontinuation protocols [14]. This subgroup analysis indicates potential applicability across diverse clinical subgroups. Nonetheless, this review was unable to assess these subgroup effects due to limited international data. Furthermore, most studies in this review were conducted in high‐income settings, limiting applicability to low‐resource settings internationally.

In conclusion, we found evidence that PCT‐guided antibiotic discontinuation protocols for patients who are critically ill with sepsis safely reduce the duration of antibiotic treatment. Evidence for CRP‐guided protocols remains inconclusive due to the very limited evidence available. However, given the substantial heterogeneity and reduced effect size in patients who were more severely ill, careful consideration should be used when applying PCT‐guided discontinuation protocols in this population. These protocols should not be interpreted as a mortality‐reducing intervention but rather a safe and effective tool for optimising antibiotic exposure.

## Supporting information


**Table S1.** Summary baseline characteristics of the included studies, full version.
**Table S2.** Baseline characteristics of the included studies.
**Table S3.** Biomarker discontinuation protocols for all included studies.
**Table S4.** Summary of the overall risk of bias for each outcome: procalcitonin‐guided vs. standard care.
**Table S5.** Egger's test results for the primary outcomes: procalcitonin‐guided vs. standard care.
**Table S6.** Sensitivity analyses: procalcitonin‐guided vs. standard care.
**Table S7.** Subgroup analyses: procalcitonin‐guided vs. standard care.
**Table S8.** Subgroup analysis of discontinuation protocols: procalcitonin‐guided vs. standard care.
**Table S9.** Subgroup analysis of discontinuation protocols: procalcitonin‐guided vs. standard care (excluding Nazer et al.).
**Table S10.** Univariable meta‐regression of baseline SOFA and APACHE scores as moderators of duration of antibiotic therapy and mortality.
**Table S11.** C‐reactive protein‐guided compared with standard care for adult patients critically ill with sepsis.
**Table S12.** Procalcitonin‐guided compared with CRP‐guided care for adult patients critically ill with sepsis.

**Figure S1.** MEDLINE database search strategy.
**Figures S2–S4.** Risk of bias judgements for duration of antibiotic therapy: procalcitonin‐guided compared with standard care; CRP‐guided compared with standard care; and procalcitonin‐guided compared with CRP‐guided care.
**Figures S5–S7.** Risk of bias judgements for mortality: procalcitonin‐guided compared with standard care; CRP‐guided compared with standard care; and procalcitonin‐guided compared with CRP‐guided care.
**Figures S8–S10.** RoB2 judgements for infection recurrence: procalcitonin‐guided compared with standard care; CRP‐guided compared with standard care; and procalcitonin‐guided compared with CRP‐guided care.
**Figures S11 and S12.** RoB2 judgements for secondary infections or superinfections: procalcitonin‐guided compared with standard care; and CRP‐guided compared with standard care.
**Figures S13–S15.** RoB2 judgements for duration of hospital stay: procalcitonin‐guided compared with standard care; CRP‐guided compared to standard care; and procalcitonin‐guided compared with CRP‐guided care.
**Figures S16–S18.** RoB2 judgements for duration of ICU stay: procalcitonin‐guided compared with standard care; CRP‐guided compared with standard care; and procalcitonin‐guided compared with CRP‐guided care.
**Figure S19.** Studies comparing procalcitonin‐guided with standard care for the outcome of duration of antibiotic therapy.
**Figure S20.** Duration of antibiotic therapy in patients receiving CRP‐guided compared with standard care.
**Figure S21.** Duration of antibiotic therapy in patients receiving procalcitonin‐guided compared with CRP‐guided care.
**Figure S22.** Mortality (long‐term) in patients receiving procalcitonin‐guided compared with standard care.
**Figure S23.** Studies comparing procalcitonin‐guided with standard care for the outcome of mortality (short‐term).
**Figure S24.** Mortality (short‐term) in patients receiving CRP‐guided compared with standard care.
**Figure S25.** Mortality (short‐term) in patients receiving procalcitonin‐guided compared with CRP‐guided care.
**Figure S26.** Infection recurrence in patients receiving procalcitonin‐guided compared with standard care.
**Figure S27.** Secondary infections or superinfections in patients receiving procalcitonin‐guided compared with standard care.
**Figure S28.** Duration of hospital stay in patients receiving procalcitonin‐guided compared with standard care.
**Figure S29.** Duration of ICU stay in patients receiving procalcitonin‐guided compared with standard care.
**Figure S30.** Infection recurrence in patients receiving CRP‐guided compared with standard care.
**Figure S31.** Infection recurrence in patients receiving procalcitonin‐guided compared with CRP‐guided care.
**Figure S32.** Secondary infections or superinfections in patients receiving CRP‐guided compared with standard care.
**Figure S33.** Duration of hospital stay in patients receiving CRP‐guided compared with standard care.
**Figure S34.** Duration of hospital stay in patients receiving procalcitonin‐guided compared with CRP‐guided care.
**Figure S35.** Duration of ICU stay in patients receiving CRP‐guided compared with standard care.
**Figure S36.** Duration of ICU stay in patients receiving procalcitonin‐guided compared with CRP‐guided care.

**Appendix S1.** Extended methods.

## References

[1] Singer M , Deutschman CS , Seymour CW , et al. The third international consensus definitions for sepsis and septic shock (Sepsis‐3). JAMA 2016; 315: 801–810. 10.1001/jama.2016.0287. 26903338 PMC4968574

[2] Rudd KE , Johnson SC , Agesa KM , et al. Global, regional, and national sepsis incidence and mortality, 1990–2017: analysis for the global burden of disease study. Lancet 2020; 395: 200–211. 10.1016/S0140-6736(19)32989-7. 31954465 PMC6970225

[3] Rhodes A , Evans LE , Alhazzani W , et al. Surviving sepsis campaign: international guidelines for management of sepsis and septic shock: 2016. Intensive Care Med 2017; 43: 304–377. 10.1007/s00134-017-4683-6. 28101605

[4] Evans L , Rhodes A , Alhazzani W , et al. Surviving sepsis campaign: international guidelines for management of sepsis and septic shock 2021. Intensive Care Med 2021; 47: 1181–1247. 10.1007/s00134-021-06506-y. 34599691 PMC8486643

[5] Baggs J , Jernigan JA , Halpin AL , Epstein L , Hatfield KM , McDonald LC . Risk of subsequent sepsis within 90 days after a hospital stay by type of antibiotic exposure. Clin Infect Dis 2018; 66: 1004–1012. 10.1093/cid/cix947. 29136126 PMC7909479

[6] Teshome BF , Vouri SM , Hampton N , Kollef MH , Micek ST . Duration of exposure to antipseudomonal β‐lactam antibiotics in the critically ill and development of new resistance. Pharmacotherapy 2019; 39: 261–270. 10.1002/phar.2201. 30506852 PMC6507412

[7] Naghavi M , Vollset SE , Ikuta KS , et al. Global burden of bacterial antimicrobial resistance 1990–2021: a systematic analysis with forecasts to 2050. Lancet 2024; 404: 1199–1226. 10.1016/S0140-6736(24)01867-1. 39299261 PMC11718157

[8] Albrich W , Harbarth S , Albrich WC . Pros and cons of using biomarkers versus clinical decisions in start and stop decisions for antibiotics in the critical care setting. Intensive Care Med 2015; 41: 1739–1751. 10.1007/s00134-015-3978-8. 26194026

[9] Kubo K , Sakuraya M , Sugimoto H , et al. Benefits and harms of procalcitonin‐ or C‐reactive protein‐guided antimicrobial discontinuation in critically ill adults with sepsis: a systematic review and network meta‐analysis. Crit Care Med 2024; 52: e522–e534. 10.1097/CCM.0000000000006366. 38949476

[10] Pepper DJ , Sun J , Rhee C , Welsh J , Powers JH III , Danner RL , Kadri SS . Procalcitonin‐guided antibiotic discontinuation and mortality in critically ill adults: a systematic review and meta‐analysis. Chest 2019; 155: 1109–1118. 10.1016/j.chest.2018.12.029. 30772386 PMC6607427

[11] Prkno A , Wacker C , Brunkhorst FM , Schlattmann P . Procalcitonin‐guided therapy in intensive care unit patients with severe sepsis and septic shock – a systematic review and meta‐analysis. Crit Care 2013; 17: R291. 10.1186/cc13157. 24330744 PMC4056085

[12] Hellyer TP , Mantle T , McMullan R , Dark P . How to optimise duration of antibiotic treatment in patients with sepsis? BMJ 2020; 371: m4357. 10.1136/bmj.m4357. 33229405

[13] National Institute for Health and Care Excellence . Procalcitonin testing for diagnosing and monitoring sepsis (ADVIA Centaur BRAHMS PCT assay, BRAHMS PCT Sensitive Kryptor assay, Elecsys BRAHMS PCT assay, LIAISON BRAHMS PCT assay and VIDAS BRAHMS PCT assay) [DG18]. 2015. https://www.nice.org.uk/guidance/dg18 (accessed 14/10/2025).

[14] Dark P , Hossain A , McAuley DF , et al. Biomarker‐guided antibiotic duration for hospitalized patients with suspected sepsis: the ADAPT‐Sepsis randomized clinical trial. JAMA 2025; 333: 682–693. 10.1001/jama.2024.26458. 39652885 PMC11862976

[15] Shukeri W , Mat‐Nor M , Ralib A , Mazlan M , Hassan M . Point‐of‐care procalcitonin to guide the discontinuation of antibiotic treatment in the intensive care unit: a Malaysian randomised controlled trial. Malaysian J Med Health Sci 2022; 18: 65–71. 10.47836/mjmhs18.6.10.

[16] Schuetz P , Beishuizen A , Broyles M , et al. Procalcitonin (PCT)‐guided antibiotic stewardship: an international experts consensus on optimized clinical use. Clin Chem Lab Med 2019; 57: 1308–1318. 10.1515/cclm-2018-1181. 30721141

[17] Kien C , Daxenbichler J , Titscher V , et al. Effectiveness of de‐implementation of low‐value healthcare practices: an overview of systematic reviews. Implement Sci 2024; 19: 56. 10.1186/s13012-024-01384-6. 39103927 PMC11299416

[18] Garritty C , Hamel C , Trivella M , et al. Updated recommendations for the Cochrane rapid review methods guidance for rapid reviews of effectiveness. BMJ 2024; 384: e076335. 10.1136/bmj-2023-076335. 38320771

[19] Page MJ , McKenzie JE , Bossuyt PM , et al. The PRISMA 2020 statement: an updated guideline for reporting systematic reviews. BMJ 2021; 372: n71. 10.1136/bmj.n71. 33782057 PMC8005924

[20] Xu C , Ju K , Lin L , Jia P , Kwong JSW , Syed A , Furuya‐Kanamori L . Rapid evidence synthesis approach for limits on the search date: how rapid could it be? Res Synth Methods 2022; 13: 68–76. 10.1002/jrsm.1525. 34523791

[21] Haddaway NR , Grainger MJ , Gray CT . Citationchaser: a tool for transparent and efficient forward and backward citation chasing in systematic searching. Res Synth Methods 2022; 13: 533–545. 10.1002/jrsm.1563. 35472127

[22] Sterne JAC , Savović J , Page MJ , et al. Rob 2: a revised tool for assessing risk of bias in randomised trials. BMJ 2019; 366: l4898. 10.1136/bmj.l4898. 31462531

[23] McGuinness LA , Higgins JPT . Risk‐of‐bias VISualization (robvis): an R package and Shiny web app for visualizing risk‐of‐bias assessments. Res Synth Methods 2020; 12: 55–61. 10.1002/jrsm.1411. 32336025

[24] Monaghan TF , Agudelo CW , Rahman SN , Wein AJ , Lazar JM , Everaert K , Dmochowski RR . Blinding in clinical trials: seeing the big picture. Medicina (Kaunas) 2021; 57: 647. 10.3390/medicina57070647. 34202486 PMC8308085

[25] Deeks JJ , Higgins JPT , Altman DG , McKenzie JE , Veroniki AA . Chapter 10: analysing data and undertaking meta‐analyses. In: Higgins J , Thomas J , Chandler J , et al., eds. Cochrane Handbook for Systematic Reviews of Interventions Version 6.5, 2024. https://www.cochrane.org/authors/handbooks‐and‐manuals/handbook/current/chapter‐10 (accessed 26/11/2025).

[26] Balduzzi S , Rücker G , Schwarzer G . How to perform a meta‐analysis with R: a practical tutorial. Evid Based Ment Health 2019; 22: 153–160. 10.1136/ebmental-2019-300117. 31563865 PMC10231495

[27] Viechtbauer W . Conducting meta‐analyses in R with the metafor package. J Stat Softw 2010; 36: 1–48. 10.18637/jss.v036.i03.

[28] Harrer M , Cuijpers P , Furukawa TA , Ebert DD . Doing Meta‐Analysis with R: A Hands‐On Guide. Boca Raton, FL and London: Chapman & Hall/CRC Press, 2021.

[29] Baujat B , Mahé C , Pignon J‐P , Hill C . A graphical method for exploring heterogeneity in meta‐analyses: application to a meta‐analysis of 65 trials. Stat Med 2002; 21: 2641–2652. 10.1002/sim.1221. 12228882

[30] Abbas A , Hefnawy MT , Negida A . Meta‐analysis accelerator: a comprehensive tool for statistical data conversion in systematic reviews with meta‐analysis. BMC Med Res Methodol 2024; 24: 243. 10.1186/s12874-024-02356-6. 39425031 PMC11487830

[31] Wan X , Wang W , Liu J , Tong T . Estimating the sample mean and standard deviation from the sample size, median, range and/or interquartile range. BMC Med Res Methodol 2014; 14: 135. 10.1186/1471-2288-14-135. 25524443 PMC4383202

[32] Knapp G , Hartung J . Improved tests for a random effects meta‐regression with a single covariate. Stat Med 2003; 22: 2693–2710. 10.1002/sim.1482. 12939780

[33] Higgins JPT , Thompson SG , Deeks JJ , Altman DG . Measuring inconsistency in meta‐analyses. BMJ 2003; 327: 557–560. 10.1136/bmj.327.7414.557. 12958120 PMC192859

[34] Guyatt G , Oxman AD , Akl EA , et al. GRADE guidelines: 1. Introduction—GRADE evidence profiles and summary of findings tables. J Clin Epidemiol 2011; 64: 383–394. 10.1016/j.jclinepi.2010.04.026. 21195583

[35] ClinicaTrials.gov . NCT06395454. Usage of procalcitonin to reduce antibiotics duration in VAP in neurosurgical ICU. 2024. https://clinicaltrials.gov/study/NCT06395454 (accessed 15/10/2025).

[36] ClinicaTrials.gov . NCT05955612. Use of procalcitonin, a blood test to guide antibiotic therapy for sepsis in adults. 2024. https://www.clinicaltrials.gov/study/NCT05955612 (accessed 15/10/2025).

[37] ClinicaTrials.gov . NCT00415753. Procalcitonin as a marker of bacterial pneumonia. 2008. https://clinicaltrials.gov/study/NCT00415753 (accessed 15/10/2025).

[38] ClinicaTrials.gov . NCT01379547. Procalcitonin to shorten antibiotics duration in ICU patients. 2012. https://clinicaltrials.gov/study/NCT01379547 (accessed 15/10/2025).

[39] ClinicaTrials.gov . NCT01572831. PCT and clinical algorithm for determination of duration of antibiotics. 2015. https://clinicaltrials.gov/study/NCT01572831 (accessed 15/10/2025).

[40] ClinicaTrials.gov . NCT00987818. Procalcitonin guided versus conventional antibiotic therapy in patients with sepsis in the ICU. 2015. https://clinicaltrials.gov/study/NCT00987818 (accessed 15/10/2025).

[41] ClinicaTrials.gov . NCT00407147. Procalcitonin level to discontinue antibiotics on ICU patients with no obvious site of infection. 2012. https://clinicaltrials.gov/study/NCT00407147 (accessed 15/10/2025).

[42] ClinicaTrials.gov . NCT01018199. Procalcitonin versus C‐reactive protein to guide therapy in community acquired pneumonia. 2016. https://clinicaltrials.gov/study/NCT01018199 (accessed 15/10/2025).

[43] Nobre V , Harbarth S , Graf J‐D , Rohner P , Pugin J . Use of procalcitonin to shorten antibiotic treatment duration in septic patients: a randomized trial. Am J Respir Crit Care Med 2008; 177: 498–505. 10.1164/rccm.200708-1238OC. 18096708

[44] Schroeder S , Hochreiter M , Koehler T , Schweiger AM , Bein B , Keck FS , von Spiegel T . Procalcitonin (PCT)‐guided algorithm reduces length of antibiotic treatment in surgical intensive care patients with severe sepsis: results of a prospective randomized study. Langenbeck's Arch Surg 2009; 394: 221–226. 10.1007/s00423-008-0432-1. 19034493

[45] Hochreiter M , Kohler T , Schweiger AM , Keck FS , Bein B , von Spiegel T , Schroeder S . Procalcitonin to guide duration of antibiotic therapy in intensive care patients: a randomized prospective controlled trial. Crit Care 2009; 13: R83. 10.1186/cc7903. 19493352 PMC2717450

[46] Stolz D , Smyrnios NA , Eggimann P , et al. Procalcitonin for reduced antibiotic exposure in ventilator‐associated pneumonia: a randomised study. Eur Respir J 2009; 34: 1364–1375. 10.1183/09031936.00053209. 19797133

[47] Deliberato RO , Marra AR , Sanches PR , et al. Clinical and economic impact of procalcitonin to shorten antimicrobial therapy in septic patients with proven bacterial infection in an intensive care setting. Diagn Microbiol Infect Dis 2013; 76: 266–271. 10.1016/j.diagmicrobio.2013.03.027. 23711530

[48] Liu B , Li H , Lei Y , Zhao S , Sun M . Clinical significance of dynamic monitoring of procalcitonin in guiding the use of antibiotics in patients with sepsis in ICU. Zhonghua Wei Zhong Bing Ji Jiu Yi Xue 2013; 25: 690–693. 10.3760/cma.j.issn.2095-4352.2013.11.013. 24225216

[49] Shehabi Y , Sterba M , Garrett PM , et al. Procalcitonin algorithm in critically ill adults with undifferentiated infection or suspected sepsis. A randomized controlled trial. Am J Respir Crit Care Med 2014; 190: 1102–1110. 10.1164/rccm.201408-1483OC. 25295709

[50] Bloos F , Trips E , Nierhaus A , et al. Effect of sodium selenite administration and procalcitonin‐guided therapy on mortality in patients with severe sepsis or septic shock: a randomized clinical trial. JAMA Intern Med 2016; 176: 1266–1276. 10.1001/jamainternmed.2016.2514. 27428731

[51] de Jong E , van Oers JAH , Beishuizen A , et al. Efficacy and safety of procalcitonin guidance in reducing the duration of antibiotic treatment in critically ill patients: a randomised, controlled, open‐label trial. Lancet Infect Dis 2016; 16: 819–827. 10.1016/s1473-3099(16)00053-0. 26947523

[52] Mahmutaj D , Krasniqi S , Braha B , Limani D , Neziri B . The predictive role of procalcitonin on the treatment of intra‐abdominal infections. Open Access Maced J Med Sci 2017; 5: 909–914. 10.3889/oamjms.2017.194. 29362617 PMC5771293

[53] Xu X , Yan F , Yu J , Chen Q , Lin H , Zheng R . Efficacy and safety of procalcitonin guidance in reducing the duration of antibiotic treatment of sepsis patients. Zhonghua Yi Xue Za Zhi 2017; 97: 343–346. 10.3760/cma.j.issn.0376-2491.2017.05.005. 28219190

[54] Jeon K , Suh JK , Jang EJ , et al. Procalcitonin‐guided treatment on duration of antibiotic therapy and cost in septic patients (PRODA): a multi‐center randomized controlled trial. J Korean Med Sci 2019; 34: e110. 10.3346/jkms.2019.34.e110. 30977312 PMC6460106

[55] Mazlan MZ , Ismail MAH , Ali S , Salmuna ZN , Shukeri WFWM , Omar M . Efficacy and safety of the point‐of‐care procalcitonin test for determining the antibiotic treatment duration in patients with ventilator‐associated pneumonia in the intensive care unit: a randomised controlled trial. Anaesthesiol Intensive Ther 2021; 53: 207–214. 10.5114/ait.2021.104300. 34006044 PMC10158486

[56] Vishalashi SG , Gupta P , Verma PK . Serum procalcitonin as a biomarker to determine the duration of antibiotic therapy in adult patients with sepsis and septic shock in intensive care units: a prospective study. Indian J Crit Care Med 2021; 25: 507–511. 10.5005/jp-journals-10071-23802. 34177168 PMC8196370

[57] Kyriazopoulou E , Liaskou‐Antoniou L , Adamis G , et al. Procalcitonin to reduce long‐term infection‐associated adverse events in sepsis. A randomized trial. Am J Respir Crit Care Med 2021; 203: 202–210. 10.1164/rccm.202004-1201OC. 32757963 PMC7874409

[58] Nazer LH , Awad W , Thawabieh H , et al. Procalcitonin‐guided management and duration of antibiotic therapy in critically ill cancer patients with sepsis (Pro‐Can study): a randomized controlled trial. Crit Care Explor 2024; 6: e1173. 10.1097/CCE.0000000000001173. 39431961 PMC11495690

[59] Liu Y , Yang W , Wei J . Guiding effect of serum procalcitonin (PCT) on the antibiotic application to patients with sepsis. Iran J Public Health 2017; 46: 1535–1539. 29167772 PMC5696693

[60] Borges I , Carneiro R , Bergo R , et al. Duration of antibiotic therapy in critically ill patients: a randomized controlled trial of a clinical and C‐reactive protein‐based protocol versus an evidence‐based best practice strategy without biomarkers. Crit Care 2020; 24: 1–11. 10.1186/s13054-020-02946-y. 32487263 PMC7266125

[61] Oliveira CF , Botoni FA , Oliveira CRA , Silva CB , Pereira HA , Serufo JC , Nobre V . Procalcitonin versus C‐reactive protein for guiding antibiotic therapy in sepsis: a randomized trial. Crit Care Med 2013; 41: 2336–2343. 10.1097/CCM.0b013e31828e969f. 23921272

[62] Heilmann E , Gregoriano C , Annane D , et al. Duration of antibiotic treatment using procalcitonin‐guided treatment algorithms in older patients: a patient‐level meta‐analysis from randomized controlled trials. Age Ageing 2021; 50: 1546–1556. 10.1093/ageing/afab078. 33993243 PMC8437072

[63] Papp M , Kiss N , Baka M , et al. Procalcitonin‐guided antibiotic therapy may shorten length of treatment and may improve survival‐a systematic review and meta‐analysis. Crit Care 2023; 27: 394. 10.1186/s13054-023-04677-2. 37833778 PMC10576288

[64] Wirz Y , Meier MA , Bouadma L , et al. Effect of procalcitonin‐guided antibiotic treatment on clinical outcomes in intensive care unit patients with infection and sepsis patients: a patient‐level meta‐analysis of randomized trials. Crit Care 2018; 22: 191. 10.1186/s13054-018-2125-7. 30111341 PMC6092799

[65] Zhang T , Wang Y , Yang Q , et al. Procalcitonin‐guided antibiotic therapy in critically ill adults: a meta‐analysis. BMC Infect Dis 2017; 17: 1–11. 10.1186/s12879-017-2622-3. 28738787 PMC5525369

[66] Arulkumaran N , Khpal M , Tam K , Baheerathan A , Corredor C , Singer M . Effect of antibiotic discontinuation strategies on mortality and infectious complications in critically ill septic patients: a meta‐analysis and trial sequential analysis. Crit Care Med 2020; 48: 757–764. 10.1097/CCM.0000000000004267. 32191414

[67] Peng F , Chang W , Xie JF , Sun Q , Qiu HB , Yang Y . Ineffectiveness of procalcitonin‐guided antibiotic therapy in severely critically ill patients: a meta‐analysis. Int J Infect Dis 2019; 85: 158–166. 10.1016/j.ijid.2019.05.034. 31229612

[68] Soni NJ , Samson DJ , Galaydick JL , Vats V , Huang ES , Aronson N , Pitrak DL . Procalcitonin‐guided antibiotic therapy: a systematic review and meta‐analysis. J Hosp Med 2013; 8: 530–540. 10.1002/jhm.2067. 23955852

[69] Schuetz P , Muller B , Christ‐Crain M , et al. Procalcitonin to initiate or discontinue antibiotics in acute respiratory tract infections. Cochrane Database Syst Rev 2012; 2012: CD007498. 10.1002/14651858.CD007498.pub2. 22972110 PMC6464976

[70] Spineli LM , Pandis N . Problems and pitfalls in subgroup analysis and meta‐regression. Am J Orthod Dentofacial Orthop 2020; 158: 901–904. 10.1016/j.ajodo.2020.09.001. 33250104

[71] Andriolo BN , Andriolo RB , Salomao R , Atallah AN . Effectiveness and safety of procalcitonin evaluation for reducing mortality in adults with sepsis, severe sepsis or septic shock. Cochrane Database Syst Rev 2017; 1: CD010959. 10.1002/14651858.CD010959.pub2. 28099689 PMC6353122

[72] Farooq A , Colón‐Franco JM . Procalcitonin and its limitations: why a biomarker's best isn't good enough. J Appl Lab Med 2019; 3: 716–719. 10.1373/jalm.2017.025916. 31639738

[73] Lippi G , Meschi T , Cervellin G . Inflammatory biomarkers for the diagnosis, monitoring and follow‐up of community‐acquired pneumonia: clinical evidence and perspectives. Eur J Intern Med 2011; 22: 460–465. 10.1016/j.ejim.2011.02.023. 21925053

[74] Meisner M . Pathobiochemistry and clinical use of procalcitonin. Clin Chim Acta 2002; 323: 17–29. 10.1016/s0009-8981(02)00101-8. 12135804

