# Supplementary material for: Clinical effectiveness of procalcitonin‐ or C‐reactive protein‐guided antibiotic discontinuation protocols for adult patients who are critically ill with sepsis: a rapid systematic review and meta‐analysis
Source: Anaesthesia. 2026 Jan 8;81(4):556–69. doi: 10.1111/anae.70109 (PMC12973355; doi:10.1111/anae.70109)
Supplement: Supplementary file 1 — Table S1. Summary baseline characteristics of the included studies, full version. Table S2. Baseline characteristics of the included studies. Table S3. Biomarker discontinuation protocols for all included studies. Table S4. Summary of the overall risk of bias for each outcome: procalcitonin‐guided vs. standard care. Table S5. Egger's test results for the primary outcomes: procalcitonin‐guided vs. standard care. Table S6. Sensitivity analyses: procalcitonin‐guided vs. standard care. Table S7. Subgroup analyses: procalcitonin‐guided vs. standard care. Table S8. Subgroup analysis of discontinuation protocols: procalcitonin‐guided vs. standard care. Table S9. Subgroup analysis of discontinuation protocols: procalcitonin‐guided vs. standard care (excluding Nazer et al.). Table S10. Univariable meta‐regression of baseline SOFA and APACHE scores as moderators of duration of antibiotic therapy and mortality. Table S11. C‐reactive protein‐guided compared with standard care for adult patients critically ill with sepsis. Table S12. Procalcitonin‐guided compared with CRP‐guided care for adult patients critically ill with sepsis. [file ANAE-81-556-s001.docx]

| **Table S1**: Summary baseline characteristics of the included studies, full version | | | |  |
| --- | --- | --- | --- | --- |
|  | **Summary statistics,  PCT vs SOC**  **(19 studies)** | **Summary statistics,  CRP vs SOC**  **(2 studies)** | **Summary statistics,  PCT vs CRP,**  **(2 studies)** | |
| ***Publication types (n)*** | | | |  |
| Trial protocol | 7 |  | 1 |  |
| Journal paper | 19 | 2 | 2 |  |
| ***Countries (n)*** | | | |  |
| Australia | 1 | 0 | 0 |  |
| Brazil | 2 | 1 | 1 |  |
| China | 3 | 0 | 0 |  |
| Greece | 1 | 0 | 0 |  |
| Germany | 3 | 0 | 0 |  |
| India | 1 | 0 | 0 |  |
| Jordan | 1 | 0 | 0 |  |
| Korea | 1 | 0 | 0 |  |
| Kosovo | 1 | 0 | 0 |  |
| Malaysia | 1 | 0 | 0 |  |
| Netherlands | 1 | 0 | 0 |  |
| Switzerland | 2 | 0 | 0 |  |
| UK | 1 | 1 | 1 |  |
| ***Sample size at baseline (n (%))*** | | | |  |
| Total | 6415 | 1972 | 1936 |  |
| Intervention | 3209 (50%) | 988 (50%) | 967 (50%) |  |
| Control | 3206 (50%) | 984 (50%) | 969 (50%) |  |
| ***Sex (n (%))*** | | | |  |
| Male | 3797 (59%) | 1134 (59%) | 777 (40%) |  |
| Female | 2586 (41%) | 799 (41%) | 1150 (60%) |  |
| ***Sepsis severity (n (%))*** | | | |  |
| Septic shock | 2560 (46%) | 944 (48%) | 958 (50%) |  |
| Sepsis | 2958 (54%) | 1012 (52%) | 969 (50%) |  |
| ***Sepsis infection site (n (%))*** | | | |  |
| Lungs | 3000 (47%) | 956 (40%) | 941 (40%) |  |
| Abdomen | 1312 (21%) | 435 (18%) | 440 (18%) |  |
| Urinary tract | 617 (10%) | 247 (10%) | 242 (10%) |  |
| Skin and soft tissue | 290 (5%) | 166 (7%) | 142 (6%) |  |
| Blood stream | 251 (4%) | 174 (7%) | 174 (7%) |  |
| Catheter related | 51 (1%) | 30 (1%) | 28 (1%) |  |
| CNS | 110 (2%) | 54 (2%) | 63 (3%) |  |
| Eye, nose and throat | 62 (1%) | 48 (2%) | 38 (2%) |  |
| Thoracic or surgical wound | 85 (2%) | - | - |  |
| Unknown or other | 616 (10%) | 297 (12%) | 312 (13%) |  |
| ***Place of acquired sepsis infection (n (%))*** | | | |  |
| Community associated | 2267 (61%) | 1234 (68%) | 1264 (69%) |  |
| Hospital or healthcare-associated | 1057 (29%) | 577 (32%) | 580 (31%) |  |
| ICU associated | 389 (10%) | - | - |  |
| ***Outcomes (n)*** | | | |  |
| Antibiotic duration | 19 | 2 | 2 |  |
| Mortality | 18 | 2 | 2 |  |
| Infection recurrence | 12 * | 2 | 2 |  |
| Secondary infections or superinfections | 7 | 2 | 1 |  |
| Duration of intensive care stays | 15 | 2 | 2 |  |
| Duration of hospital stays | 13 | 2 | 2 |  |
| *Notes*: PCT = Procalcitonin; SOC = standard of care; CRP = C-Reactive Protein.  * For infection recurrence, 12 studies were identified, and descriptive data was extracted from all studies, but the effect size was generated based on data from 11 studies as one study [54] included zero event data. | | | |  |

| T**able S2:** Baseline characteristics of the included studies | | | | | | | | |
| --- | --- | --- | --- | --- | --- | --- | --- | --- |
| **Author (s), year** | **Country** | **Patient eligibility criteria, (sepsis definition)** | **Sample size at baseline (n)** | **Intensive care setting** | **Mean age**  **(SD)** | **Males  (n, %)** | **Mean SOFA**  **(SD)** | **Mean APACHE (SD)** |
| **PCT (intervention) vs SOC (control)** | | | | | | | | |
| Nobre et al. (2008) [43] | Switzerland | Adult patients who developed severe sepsis or septic shock during ICU stay, meeting the sepsis definition (Sepsis-1). | I: 39 C: 40 | Mixed medical and surgical patients ICU | I: 64.30 (13.60) C: 65.80 (15.00) | I: 27 (69%)  C: 27 (68%) | I: 5.90 (3.30) C: 6.70 (2.90) |  |
| Hochreiter et al. (2009) [45] | Germany | All patients requiring antibiotic therapy based on confirmed or highly suspected bacterial infections and meeting at least two concomitant SIRS criteria (Sepsis-1) | I: 57 C: 53 | Surgical ICU | I: 67.30 (14.40)  C: 66.60 (15.50) | I: 29 (51%)  C: 29 (55%) |  |  |
| Schroeder et al. (2009) [44] | Germany | Patients after abdominal surgery and after the start of antibiotic treatment, with the diagnosis of severe sepsis, who were admitted to the ICU (Sepsis-1). | I: 14 C: 13 | ICU | I: 69.30 (10.60) C: 68.40 (13.70) | I: 8 (57%)  C: 7 (54%) | I: 7.30 (3.50) C: 8.30 (4.20) |  |
| Stolz et al. (2009) [46] | Switzerland | Adult ICU patients intubated for mechanical ventilation for 48 hours or more (highly suspected sepsis) ** | I: 51 C: 50 | Medical and surgical ICU, 7 ICUs | I: 53.75 (14.89)* C: 54.75 (14.49)* | I: 38 (75%)  C: 37 (74%) | I: 7.30 (3.40) C: 8.20 (3.40) |  |
| Deliberato et al. (2013) [47] | Brazil | Adult critically ill patients admitted with suspected sepsis, severe sepsis, or septic shock, as well as patients who developed suspected sepsis, severe sepsis, or septic shock during their ICU stay (Sepsis-1). | I: 42 C: 39 | Medical-surgical ICU | I: 68.00 (21.00) C: 62.00 (19.00) | I: 24 (57 %)  C: 21 (54%) | I: 6.29 (2.85) C: 5.38 (3.33) |  |
| Liu et al. (2013) [48] | China | Suspected sepsis (not specified). | I: 42 C: 40 | ICU | I: 54.90 (13.80) C: 53.40 (12.20) | I: 20 (48%) C: 18 (45%) |  | I: 21.60 (4.30) C: 18.50 (3.60) |
| Shehabi et al. (2014) ^1^ [49] | Australia | Adult patients admitted to the ICU within the previous 72 hours, receiving parenteral and/or enteral antibiotics for a suspected bacterial infection (meeting two or more SIRS criteria) and expected to remain in the ICU for longer than 24 hours (Sepsis-2). | I: 196 C: 198 | 11 ICUs | I: 63.10 (14.90) C: 65.80 (15.50) | I: 93 (47%) C: 119 (60%) | I: 6.00 (4.48)* C: 5.67 (3.73)* | I: 21.20 (7.80) C: 20.90 (7.10) |
| Bloos et al. (2016) ^1^ [50] | Germany | Adults with severe sepsis or septic shock (termed severe sepsis) beginning no later than 24 hours before randomisation (Sepsis-1). | I: 552 C: 537 | 33 multidisciplinary ICU | Combined sample:  65.70 (13.70) | Combined sample:  691 (64%) | I: 9.90 (3.35)* C: 10.10 (3.3)* | I: 23.90 (7.55)* C: 24.55 (7.64)* |
| de Jong et al. (2016) ^1^ [51] | Netherlands | Adult patients admitted to the ICU who received their first dose of antibiotics within 24 hours before inclusion in the study for an assumed or proven infection.  (Sepsis-2). | I: 761 C: 785 | 15 hospital ICUs | I: 64.67 (15.60)* C: 65.67 (13.37)* | I: 464 (61%) C: 470 (60%) | I: 6.00 (4.46)* C: 6.33 (3.71)* | I: 72.00 (29.71)* C: 73.67 (29.71)* |
| Liu et al., (2017) [59] | China | Adult patients with bacterially infected sepsis diagnosed in the clinic and the ICU stay time was no more than 72 hours (not specified). | I: 49 C: 49 | Renmin Hospital of Wuhan University | I: 66.38 (9.32) C: 65.22 (10.13) | I: 27 (55%) C: 29 (58%) |  |  |
| Mahmutaj et al. (2017) [52] | Kosovo | Adult patients diagnosed with acute abdomen with Systemic Inflammatory Response Syndrome (SIRS), who underwent urgent surgical intervention and an Index Manheim Peritonitis score > 10 points (highly suspected sepsis) ** | I: 50 C: 50 | University Clinical Hospital ^2^ | Combined sample:  43.20 (18.50) | I: 34 (68%) C: 31 (62%) |  |  |
| Xu et al. (2017) [53] | China | Adults with suspected or confirmed sepsis, receiving antibiotics within 24 hours, and expected to stay in the ICU for at least 24 hours (not specified). | I: 79 C: 77 | ICU | I: 66.80 (9.10) C: 64.90 (8.80) | I: 45 C: 44 |  | I: 22.70 (4.70) C: 19.90 (4.20) |
| Jeon et al. (2019) ^1^ [54] | Korea | Adult patients, enrolled within 24 hours of admission to the ICU with suspected severe sepsis or septic shock, and expected to remain in the ICU for longer than 48 hours if they had received antibiotics for less than 48 hours (not specified). | I: 23 C: 29 | 4 medical ICUs | I: 68.33 (11.06)* C: 70.00 (10.92)* | I: 8 (33%) C: 14 (48%) | I: 9.67 (7.11)* C: 9.00 (3.12)* | I: 27.67 (16.59)* C: 22.67 (11.70)* |
| Mazlan et al. (2021) ^1^ [55] | Malaysia | Adult patients ventilated for more than 48 hours who had CPIS scores greater than five (highly suspected sepsis) ** | I: 43 C: 42 | 4 ICUs | I: 49.07 (17.08) C: 53.12 (16.49) | I: 27 (63%) C: 19 (45%) | I: 8.05 (3.14) C: 8.02 (2.86) | I: 18.77 (6.23) C: 18.50 (4.61) |
| Kyriazopoulou et al. (2021) ^1^ [57] | Greece | Adults hospitalised with LRTIs (community, hospital-acquired, or ventilator-associated), acute pyelonephritis, or primary bloodstream infection and meeting the sepsis definition (sepsis-3). | I: 131 C: 125 | 7 internal departments of internal medicine ^3^ | I: 79.60 (9.80) C: 78.00 (13.10) | I: 52 (41%)  C: 62 (46%) | I: 4.10 (2.10) C: 4.10 (2.20) | I: 13.00 (4.60) C: 13.30 (4.70) |
| Vishalashi et al. (2021) [56] | India | Adult patients with sepsis or septic shock, based on the sepsis three criteria, who were admitted to ICUs within 24 hours (sepsis-3). | I: 45 C: 45 | Mixed adult ICU | I: 42.89 (16.83) C: 46.78 (16.22) | I: 22 (49%) C: 29 (64%) | I: 4.18 (3.45) C: 4.80 (2.47) | I: 18.07 (6.67) C: 17.40 (5.53) |
| Shukeri et al. (2022) [15] | Malaysia | Adults with suspected or confirmed bacterial infections on admission to or during their stay in ICUs (not specified). | I: 40 C: 40 | ICU | I: 53.00 (18.00) C: 53.00 (18.00) | I: 25 (63%) C: 18 (45%) | I: 5.00 (3.84)* C: 4.67 (3.08)* | I: 12.00 (10.00)* C: 14.00 (8.46)* |
| Nazer et al. (2024) ^1^ [58] | Jordan | Adult patients with cancer who were started on antibiotics for the treatment of sepsis, upon ICU admission or during their ICU stay, and were expected to remain in the ICU for greater than or equal to 48 hours, meeting the sepsis three definition (sepsis-3). | I: 77 C: 76 | 2 ICUs | I: 59.33 (14.35)* C: 60.67 (11.34)* | I: 54 (70%) C: 49 (65%) | I: 7.67 (3.02)* C: 7.00 (3.02)* | I: 20.33 (6.80)* C: 21.67 (6.80)* |
| Dark et al. (2025) [14] ^1^ | UK | Adult patients admitted to either a critical care or ICU whose intravenous antibiotics for suspected sepsis had been initiated within 24 hours and were likely to be continued for at least 72 hours, meeting the criteria for sepsis (sepsis-3). | I: 918 C: 918 | 41 ICUs | I: 60.60 (15.20) C: 59.80 (15.30) | I: 561 (61%)  C: 544 (60%) | I: 7.00 (2.97)* C: 7.00 (2.97)* | I: 17.50 (6.50) C: 17.20 (6.50) |
| **CRP (intervention) vs SOC (control)** | | | | | | | | |
| Borges et al. (2020) ^1^ [60] | Brazil | Adult patients admitted to ICUs, with clinical suspicion or microbiological confirmation of infection, and the prospect of an ICU stay longer than 24h (sepsis-3). | I: 64 C: 66 | 2 ICUs | I: 60.20 (14.00) C: 57.00 (17.30) | I: 34 (53%) C: 34 (52%) | I: 6.67 (3.79)* C: 6.67 (3.03)* | I: 18.33 (6.83)* C: 16.67 (6.06)* |
| Dark et al. (2025) ^1^ [14] | UK | Adult patients admitted to either a critical care or ICU whose intravenous antibiotics for suspected sepsis had been initiated within 24 hours and were likely to be continued for at least 72 hours, meeting the sepsis definition (sepsis-3). | I: 924 C: 918 | 41 ICUs | I: 60.30 (15.60) C: 59.80 (15.30) | I: 522 (60%)  C: 544 (60%) | I: 7.00 (2.97)* C: 7.00 (2.97)* | I: 17.30 (6.40) C: 17.20 (6.50) |
| **PCT (intervention) vs CRP (control)** | | | | | | | | |
| Oliveira et al. (2013) ^1^ [61] | Brazil | Adult patients with suspected severe sepsis or septic shock (sepsis 1/2). | I: 49 C: 45 | ICU | I: 59.60 (13.3) C: 59.60 (18.5) | I: 31 (63%) C: 26 (58%) | I: 7.50 (3.82)* C: 7.00 (4.59)* | I: 20.83 (11.07)* C: 20.00 (7.66)* |
| Dark et al. (2025) ^1^ [14] | UK | Adult patients admitted to either a critical care or ICU whose intravenous antibiotics for suspected sepsis had been initiated within 24 hours and were likely to be continued for at least 72 hours, meeting sepsis criteria (sepsis-3). | I: 918 C: 924 | 41 ICUs | I: 60.60 (15.2) C: 60.30 (15.6) | I: 561 (61%) C: 552 (60%) | I: 7.00 (2.97)* C: 7.00 (2.97)* | I: 17.50 (6.5)* C: 17.30 (6.4)* |
| *Notes:* ^1^ Baseline data based on modified ITT or per case (e.g., Mazlan et al., 2021), ^2^ care not provided in the ICU but participants would be eligible for ICU admission according to baseline characteristics, and ^3^ patients received treatment in the wards under advanced supportive care, because of the shortage of ICU beds in the country. Sepsis-1 definition [1], Sepsis-2 definition [2], sepsis-3 [3].  SOFA = Sequential Organ Failure Assessment score, APACHE = Acute Physiology and Chronic Health Evaluation score, PCT = Procalcitonin; SOC = standard of care, CRP = C-Reactive Protein, ICU = Intensive Care Unit.  *Median data converted to mean and standard deviation (SD).  ** RCTs included participants who met the criteria for highly suspected sepsis: SOFA score ≥2, need for antibiotics and organ support, and treatment in a critical or intensive care setting. Eligibility was confirmed through consultation with clinicians (PD and TF).  [Sepsis -1] Bone RC, Balk RA, Cerra FB, et al. Definitions for sepsis and organ failure and guidelines for the use of innovative therapies in sepsis. Chest 1992; 101: 1644-55. https://doi.org/10.1378/chest.101.6.1644  [Sepsis-2] Levy MM, Fink MP, Marshall JC, et al. 2001 sccm/esicm/accp/ats/sis international sepsis definitions conference. Intensive Care Med 2003; 29: 530-8. https://doi.org/10.1007/s00134-003-1662-x  [Sepsis-3] Singer M, Deutschman CS, Seymour CW, et al. The third international consensus definitions for sepsis and septic shock (sepsis-3). JAMA 2016; 315: 801-10. https://doi.org/10.1001/jama.2016.0287 | | | | | | | | |

| **Table S3:** Biomarker discontinuation protocols for all included studies | | | | | |
| --- | --- | --- | --- | --- | --- |
| **Author (s), year** | **Control group protocol** | **Intervention protocol** | | | |
|  |  | **Absolute thresholds** | **Relative thresholds** | **Biomarker timings (days)** | **Biomarker-guided antibiotic discontinuation protocol** |
| **PCT (intervention) vs SOC (control)** | | | | | |
| Nobre et al. (2008) [43] | Received ABX according to the local guidelines and susceptibility patterns (physicians unaware of PCT results) | Stop: < 0.25 µg/L  Strong stop: < 0.1 µg/L | 90% | Daily | **D1 or D2 PCT levels < 1 µg/L (re-evaluate D3):**  Discontinued ABX when PCT < 0.1 µg/L, provided that careful clinical evaluation ruled out severe infection.   **D1 or D2 PCT levels ≥ 1 µg/L (re-evaluate D5):**  Discontinued ABX when  1) PCT decreased by > 90% from the baseline peak value or,  2) PCT < 0.25 µg/L (stop), or 3) PCT < 0.1 µg/L (strong stop). |
| Hochreiter et al. (2009) [45] | ABX was administered over eight days in accordance with standard care (in both groups, routine laboratory analysis, including CRP and PCT count, was performed). | < 1 µg/L | 25 to 35% | Daily | Discontinued ABX when clinical signs and symptoms of infection improved and  1) PCT < 1 µg/L, or  2) PCT decreased to < 25-35% of the initial value over three consecutive days. |
| Schroeder et al. (2009) [44] | Received ABX according to clinical signs and empiric rules (in both groups, routine lab analysis, including CRP, was conducted) | < 1 µg/L | 25 to 35% | Daily | Discontinued ABX when clinical signs and symptoms of infection improved and  1) PCT < 1 µg/L, or  2) PCT decreased to < 25-35% of the initial value over three consecutive days. |
| Stolz et al. (2009) [46] | Discontinued ABX following standard clinical guidelines for ventilator-associated pneumonia (VAP) without PCT guidance. | Stop: 0.25 to 0.5 µg/L  Strong stop: < 0.25 µg/L | 80% | Daily | At D2, discontinued ABX when  1) PCT < 0.25 μg/L (strong stop) or 2) PCT between 0.25 - 0.5 µg/L, or PCT decreased by ≥ 80% compared with D0, indicating that bacterial infection was unlikely (stop).   After D2, the evaluation of PCT levels was performed by comparing daily PCT levels with those of the preceding values. |
| Deliberato et al. (2013) [47] | Received ABX based on the source of the infection and local susceptibility profile. No PCT guidance was used (CRP levels were recorded for all patients). | < 0.5 µg/L | 90% | D0, 5 and 7 | Discontinued ABX when  1) PCT decreased by > 90% from the peak level, or 2) PCT < 0.5 µg/L. |
| Liu et al. (2013) [48] | Received ABX according to standard care | < 0.25 µg/L | 90% | Daily | Discontinued ABX when no active infection, APACHE II decreased and when  1) PCT decreased > 90%, or  2) PCT < 0.25 ug/L. |
| Shehabi et al. (2014) [49] | Received ABX according to standard care (prescription according to Australian guidelines), clinicians were blinded to PCT levels. | 0.10 µg/L 0.10 to 0.25 µg/L | 90% | Daily | Discontinued ABX when  1) PCT < 0.10 µg/L, or 2) PCT between 0.10–0.25 µg/L and infection is highly unlikely, or 3) PCT reduced > 90% from baseline (and assessed ABX appropriateness and/or adequacy of source control if PCT level at 48 hours is > 70% of baseline value). |
| Bloos et al. (2016) [50] | Received ABX according to the guidelines of the German Sepsis Society (no PCT measurements were obtained until day 14) | ≤ 1 µg/L | 50% | D0, 1, 4, 7, 10, 14 | At D4, no change in ABX was recommended if the PCT level dropped by at least 50% compared with the baseline value. Otherwise, changing or optimising ABX, or introducing new interventions for source control, is recommended.  Other days, discontinued ABX when  1) PCT level ≤ 1 µg/L, or  2) PCT level drops by at least 50% compared with the previous value.  Otherwise, changes or optimisations to ABX or new interventions for source control are recommended. |
| de Jong et al. (2016) [51] | Received ABX according to the local or national guidelines. PCT levels were not measured (in both groups, CRP was measured) | ≤ 0.5 µg/L | 80% | Daily | Discontinued ABX when  1) PCT has decreased by > 80% of its peak value, or 2) PCT ≤ 0.5 μg/L. |
| Liu et al. (2017) [59] | Received ABX according to standard care | < 0.25 µg/L | 90% | Daily | Discontinued ABX when  1) PCT level decreased to 90% of the peak value or  2) PCT < 0.25μg/L. |
| Mahmutaj et al. (2017) [52] | ABX was discontinued based on the normalisation of leukocyte values and patient clinical outcome | < 0.5 µg/L | 80% | D1, 4, 7 | Discontinued ABX when 1) PCT decreased > 80% of the initial value, or 2) PCT < 0.5 µg/L. |
| Xu et al. (2017) [53] | Received ABX according to standard care | < 0.25 µg/L | 90% | Daily | Discontinued ABX when 1) PCT decreased by > 90% of the peak value, or 2) PCT < 0.25 µg/L. |
| Jeon et al. (2019) [54] | ABX was discontinued based on the clinician's guidance | ≤ 0.5 µg/L | 80% | Alternative days | Discontinued ABX when  1) PCT decreased by > 80% of its peak value, or  2) PCT ≤ 0.5 μg/L. |
| Mazlan et al. (2021) [55] | Received standard conventional 14-day ABX therapy. PCT levels not measured (CRP measured according to the physician's decision) | Stop: 0.25 to 0.5 µg/L  Strong stop: < 0.25 µg/L | 80% | D1, 3, 7, 9 | Discontinued ABX when  1) PCT < 0.25 µg/L (strong stop), or 2) PCT level decreased by > 80% from the peak PCT level, or PCT is > 0.25 but < 0.5 µg/L (stop). |
| Kyriazopoulou et al. (2021) [57] | ABX was received according to international guidelines, and the investigators were unaware of PCT levels | < 0.5 µg/L | 80% | D0, 5 | Discontinued ABX when  1) PCT was reduced by at least 80% or,  2) PCT < 0.5 μg/L. |
| Vishalashi et al. (2021) [56] | Received ABX according to the institutional protocol (routine lab analysis, and serum PCT measurements completed for both groups) | < 0.1 µg/L | 80% | Daily | Discontinued ABX when 1) PCT < 0.10 µg/L, or 2) PCT decreased by > 80% from baseline. |
| Shukeri et al. (2022) [15] | Received ABX according to national guidelines for infection types and clinical development. PCT was not measured. | Relative threshold:  0.25 to 0.5 µg/L Absolute threshold:  0.25 µg/L | 80% | Alternative days | Discontinued ABX when  1) PCT concentration has decreased by > 80% of its peak value, or 2) PCT between 0.25–0.5 µg/L (relative discontinuation threshold), or  3) PCT < 0.25 µg/L (absolute discontinuation threshold). |
| Nazer et al. (2024) [58] | Received ABX according to clinical judgement, patient clinical outcome, and microbiological cultures. | < 0.1 µg/L | 90% | Daily | Discontinued ABX when  1) PCT < 0.1 µg/L and low likelihood of infection, or  2) PCT ≥ 0.1 µg/L and < 0.25 µg/L, and low likelihood of infection (consider stopping antibiotics if the level does not increase), or  2) consider stopping or de-escalating once PCT is < 90% of the first PCT level. |
| Dark et al. (2025) [14] | Received ABX according to the daily standardised written advice supporting usual care for sepsis and antibiotic stewardship. PCT levels were not measured. | Stop: 0.25 to 0.5 µg/L  Strong stop: 0.25 µg/L | 80% | Daily | Discontinued ABX when  1) PCT < 0.25 µg/L (strong stop), or  2) PCT fall by >80% from baseline or 0.25µg/L < PCT < 0.50µg/L (stop). |
| **CRP (intervention) vs SOC (control)** | | | | | |
| Borges et al. (2020) [60] | Discontinued ABX according to clinical improvement, microbiological results, and stipulated time according to infection. CRP levels not provided to the clinical team. | < 35 mg/L | 50% | Daily | **D0 CRP levels > 100mg/L (reassessment on D5):** CRP re-evaluated after five days of ABX. Discontinued when:  1) CRP falls by more than 50% of the highest value, and no signs of active infection and SOFA decreasing, or  2) after 7 days of ABX.  **D0 CRP levels < 100mg/L (reassessment on D3):** 1) CRP < 35mg/L in the absence of infection and SOFA decreasing, or  2) after 7 days of ABX. |
| Dark et al. (2025) [14] | Received ABX according to the daily standardised written advice supporting usual care for sepsis and antibiotic stewardship. CRP levels were not measured. | Strong stop:  < 25mg/L | 50% | Daily | Discontinued ABX when 1) CRP < 25mg/L, or 2) CRP falls by more than 50% from baseline. |
| **PCT (intervention) vs CRP (control)** | | | | | |
| Oliveira et al. (2013) [61] | **D0 CRP levels > 100mg/L (reassessment on D5)** CRP re-evaluated after five days of ABX. Discontinue ABX when  1) CRP falls by more than 50% of the highest value, in the absence of infection and SOFA decrease, or  2) after 7 days of ABX  **D0 CRP levels < 100mg/L (reassessment on D4)** Discontinue ABX when 1) CRP < 25mg/L in the absence of infection, and SOFA decrease, or  2) after 7 days of ABX | < 0.1 µg/L | 90% | D0, daily until discontinuation (alternative days during ICU stay, then every 5 days when transferred to the ward) | **D0 PCT levels > 1.0µg/L (reassessment on D5)** Discontinued ABX when 1) PCT decreased by > 90% of the highest value, and SOFA decreased and no signs of active infection, or 2) after 7 days of antibiotic treatment.   **D0 PCT levels < 1.0 µg/L (reassessment on D4)** Discontinued ABX when 1) PCT < 0.1 µg/L in the absence of active infection, and SOFA decrease, or  2) after 7 days of ABX. |
| Dark et al. (2025) [14] | Discontinued ABX when 1) CRP < 25mg/L 2) CRP falls by more than 50% from baseline | Stop: 0.25 to 0.5 µg/L  Strong stop: 0.25 µg/L | 80% | Daily | Discontinued ABX when 1) PCT < 0.25 µg/L (strong stop), or  2) PCT decreased by >80% from baseline or 0.25µg/L < PCT < 0.50µg/L (stop). |
| *Notes*: ABX = antibiotics, D0 = baseline, PCT = Procalcitonin; SOC = standard of care, CRP = C-Reactive Protein | | | | | |

| **Table S4:** Summary of the overall risk of bias (RoB2) for each outcome: procalcitonin vs. standard care | | | |
| --- | --- | --- | --- |
| **Outcomes** | **No. of RCTs**  **Low** | **No. of RCTs**  **Some concerns** | **No. of RCTs**  **High** |
| Antibiotic duration | 1 | 3 | 15 |
| Mortality | 1 | 14 | 3 |
| Infection recurrence | 1 | 1 | 10 |
| Secondary infections or superinfections | 1 | 3 | 3 |
| Duration of intensive care stay | 1 | 2 | 12 |
| Duration of hospital stay | 1 | 2 | 10 |
| *Notes*: RCTs = randomised controlled trials | | | |

**Publication bias – Eggers Test**

| **Table S5:** Egger’s test results for the primary outcomes: procalcitonin vs. standard care | | | | |
| --- | --- | --- | --- | --- |
| **Outcomes** | **No. of RCTs** | **Eggers test intercept (SE)** | ***t* (df)** | ***p*** |
| Antibiotic duration | 19 | -0.81 (0.86) | -0.94 (17) | 0.359 |
| Mortality (short-term) | 18 | -0.08 (0.44) | -0.19 (16) | 0.851 |
| *Notes*: df = degrees of freedom, RCT = randomised controlled trials | | | | |

Pre-planned sensitivity, subgroup, and meta-regression analyses were conducted only for primary outcomes, such as antibiotic duration and mortality, as specified in the protocol. Also, these analyses were only conducted in studies comparing PCT-guided antibiotic discontinuation protocols with standard care, as the other comparisons had too few studies to conduct a feasible analysis.

***Sensitivity analyses***

| **Table S6:** Sensitivity analyses: procalcitonin vs. standard care | | | | |
| --- | --- | --- | --- | --- |
| **Sensitivity analyses** | **No. of RCTs** | **MD (95% CI), days** | **95% PI** | **I² (%)** |
| **Antibiotic duration, main analysis: k = 19, MD = -1.97 days (-2.56 to -1.37), I^2^ = 88.8%** | | | | |
| Excluding outcomes measuring the initial sepsis episode | 15 | -2.03 (-2.67 to -1.39) | -4.15 to 0.09 | 90.2 |
| Excluding the studies with converted data (median to mean) | 9 | -1.77 (-2.24 to -1.31) | -3.04 to -0.50 | 91.9 |
| **Mortality (short-term), main analysis: k = 18, RR = 0.95 (0.83 to 1.07), I^2^ = 20.7%** | | | | |
|  | **No. of RCTs** | **RR (95% CI)** | **95% PI** | **I² (%)** |
| Including only outcomes measuring all-cause mortality | 12 | 0.90 (0.79 to 1.03), *p* = 0.118 | 0.74 to 1.10 | 17.1 |
| Including only outcomes measuring in-hospital mortality | 9 | 0.98 (0.74 to 1.30), *p* = 0.872 | 0.61 to 1.56 | 29.6 |
| Including only outcomes measuring intensive care mortality | 5 | 1.17 (0.59 to 2.33), *p* = 0.553 | 0.38 to 3.64 | 26.3 |
| *Notes:* MD = mean difference, RR = Risk Ratios, CI = confidence intervals, PI = prediction intervals, k = number of studies, RCT = randomised controlled trials. | | | | |

***Subgroup and meta-regression analyses***

We did not perform pre-planned subgroup analyses to explore subgroup effects for adherence (k = 9), place of acquired sepsis (k = 2), or sepsis infection site (k = 2), as none of the planned analyses included more than 10 studies. Subgroup analyses were only conducted for risk of bias and frequency of PCT measurements.

| **Table S7:**  Subgroup analyses: procalcitonin vs. standard care | | | | |
| --- | --- | --- | --- | --- |
| **Subgroups** | **No. of RCTs** | **MD (95% CI), days** | **I² (%)** | **Test for subgroup differences** |
| **Antibiotic duration, main analysis: k = 19, MD = -1.97 days (-2.56 to -1.37), I^2^ = 88.8%** | | | | |
| ***Risk of bias*** | | | | |
| Low/some | 4 | -2.51 (-5.56 to 0.55) | 92.7 | *Q*(1) = 0.58, *p* = 0.445 |
| High | 15 | -1.75 (-2.25 to -1.26) | 88.1 |  |
| ***Biomarker measurement timings*** | | | | |
| Daily | 12 | -1.78 (-2.20 to -1.35) | 89.3 | *Q*(1) = 0.69, *p* = 0.406 |
| Non-Daily | 7 | -2.37 (-4.05 to -0.69) | 89.6 |  |
| **Mortality (short-term), main analysis: k = 18, RR = 0.95 (0.83 to 1.07), I^2^ = 20.7%** | | | | |
|  | **No. of RCTs** | **RR (95% CI)** | **I² (%)** | **Test for subgroup differences** |
| ***Risk of bias*** | | | | |
| Low/some | 15 | 0.96 (0.83 to 1.11) | 32.1 | *Q*(1) = 2.57, *p* = 0.109 |
| High | 3 | 0.76 (0.45 to 1.29) | 0.0 |  |
| ***Biomarker measurement timings*** | | | | |
| Daily | 12 | 0.98 (0.85 to 1.13) | 17.9 | Q(1) = 0.92, *p* = 0.336 |
| Non-Daily | 6 | 0.84 (0.58 to 1.22) | 27.6 |  |
| *Notes:* MD = mean difference, RR = Risk Ratios, CI = confidence intervals, CI = confidence intervals, RCTs = randomised controlled trials. | | | | |

We conducted subgroup analyses stratified by thresholds twice. First, we included all studies (Table S5). We then excluded Nazer et al. because their study reported a mortality risk of 45%, which was substantially higher than the average of 21% across the other included studies. When we removed this study, the results for the duration of antibiotic therapy were similar. For mortality rates, we found no significant differences between PCT and standard care in the first subgroup (PCT ≤ 0.25 or >80–90% decrease) *after* excluding the Nazer study. The impact of this study might be due to the criteria for antibiotic discontinuation (PCT < 0.1 µg/L) or because all participants had cancer and sepsis.

| **Table S8:** Subgroup analysis of discontinuation protocols: procalcitonin vs. standard care | | | | |
| --- | --- | --- | --- | --- |
| **Subgroups** | **No. of RCTs** | **MD (95% CI), days** | **I² (%)** | **Test for subgroup differences** |
| **Antibiotic duration, main analysis: k = 19, MD = -1.97 days (-2.56 to -1.37), I^2^ = 88.8%** | | | | |
| Studies with absolute thresholds ≤ 0.25 or relative thresholds at 80-90% decrease | 8 | -1.90 (-2.44 to -1.35) | 92.4 | *Q*(2) = 2.27,  *p* = 0.321 |
| Studies with absolute thresholds between 0.25 and 0.50 or relative thresholds at 80% decrease | 8 | -2.58 (-4.03 to -1.12) | 85.6 |  |
| Studies with absolute thresholds > 0.50, or relative thresholds ≤ 50% decrease | 3 | -1.26 (-3.92 to 1.39) | 89.2 |  |
| **Mortality (short-term), main analysis: k = 18, RR = 0.95 (0.83 to 1.07), I^2^ = 20.7%** | | | | |
|  | **No. of RCTs** | **RR (95% CI)** | **I^2^ (%)** | **Test for subgroup differences** |
| Studies with absolute thresholds ≤ 0.25 or relative thresholds at 80-90% decrease | 8 | 1.24 (1.01 to 1.51) | 0.0 | *Q*(2) = 13.10,  *p* = 0.001 |
| Studies with absolute thresholds between 0.25 and 0.50 or relative thresholds at 80% decrease | 7 | 0.84 (0.66 to 1.06) | 45.6 |  |
| Studies with absolute thresholds > 0.50, or relative thresholds ≤ 50% decrease | 3 | 0.92 (0.85 to 0.99) | 0 |  |
| *Notes:* MD = mean difference, RR = risk ratios, CI = confidence intervals, randomised controlled trials | | | | |

| **Table S9:** Subgroup analysis of discontinuation protocols: procalcitonin vs. standard care (excluding Nazer et al.) | | | | |
| --- | --- | --- | --- | --- |
| **Subgroups** | **No. of RCTs** | **MD (95% CI), days** | **I² (%)** | **Test for subgroup differences** |
| **Antibiotic duration, main analysis: k = 18, MD = -2.02 days (-2.64 to -1.40), I^2^ = 89.4%** | | | | |
| Studies with absolute thresholds ≤ 0.25 or relative thresholds at 80-90% decrease | 7 | -1.96 (-2.55 to -1.38) | 93.5 | *Q(*2) = 2.28,  *p* = 0.321 |
| Studies with absolute thresholds between 0.25 and 0.50 or relative thresholds at 80% decrease | 8 | -2.58 (-4.03 to -1.12) | 85.6 |  |
| Studies with absolute thresholds > 0.50, or relative thresholds ≤ 50% decrease | 3 | -1.26 (-3.92 to 1.39) | 89.2 |  |
| **Mortality (short-term), main analysis: k = 17, RR = 0.91 (0.82 to 1.01), I^2^ = 0.00%** | | | | |
|  | **No. of RCTs** | **RR (95% CI)** | **I^2^ (%)** | **Test for subgroup differences** |
| Studies with absolute thresholds ≤ 0.25 or relative thresholds at 80-90% decrease | 7 | 1.11 (0.88 to 1.41) | 0.0 | *Q*(2) = 4.83,  *p* = 0.090 |
| Studies with absolute thresholds between 0.25 and 0.50 or relative thresholds at 80% decrease | 7 | 0.84 (0.66 to 1.06) | 45.6 |  |
| Studies with absolute thresholds > 0.50, or relative thresholds ≤ 50% decrease | 3 | 0.92 (0.85 to 0.99) | 0.0 |  |
| *Notes:* MD = mean difference, RR = Risk Ratios, CI = confidence intervals, CI = confidence intervals, RCTs = randomised controlled trials | | | | |

| **Table S10:** Univariable meta-regression of baseline SOFA and APACHE scores as moderators of antibiotic duration and mortality | | | | | | | | |
| --- | --- | --- | --- | --- | --- | --- | --- | --- |
| **Moderators** | | **No. of studies** | **R^2^** | ***Coefficient*** | **Standard error** | ***t*** | ***p*** | **95% CI** |
| **Antibiotic duration** | | | | | | | | |
| Baseline SOFA scores | Intercept | 14 |  | -5.65 | 1.20 | -4.70 | 0.0005 | -8.27 to -3.03 |
|  | Moderator | 14 | 70.19 | 0.55 | 0.17 | 3.22 | 0.007 | 0.18 to 0.92 |
|  | Test for moderators | *F = 10.38, p = 0.007* | | | | | | |
| Baseline APACHE scores | Intercept | 12 |  | -2.15 | 0.74 | -2.92 | 0.015 | -3.78 to -0.51 |
|  | Moderator | 12 | 0.00 | 0.01 | 0.02 | 0.52 | 0.612 | -0.04 to 0.07 |
|  | Test for moderators | *F = 0.27, p = 0.612* | | | | | | |
| **Mortality (short-term)** | | | | | | | | |
| Baseline SOFA scores | Intercept | 14 |  | -0.289 | 0.354 | -0.82 | 0.430 | -1.06 to 0.48 |
|  | Moderator | 14 | 0.00 | 0.032 | 0.049 | 0.64 | 0.539 | -0.07 to 0.14 |
|  | Test for moderators | *F = 0.42, p = 0.529* | | | | | | |
| Baseline APACHE scores | Intercept | 12 |  | 0.088 | 0.146 | 0.605 | 0.559 | -0.24 to 0.41 |
|  | Moderator | 12 | 6.61 | -0.004 | 0.004 | -1.04 | 0.323 | -0.01 to 0.00 |
|  | Test for moderators | *F = 1.08, p = 0.323* | | | | | | |
| *Notes*: CI = confidence intervals, SOFA = Sequential Organ Failure Assessment score, APACHE = SOFA = Sequential Organ Failure Assessment score. R^2^ = amount (%) of heterogeneity accounted for. | | | | | | | | |

| **Table S11:** C-reactive protein compared to standard care for critically ill adult patients with sepsis | | | | | |
| --- | --- | --- | --- | --- | --- |
| **Outcomes** | **No. of participants (studies)** | **Certainty of the evidence (GRADE)** | **Relative effect (95% CI)** | **Anticipated absolute effects** | |
|  |  |  |  | **Risk with standard care** | **Risk difference with CRP** |
| Antibiotic duration | 1927 (2 RCTs) | ⨁◯◯◯ Very low^a,b^ | - | The mean antibiotic duration was 10.35 days | MD **0.58 days lower** (11.04 lower to 9.89 higher) |
| Short-term mortality | 1924 (2 RCTs) | ⨁⨁◯◯ Low^b^ | RR: 1.10 (0.69 to 1.76) | 192 per 1,000 | **19 more per 1,000** (59 fewer to 146 more) |
| Infection recurrence | 1951 (2 RCTs) | ⨁⨁◯◯ Low^b^ | RR: 1.31 (0.00 to 552.21) | 6 per 1,000 | **2 more per 1,000** (6 fewer to 3,378 more) |
| Secondary infection or superinfection | 1951 (2 RCTs) | ⨁⨁◯◯ Low^b^ | RR: 1.05 (0.44 to 2.52) | 47 per 1,000 | **2 more per 1,000** (26 fewer to 71 more) |
| Length of hospital stay | 1924 (2 RCTs) | ⨁◯◯◯ Very low^a,b^ | - | The mean length of hospital stay was 21.92 days | MD **1.08 days higher** (32.01 lower to 34.16 higher) |
| Length of intensive care stay | 1663 (2 RCTs) | ⨁⨁◯◯ Low^b^ | - | The mean length of intensive care stay was 8.37 days | MD **0.10 days lower** (1.67 lower to 1.48 higher) |
| *Notes*: The risk in the intervention group (and its 95% confidence interval) is based on the assumed risk in the comparison group and the relative effect of the intervention (and its 95% CI). CI = confidence interval, MD = mean difference, RR = risk ratio.  **Explanations:**  a. Downgraded one level because of moderate heterogeneity  b. Downgraded two levels because of a small sample and wide confidence intervals | | | | | |

| **Table S12:** Procalcitonin compared with CRP for critically ill adult patients with sepsis | | | | | |
| --- | --- | --- | --- | --- | --- |
| **Outcomes** | **No. of participants (studies)** | **Certainty of the evidence (GRADE)** | **Relative effect (95% CI)** | **Anticipated absolute effects** | |
|  |  |  |  | **Risk with CRP** | **Risk difference with PCT** |
| Antibiotic duration | 1916 (2 RCTs) | ⨁◯◯◯ Very low^a,b^ | - | The mean antibiotic duration was 8.9 days | MD **0.08 days lower** (10.76 lower to 10.60 higher) |
| Short-term mortality | 1847 (2 RCTs) | ⨁⨁⨁◯ Moderate^c^ | RR: 0.99 (0.94 to 1.05) | 217 per 1,000 | **2 fewer per 1,000** (13 fewer to 11 more) |
| Infection recurrence | 1910 (2 RCTs) | ⨁⨁◯◯ Low^b^ | RR: 2.29 (0.76 to 6.92) | 6 per 1,000 | **8 more per 1,000** (2 fewer to 37 more) |
| Secondary infection or superinfection | 1816 (1 RCT) | ⨁⨁◯◯ Low^b^ | RR: 1.07 (0.64 to 1.80) | 30 per 1,000 | **2 more per 1,000** (11 fewer to 24 more) |
| Length of hospital stay | 1876 (2 RCTs) | ⨁◯◯◯ Very low^b,d^ | - | The mean length of hospital stay was 22.8 days | MD **2.0 days higher** (37.19 lower to 41.20 higher) |
| Length of intensive care stay | 1628 (2 RCTs) | ⨁⨁◯◯ Very low^b,d^ | - | The mean length of intensive care stay was 9.67 days | MD **1.11 days higher** (16.99 lower to 19.22 higher) |
| *Notes:* The risk in the intervention group (and its 95% confidence interval) is based on the assumed risk in the comparison group and the relative effect of the intervention (and its 95% CI). CI = confidence interval, MD = mean difference, RR = risk ratio.  **Explanations:**  a. Downgraded two levels because of substantial heterogeneity and inconsistent effects  b. Downgraded two levels because of wide confidence intervals and small sample size.  c. Downgraded one level because of the small sample size  d. Downgraded one level because of moderate heterogeneity | | | | | |
