# Supplementary material for: Clinical effectiveness of procalcitonin‐ or C‐reactive protein‐guided antibiotic discontinuation protocols for adult patients who are critically ill with sepsis: a rapid systematic review and meta‐analysis
Source: Anaesthesia. 2026 Jan 8;81(4):556–69. doi: 10.1111/anae.70109 (PMC12973355; doi:10.1111/anae.70109)
Supplement: Supplementary file 2 — Figure S1. MEDLINE database search strategy. Figures S2–S4. Risk of bias judgements for duration of antibiotic therapy: procalcitonin‐guided compared with standard care; CRP‐guided compared with standard care; and procalcitonin‐guided compared with CRP‐guided care. Figures S5–S7. Risk of bias judgements for mortality: procalcitonin‐guided compared with standard care; CRP‐guided compared with standard care; and procalcitonin‐guided compared with CRP‐guided care. Figures S8–S10. RoB2 judgements for infection recurrence: procalcitonin‐guided compared with standard care; CRP‐guided compared with standard care; and procalcitonin‐guided compared with CRP‐guided care. Figures S11 and S12. RoB2 judgements for secondary infections or superinfections: procalcitonin‐guided compared with standard care; and CRP‐guided compared with standard care. Figures S13–S15. RoB2 judgements for duration of hospital stay: procalcitonin‐guided compared with standard care; CRP‐guided compared to standard care; and procalcitonin‐guided compared with CRP‐guided care. Figures S16–S18. RoB2 judgements for duration of ICU stay: procalcitonin‐guided compared with standard care; CRP‐guided compared with standard care; and procalcitonin‐guided compared with CRP‐guided care. Figure S19. Studies comparing procalcitonin‐guided with standard care for the outcome of duration of antibiotic therapy. Figure S20. Duration of antibiotic therapy in patients receiving CRP‐guided compared with standard care. Figure S21. Duration of antibiotic therapy in patients receiving procalcitonin‐guided compared with CRP‐guided care. Figure S22. Mortality (long‐term) in patients receiving procalcitonin‐guided compared with standard care. Figure S23. Studies comparing procalcitonin‐guided with standard care for the outcome of mortality (short‐term). Figure S24. Mortality (short‐term) in patients receiving CRP‐guided compared with standard care. Figure S25. Mortality (short‐term) in patients receiving procalcitonin‐guided comp [file ANAE-81-556-s003.docx]

|  | Database search performed on 14/04/2025 |
| --- | --- |
| 1 | exp Sepsis/ |
| 2 | sepsis.ti,ab. |
| 3 | exp Shock, Septic/ |
| 4 | (septic adj2 shock).ti,ab. |
| 5 | septic*.ti,ab. |
| 6 | exp Systemic Inflammatory Response Syndrome/ |
| 7 | systemic inflammatory response syndrome.ti,ab. |
| 8 | SIRS.ti,ab. |
| 9 | exp Multiple Organ Failure/ |
| 10 | multiple organ failure.ti,ab. |
| 11 | bloodstream infection*.ti,ab. |
| 12 | blood stream infection*.ti,ab. |
| 13 | exp Critical Illness/ |
| 14 | critical illness*.ti,ab. |
| 15 | critically ill.ti,ab. |
| 16 | exp Critical Care/ |
| 17 | critical care.ti,ab. |
| 18 | critical care unit*.ti,ab. |
| 19 | ICU.ti,ab. |
| 20 | ITU.ti,ab. |
| 21 | CCU.ti,ab. |
| 22 | exp Intensive Care Units/ |
| 23 | intensive care unit*.ti,ab. |
| 24 | intensive therapy unit*.ti,ab. |
| 25 | 1 or 2 or 3 or 4 or 5 or 6 or 7 or 8 or 9 or 10 or 11 or 12 or 13 or 14 or 15 or 16 or 17 or 18 or 19 or 20 or 21 or 22 or 23 or 24 |
| 26 | exp Calcitonin/ |
| 27 | calcitonin.ti,ab. |
| 28 | exp Procalcitonin/ |
| 29 | procalcitonin.ti,ab. |
| 30 | PCT.ti,ab. |
| 31 | calcitonin precursor*.ti,ab. |
| 32 | exp C-Reactive Protein/ |
| 33 | C-reactive.ti,ab. |
| 34 | CRP.ti,ab. |
| 35 | protein precursor*.ti,ab. |
| 36 | biomarker*.ti,ab. |
| 37 | 26 or 27 or 28 or 29 or 30 or 31 or 32 or 33 or 34 or 35 or 36 |
| 38 | exp Anti-Bacterial Agents/ |
| 39 | anti-bacterial agent*.ti,ab. |
| 40 | exp Anti-Infective Agents/ |
| 41 | anti-infective agent*.ti,ab. |
| 42 | antibiotic*.ti,ab. |
| 43 | antimicrobial*.ti,ab. |
| 44 | 38 or 39 or 40 or 41 or 42 or 43 |
| 45 | exp Clinical Trial/ |
| 46 | clinical trial*.ti,ab. |
| 47 | randomi*ed controlled trial.ti,ab. |
| 48 | controlled clinical trial.ti,ab. |
| 49 | controlled study.ti,ab. |
| 50 | RCT.ti,ab. |
| 51 | exp Randomized Controlled Trial/ |
| 52 | randomi?ed.ti,ab. |
| 53 | random*.ti,ab. |
| 54 | trial*.ti. |
| 55 | 45 or 46 or 47 or 48 or 49 or 50 or 51 or 52 or 53 or 54 |
| 56 | exp animals/ not humans.sh. |
| 57 | 55 not 56 |
| 58 | 25 and 37 and 44 and 57 |
| 59 | Limit to 2005 to current |

**Figure S1:** Medline database search strategy

**Risk of bias (Rob2) judgements**

**
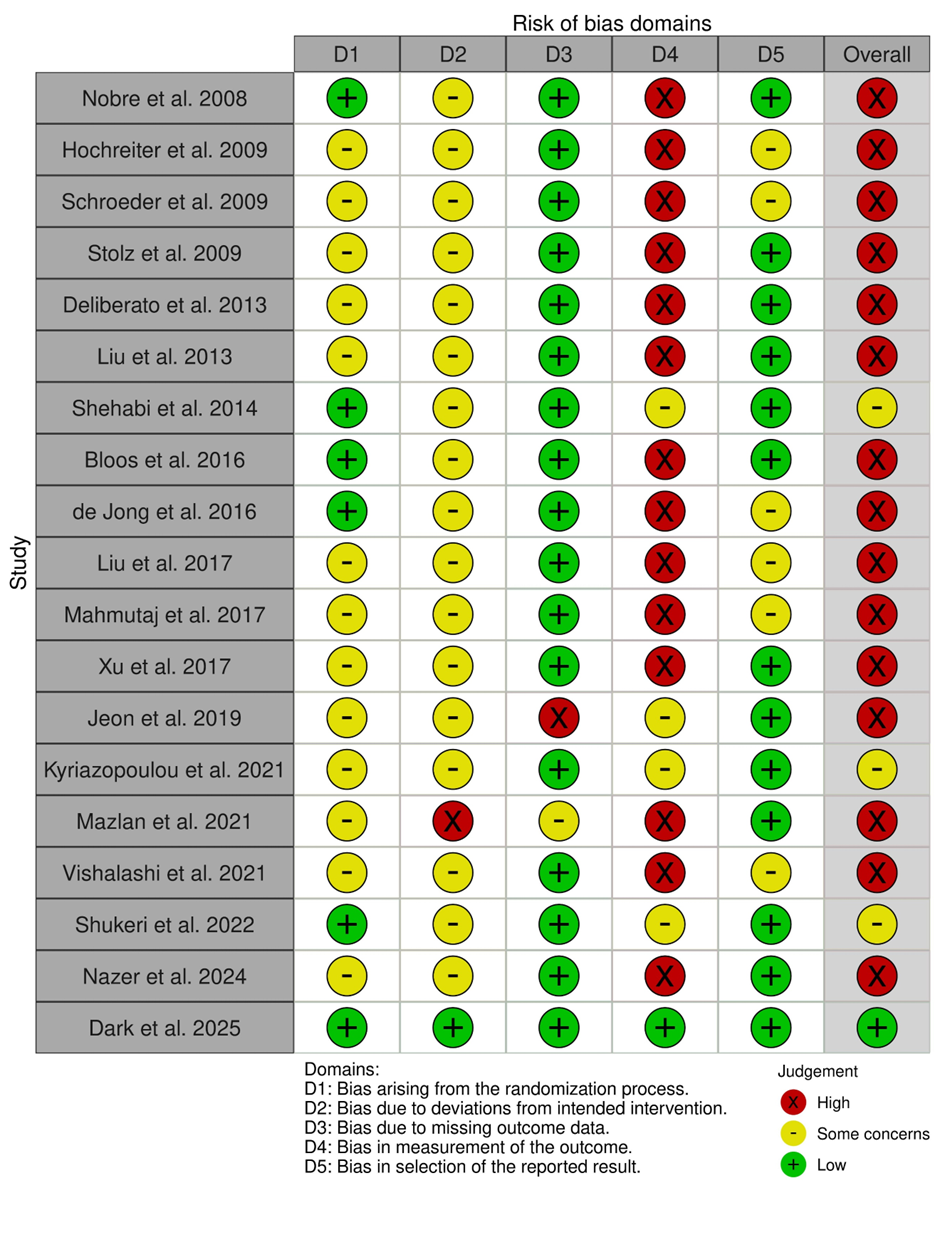
**

**Figure S2**: RoB2 judgements for antibiotic duration: Procalcitonin compared to standard care

**
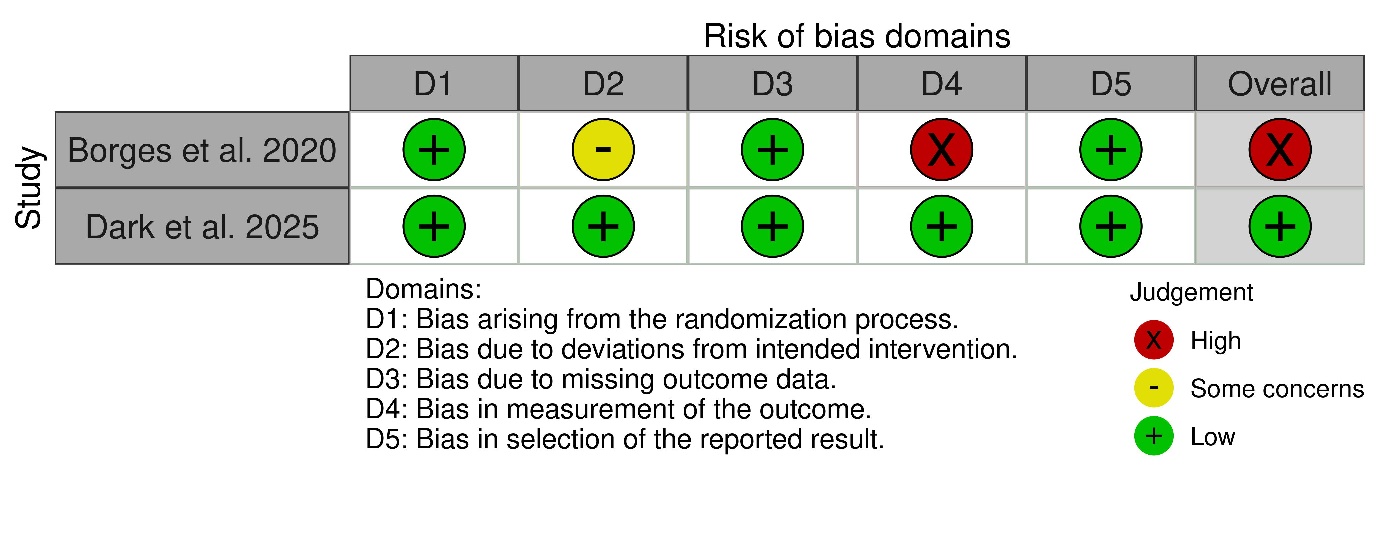
**

**Figure S3**: RoB2 judgements for antibiotic duration: C-reactive protein compared to standard care

**
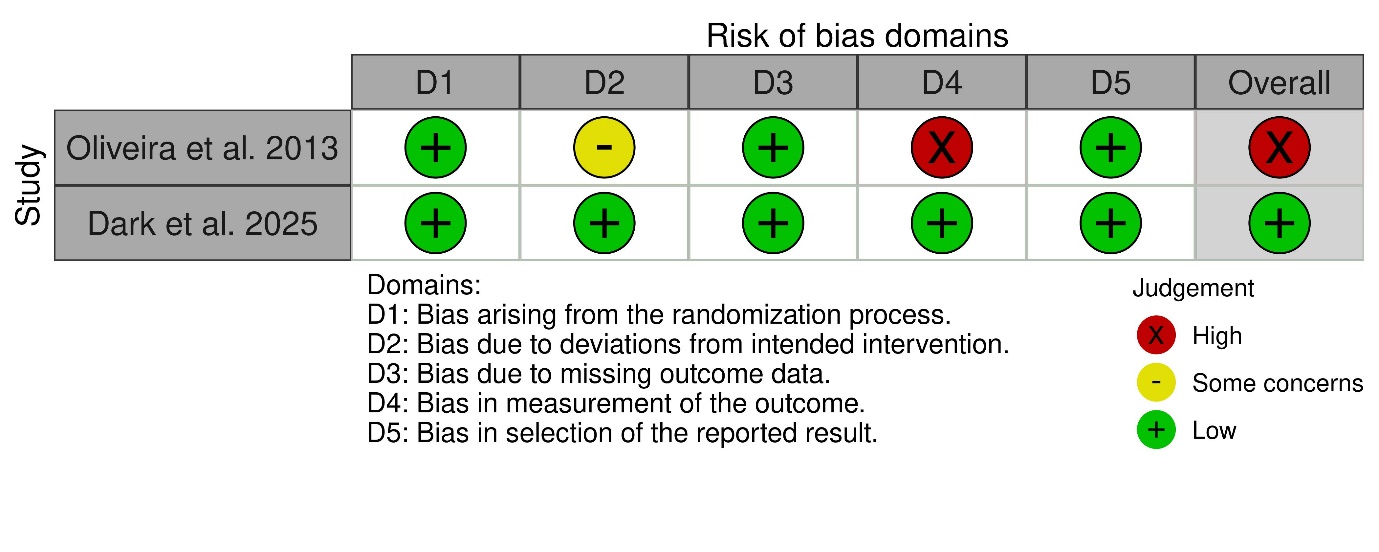
**

**Figure S4**: RoB2 judgements for antibiotic duration: Procalcitonin compared to C-reactive protein


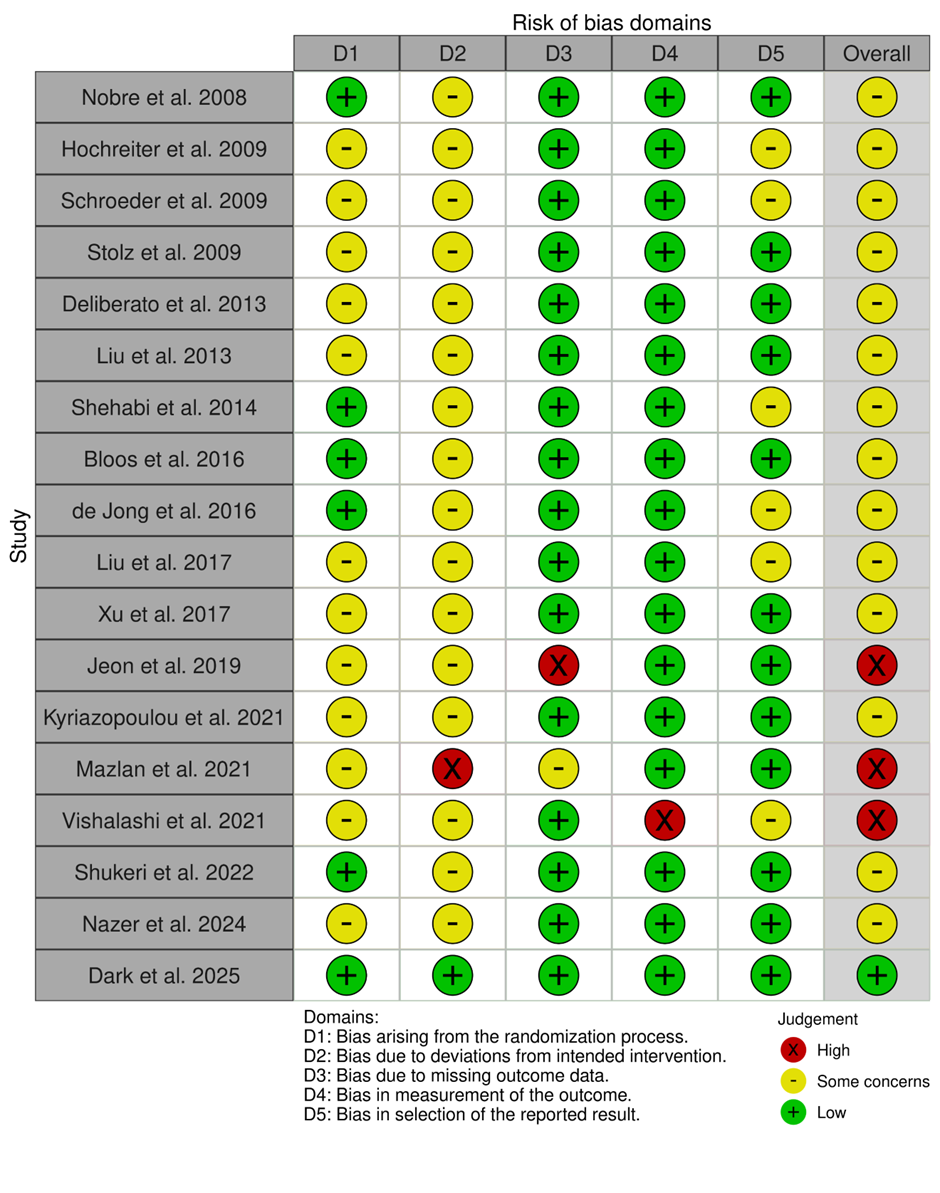


**Figure S5**: RoB2 judgements for mortality: Procalcitonin compared to standard care

**
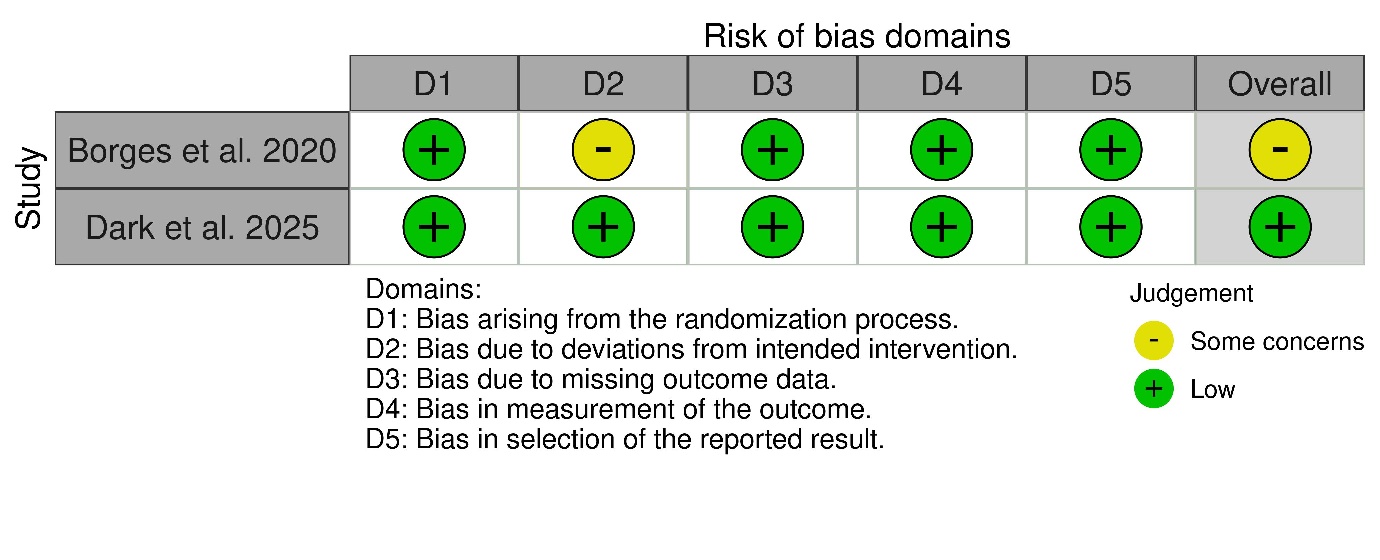
**

**Figure S6**: RoB2 judgements for mortality: C-reactive protein compared to standard care


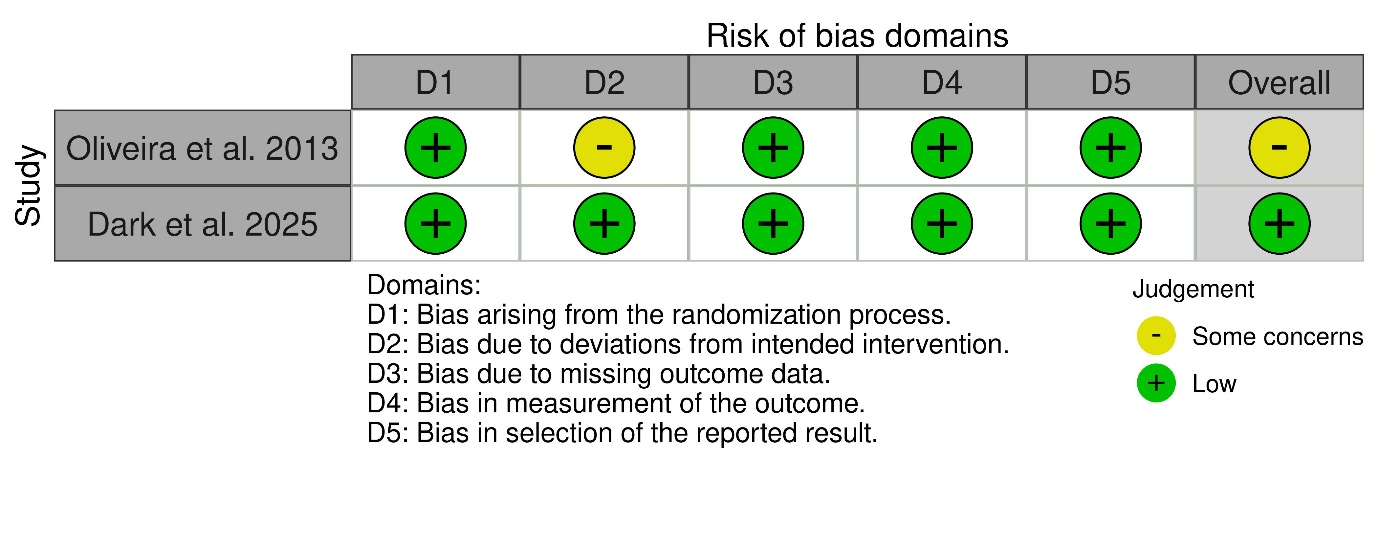


**Figure S7**: RoB2 judgements for mortality: Procalcitonin compared to C-reactive protein

**
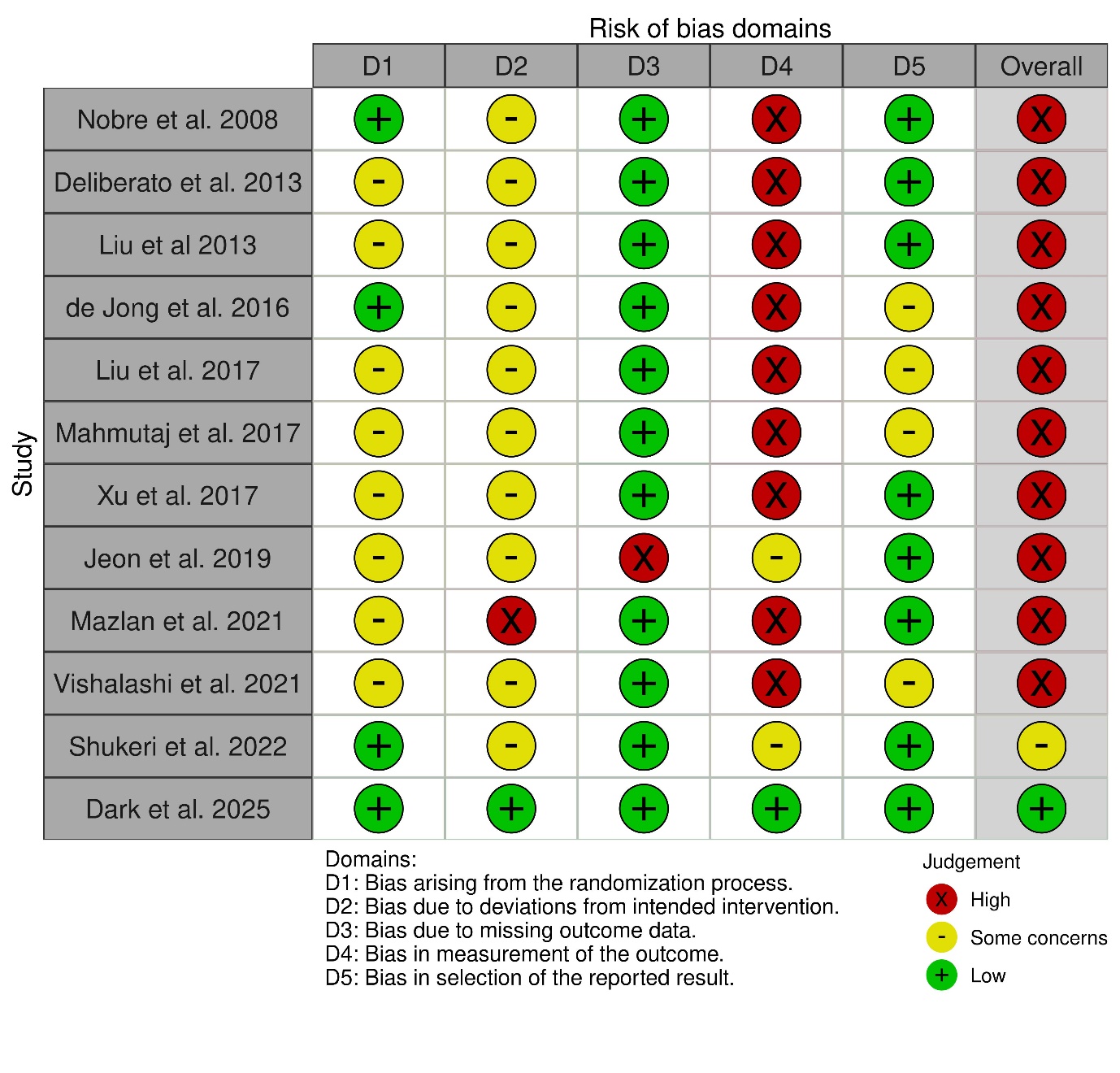
**

**Figure S8**: RoB2 judgements for infection recurrence: Procalcitonin compared to standard care

**
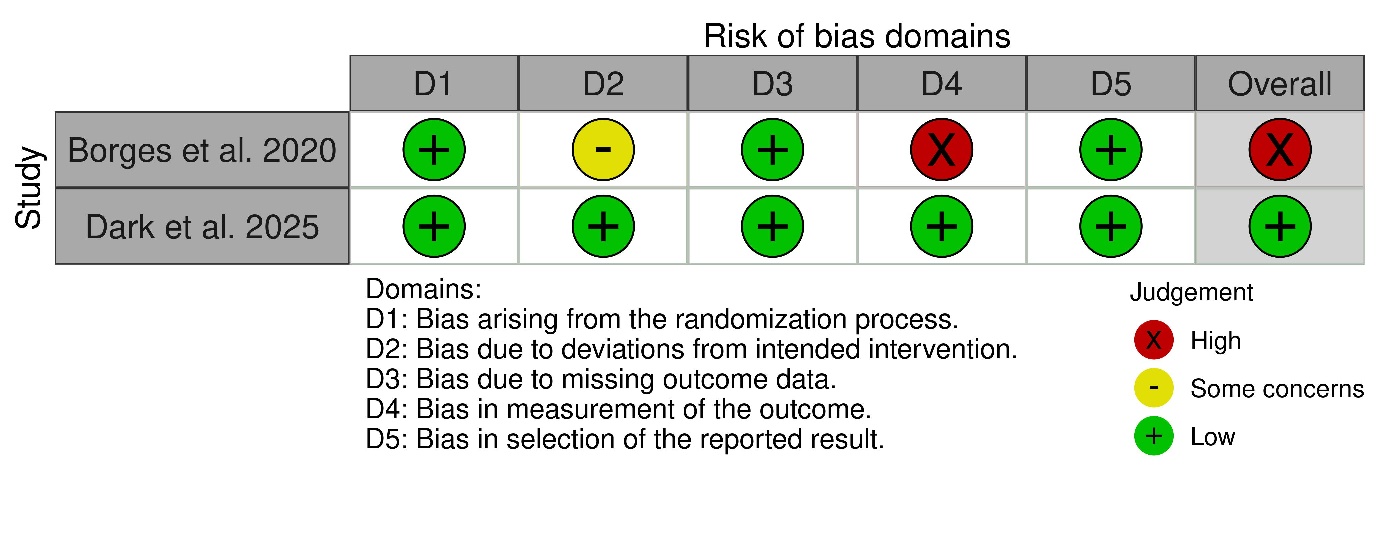
**

**Figure S9**: RoB2 judgements for infection recurrence: C-reactive protein compared to standard care

**
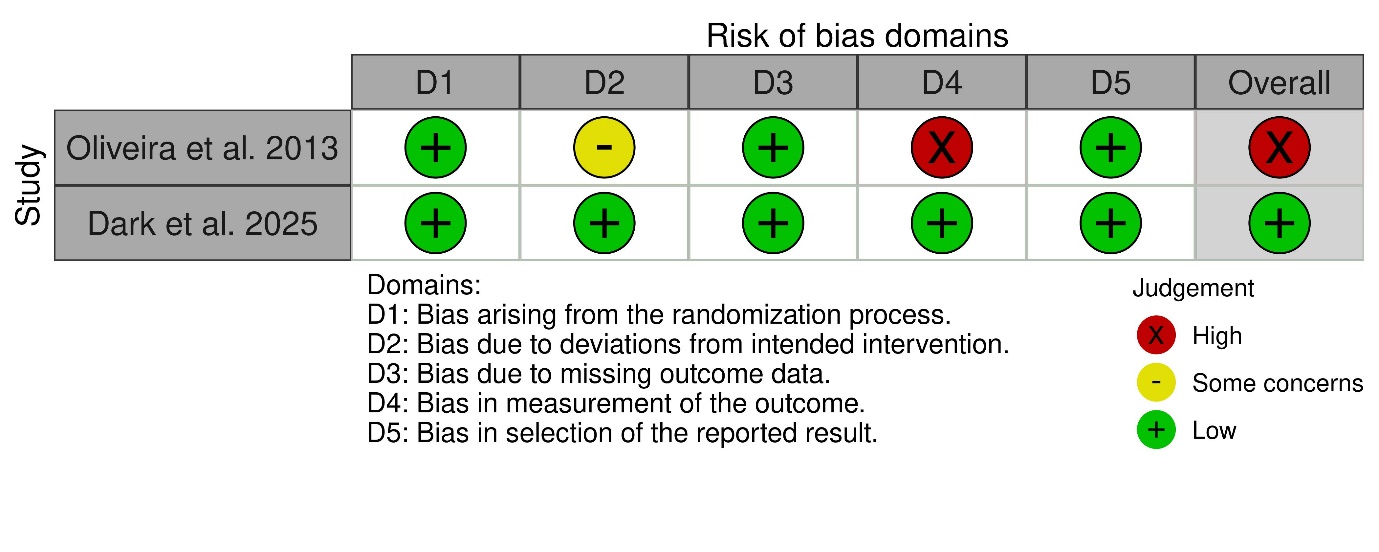
**

**Figure S10**: RoB2 judgements for infection recurrence: Procalcitonin compared to C-reactive protein


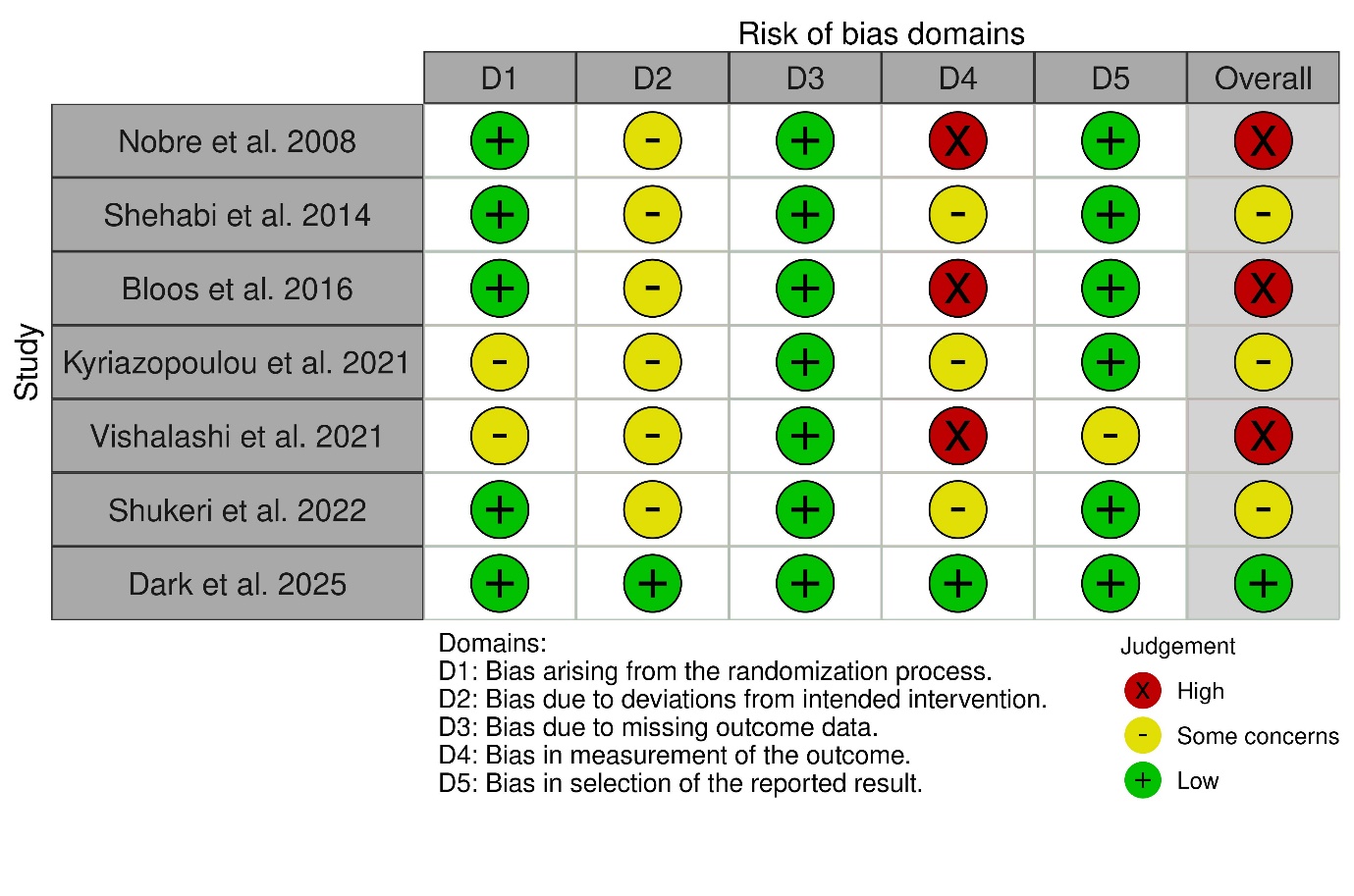


**Figure S11**: RoB2 judgements for secondary infections or superinfections: Procalcitonin compared to standard care


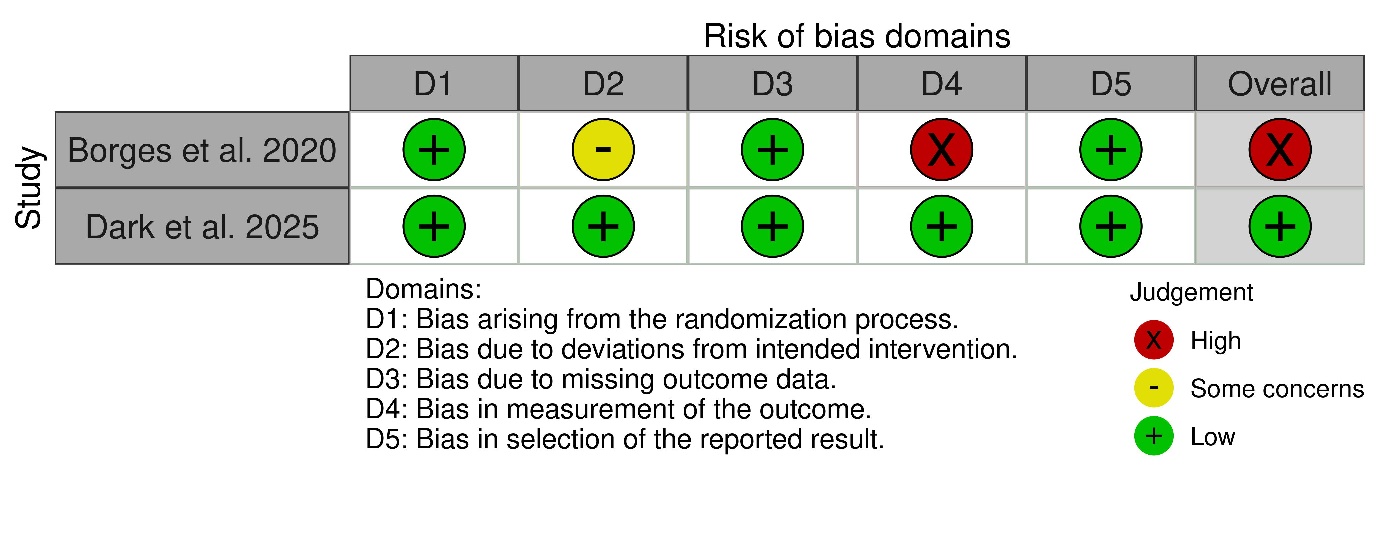
**Figure S12**: RoB2 judgements for secondary infections or superinfections: C-reactive protein compared to standard care


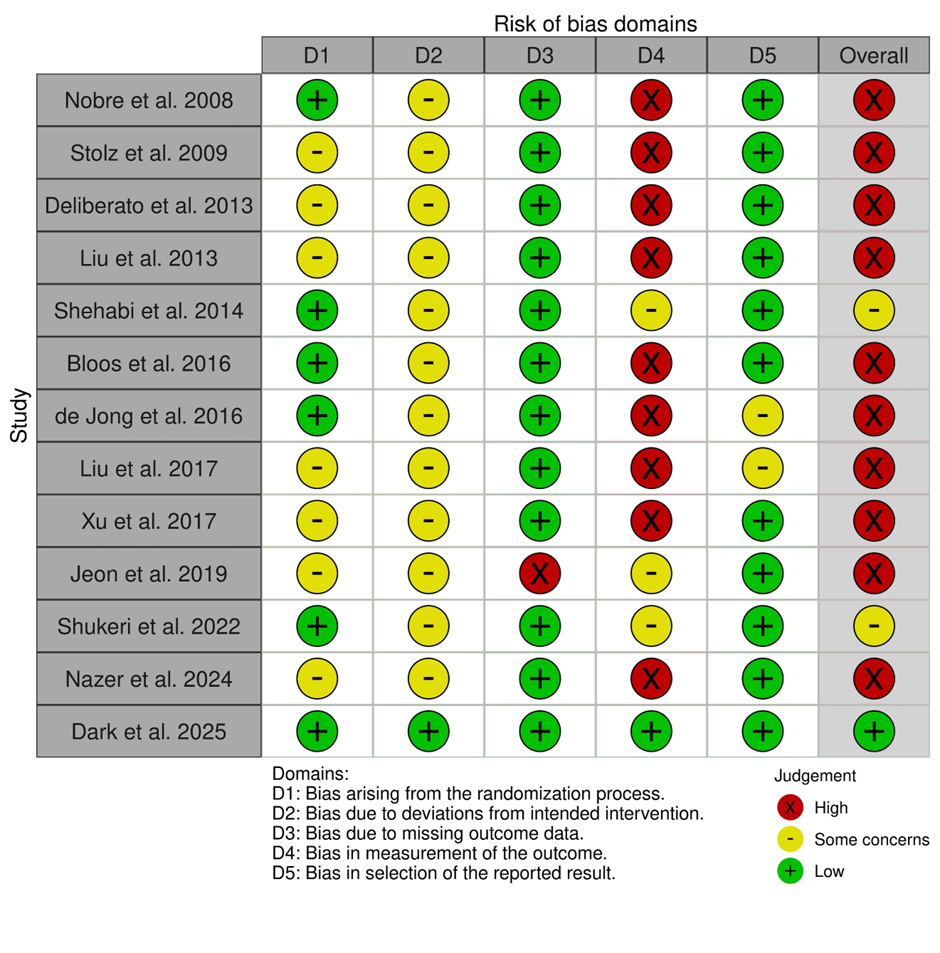


**Figure S13**: RoB2 judgements for duration of hospital stay – Procalcitonin compared to standard care


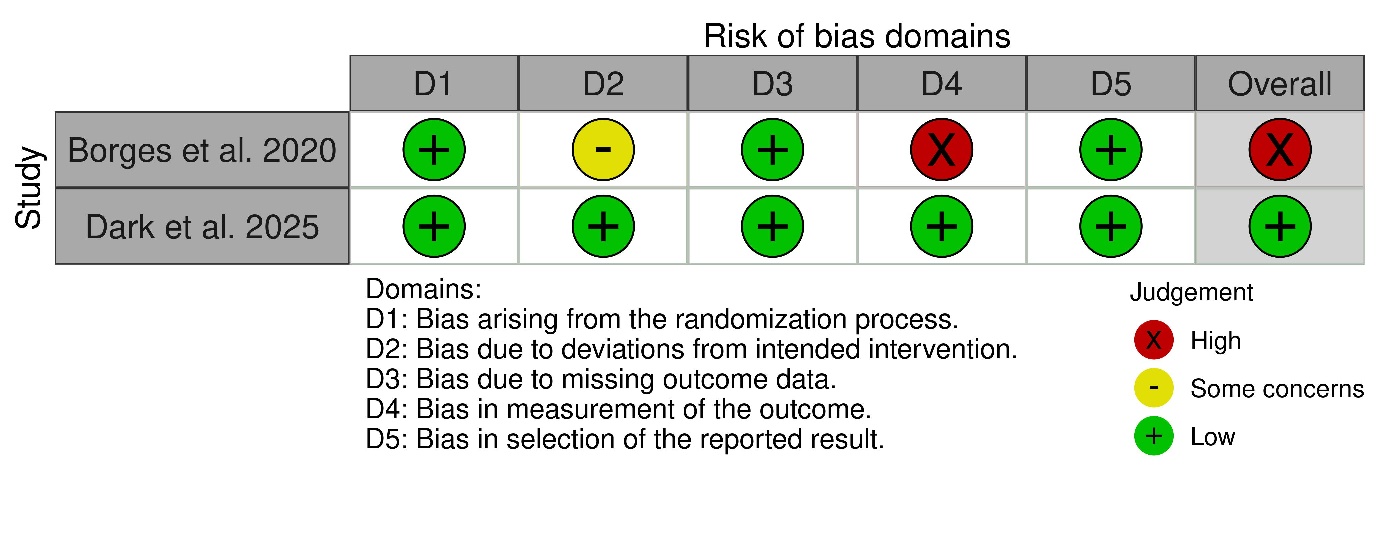


**Figure S14**: RoB2 judgements for duration of hospital stay: C-reactive protein compared to standard care


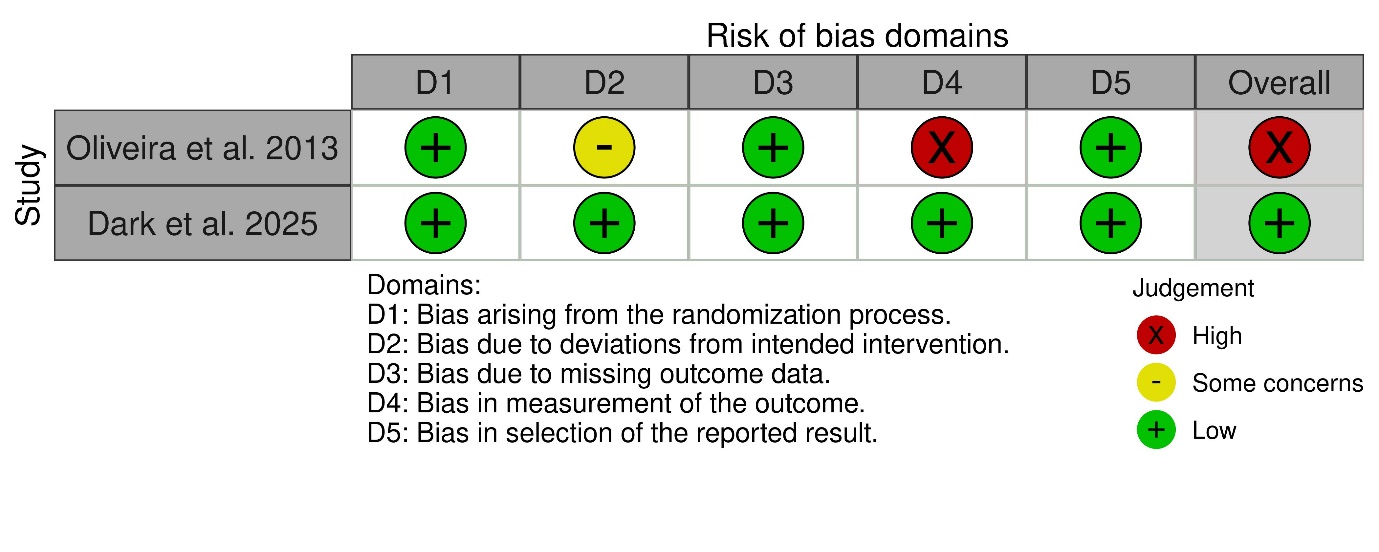


**Figure S15**: RoB2 judgements for duration of hospital stay: Procalcitonin compared to C-reactive protein

**
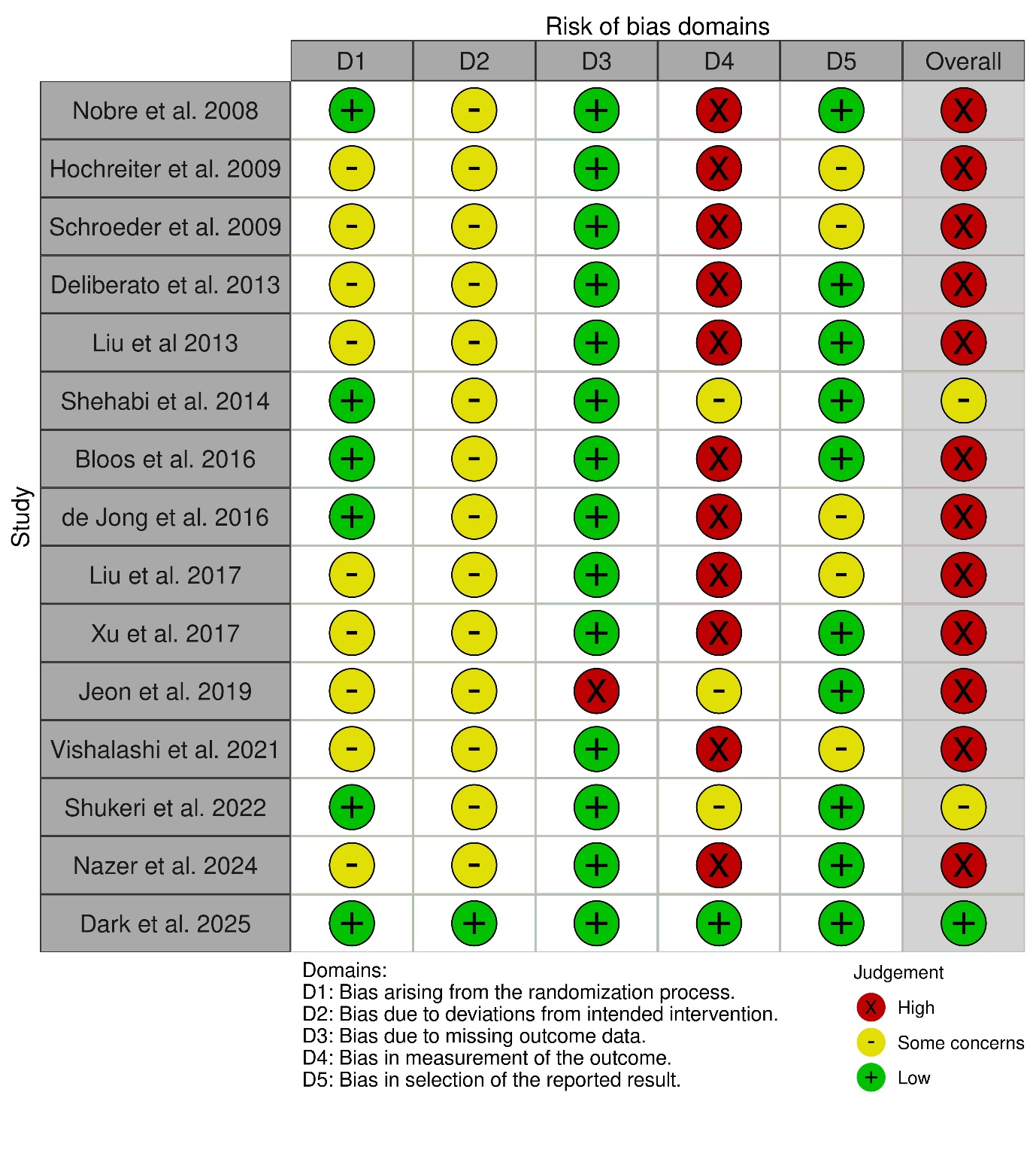
**

**Figure S16**: RoB2 judgements for duration of intensive care stay: Procalcitonin compared to standard care


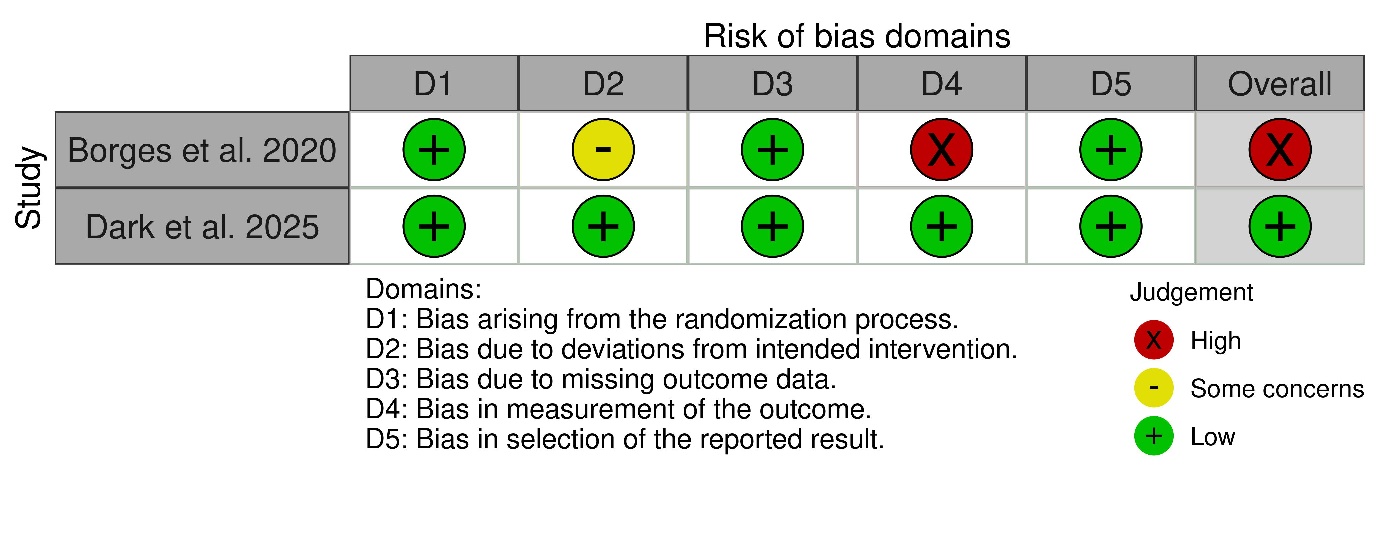


**Figure S17**: RoB2 judgements for duration of intensive care stay: C-reactive protein compared to standard care


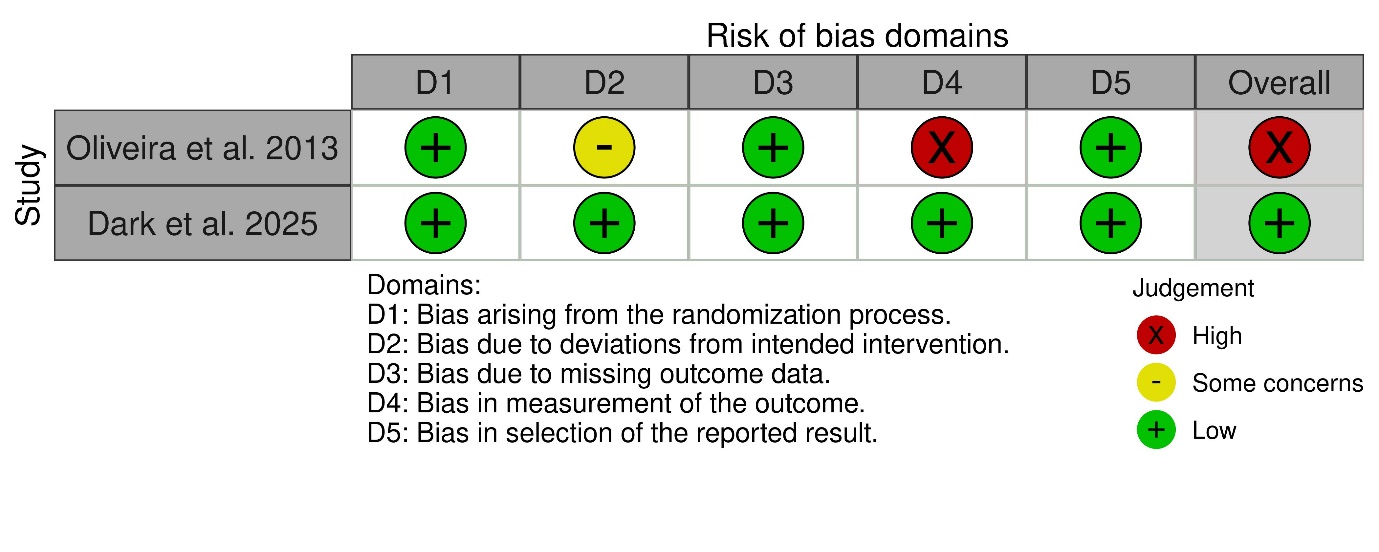


**Figure S18**: RoB2 judgements for duration of intensive care stay: Procalcitonin compared to C-reactive protein

**Publication bias – Funnel plots**


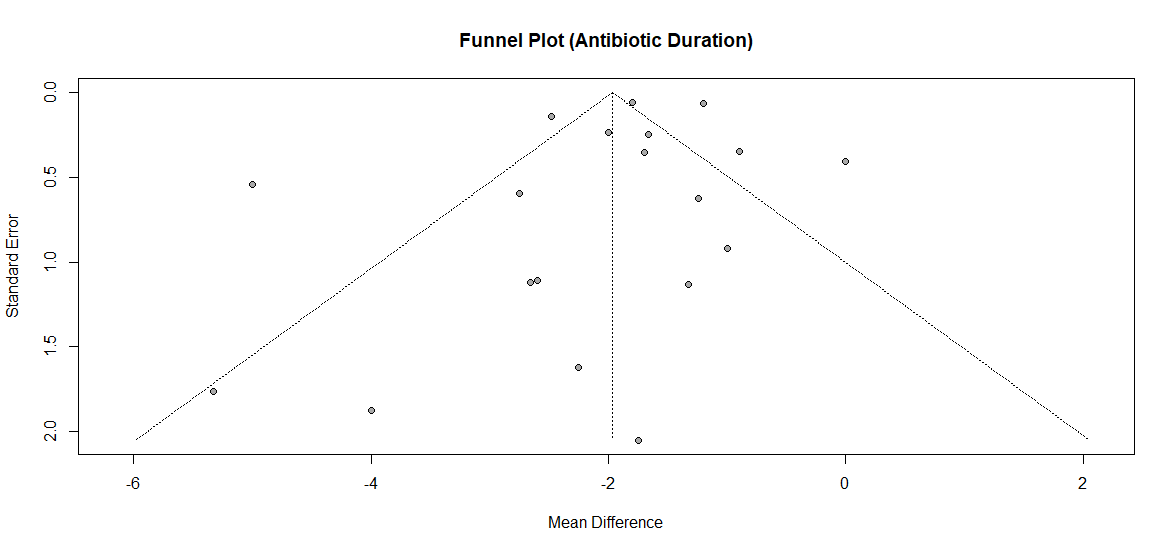


**Figure S19**: Studies comparing procalcitonin to standard care for the outcome of antibiotic duration.

**Forest plots**


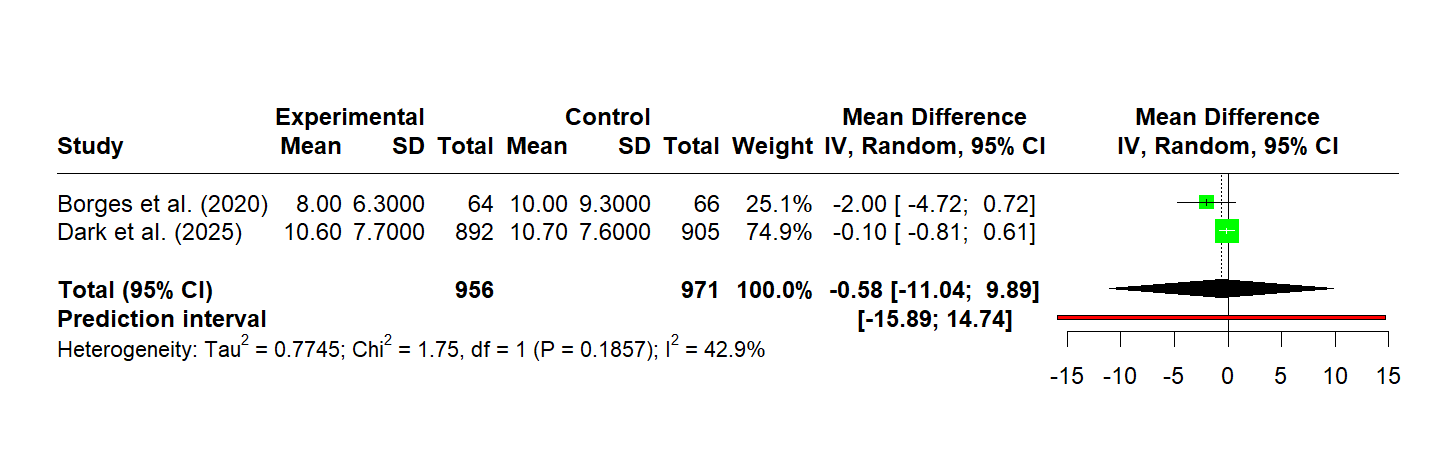


**Figure S20**: Antibiotic duration in participants receiving C-reactive protein compared to standard care


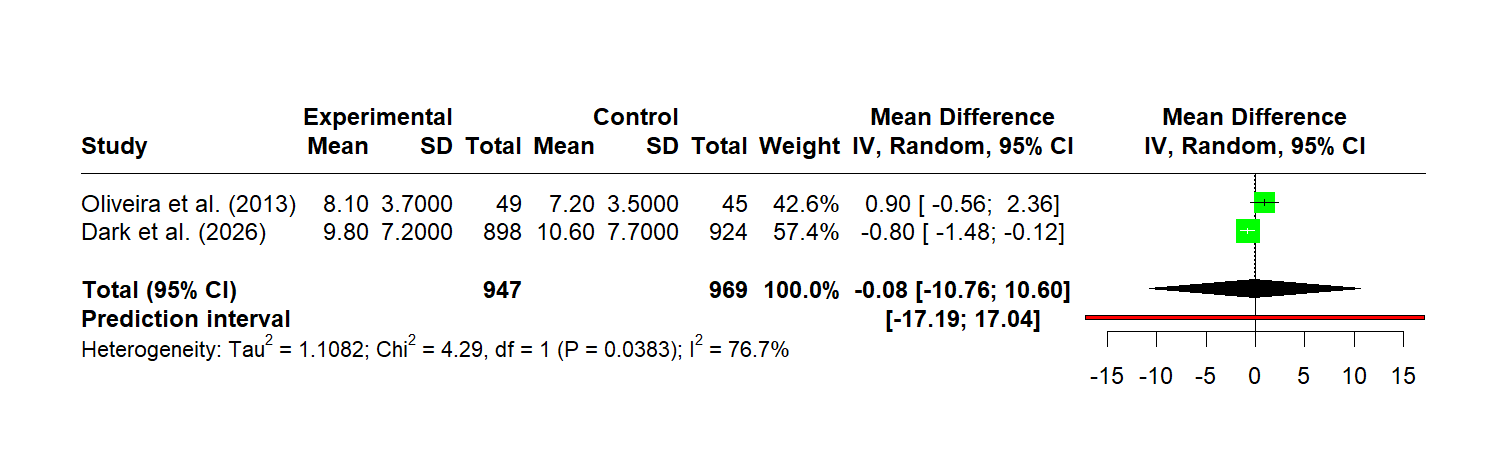


**Figure S21**: Antibiotic duration in participants receiving procalcitonin compared to C-reactive protein


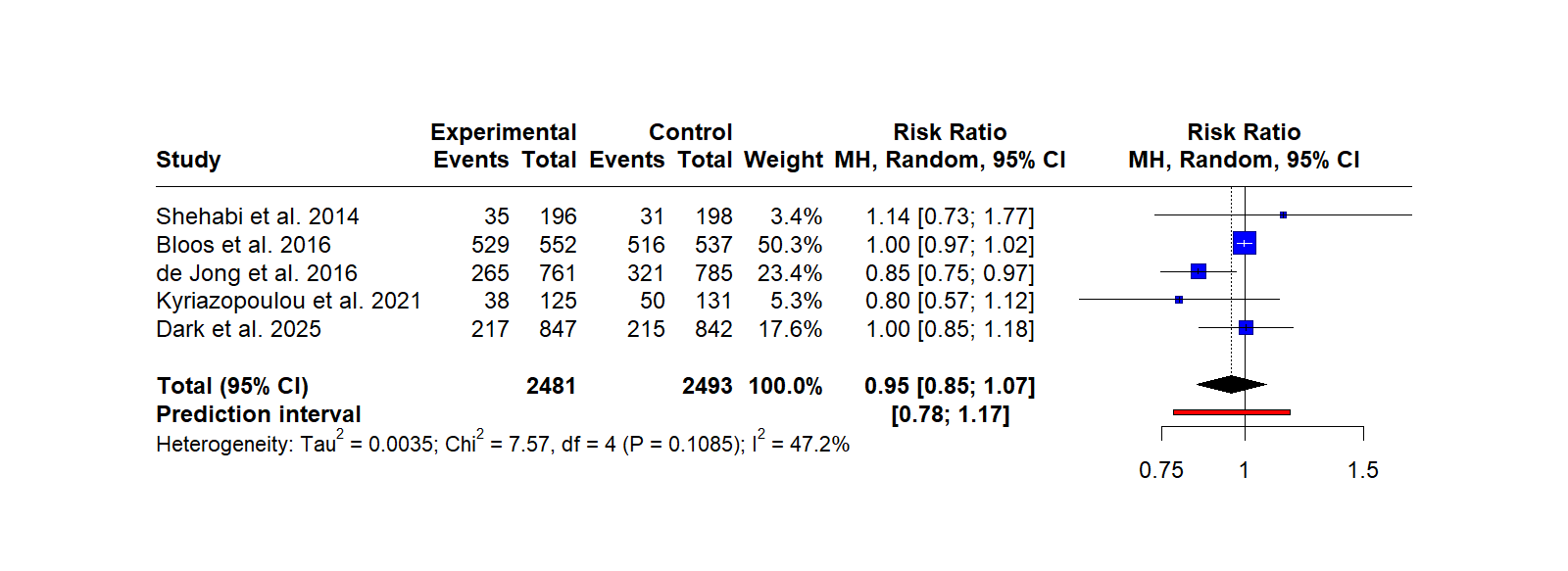


**Figure S22**: Mortality (long-term) in participants receiving procalcitonin compared to standard care

**Publication bias – Funnel plots**


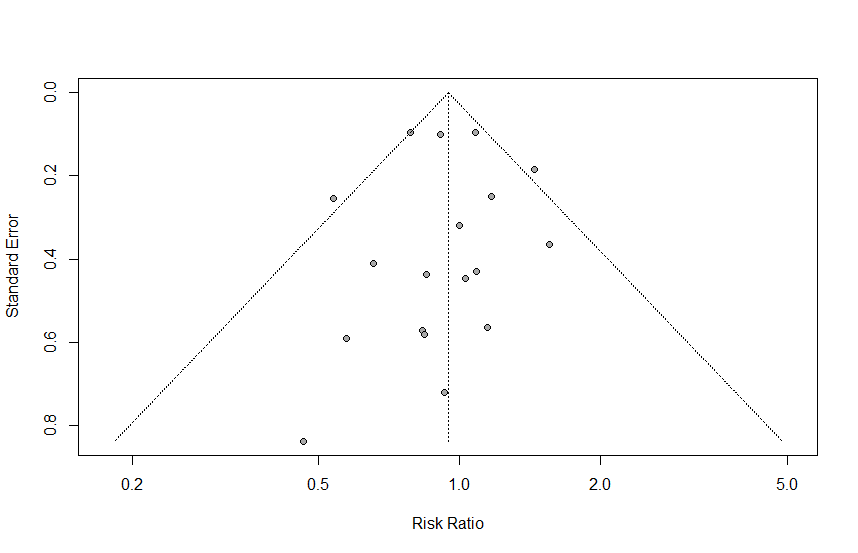


**Figure S23**: Studies comparing procalcitonin to standard care for the outcome of mortality (short-term)


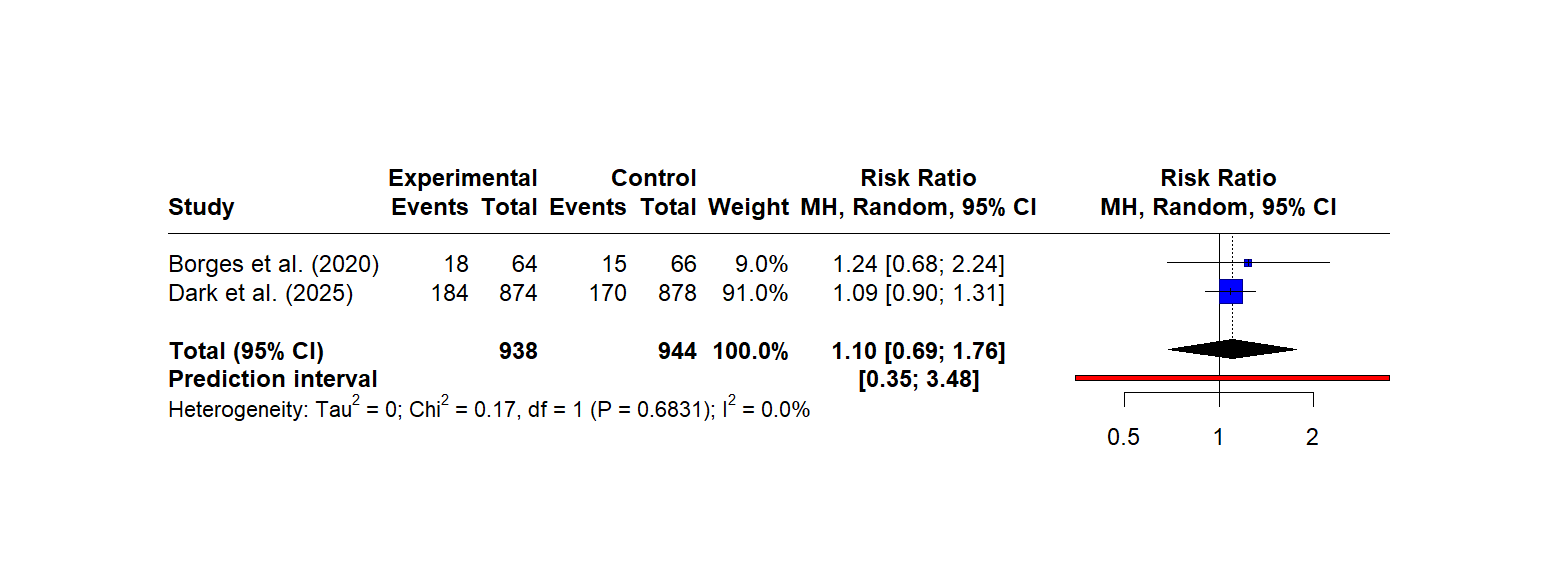


**Figure S24**: Mortality (short-term) in participants receiving C-reactive protein compared to standard care


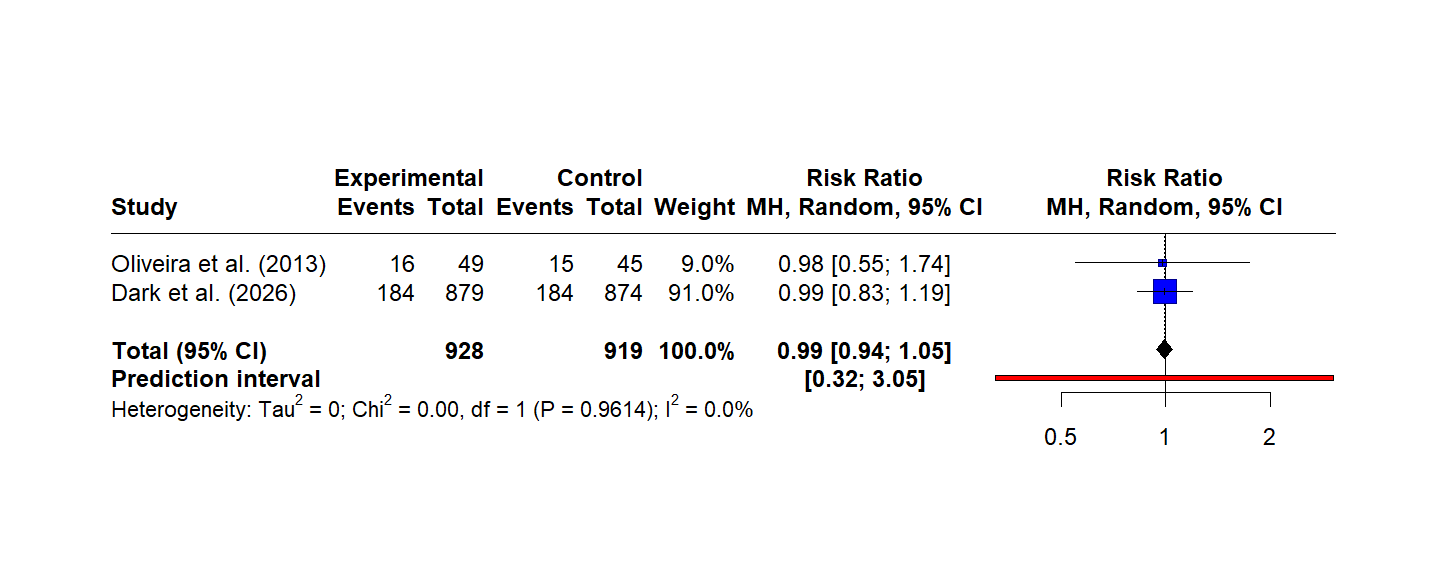


**Figure S25**: Mortality (short-term) in participants receiving procalcitonin compared to C-reactive protein


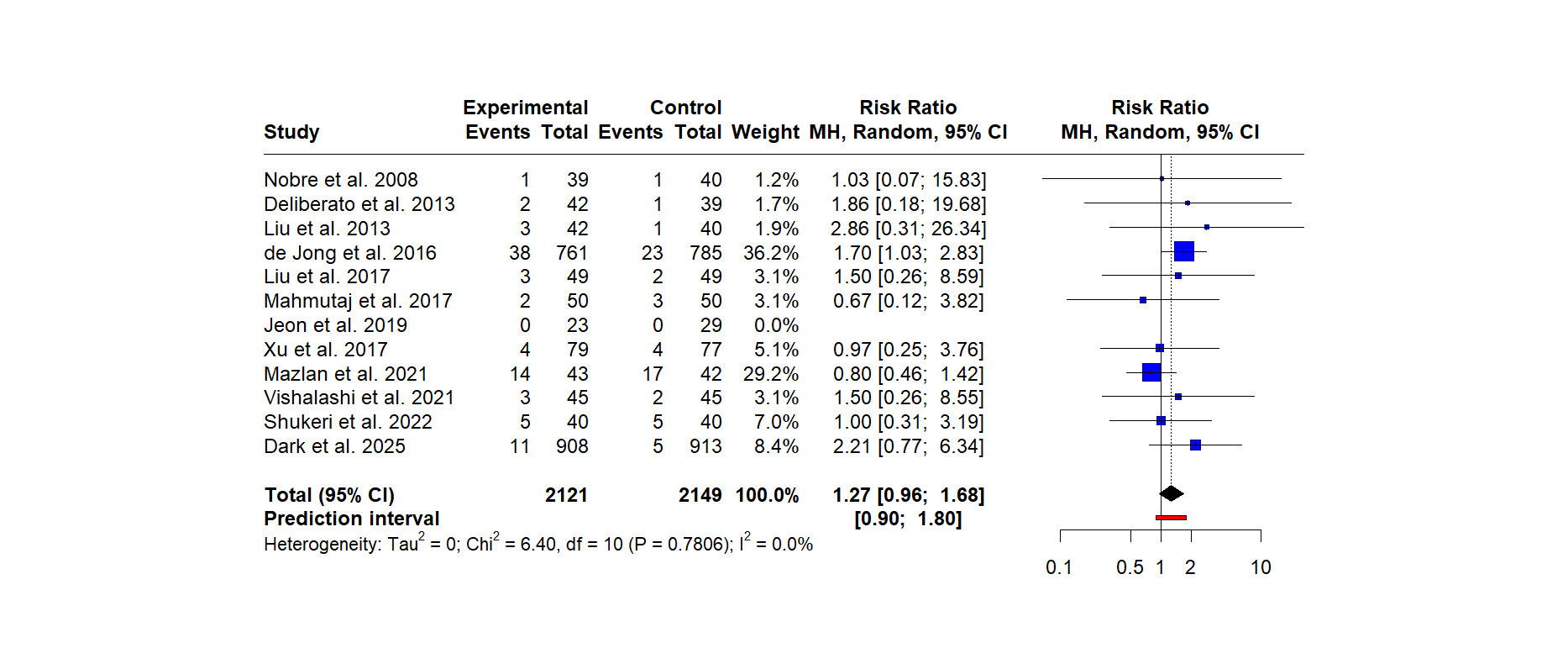


**Figure S26**: Infection recurrence in participants receiving procalcitonin compared to standard care


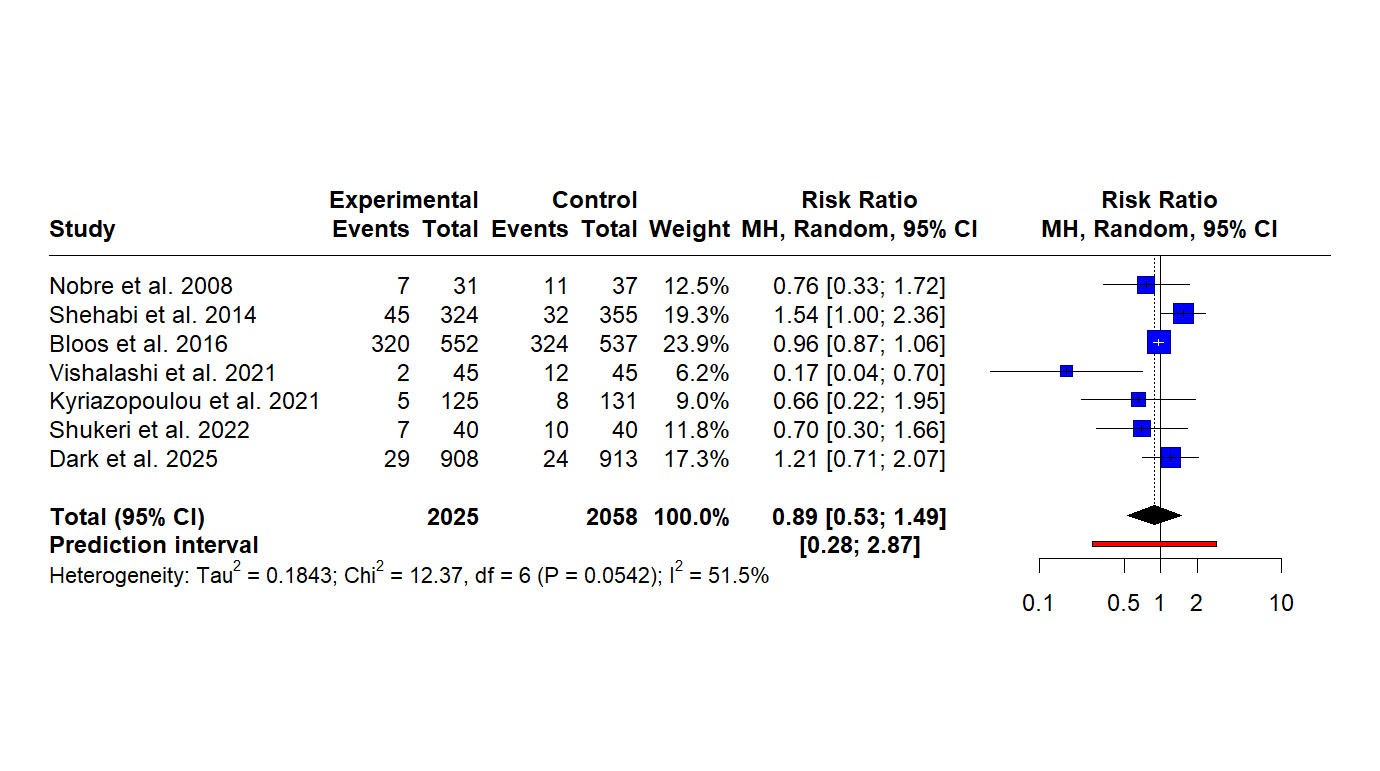


**Figure S27**: Secondary infections or superinfections in participants receiving procalcitonin compared to standard care


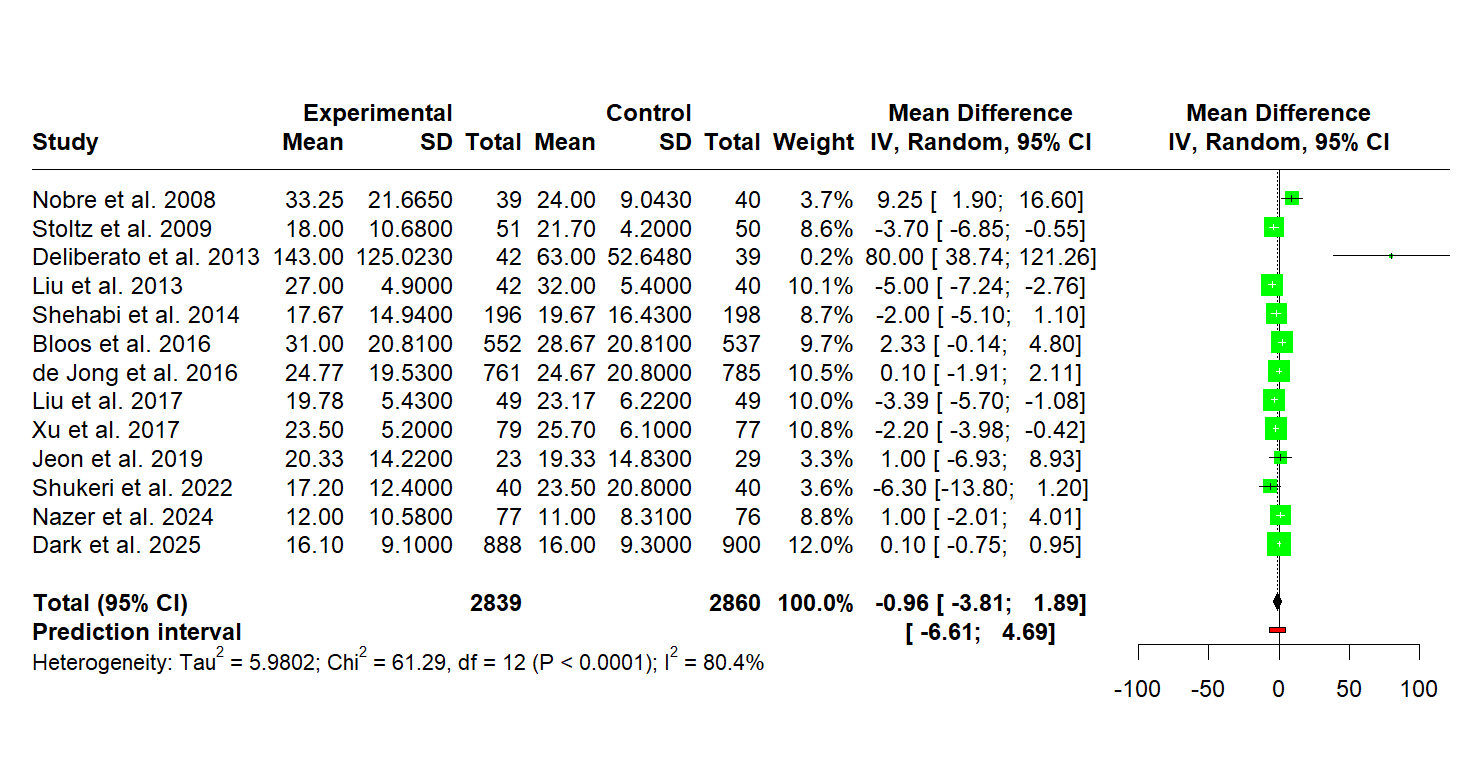


**Figure S28**: Duration of hospital stay in participants receiving procalcitonin compared to standard care


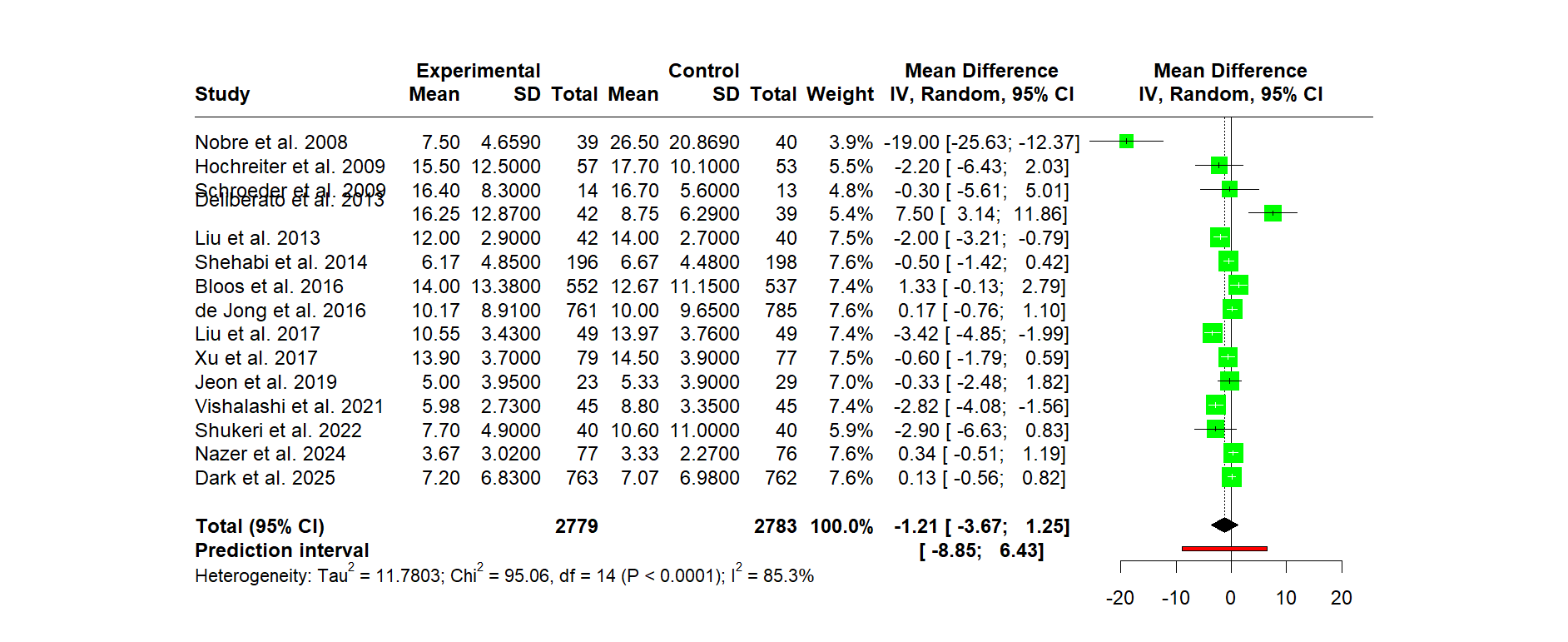


**Figure S29**: Duration of intensive care stay in participants receiving procalcitonin compared to standard care


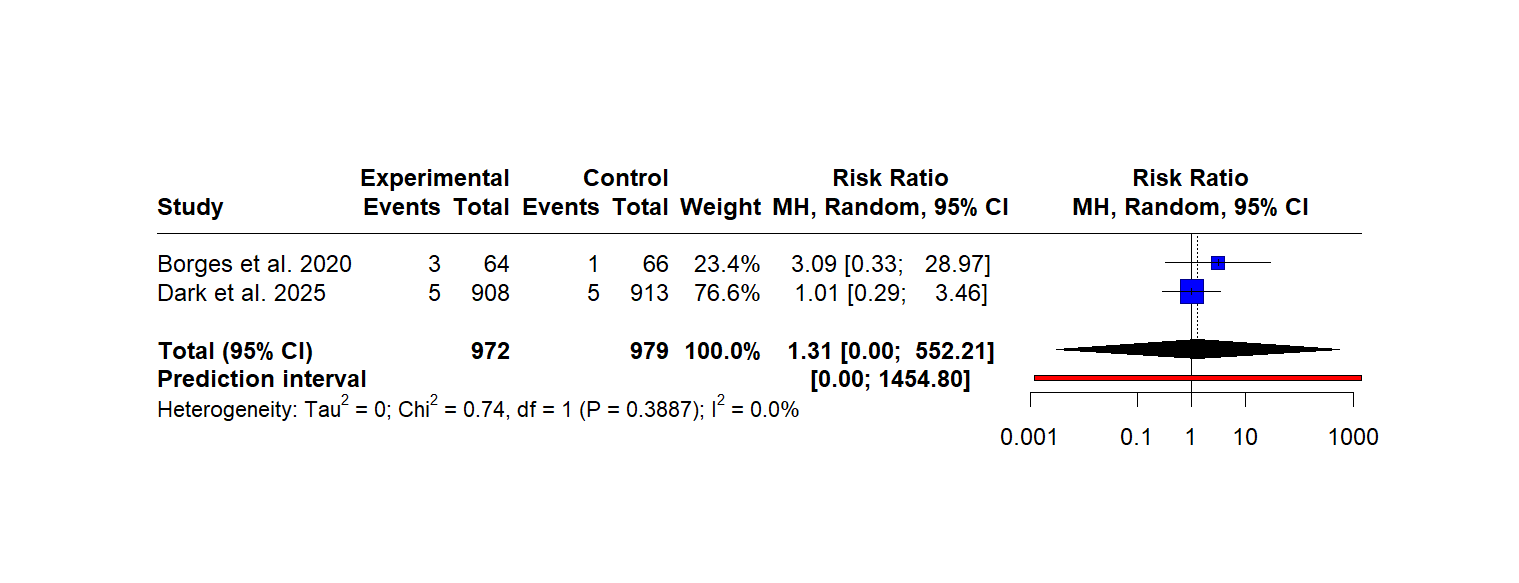


**Figure S30**: Infection recurrence in participants receiving C-reactive protein compared to standard care


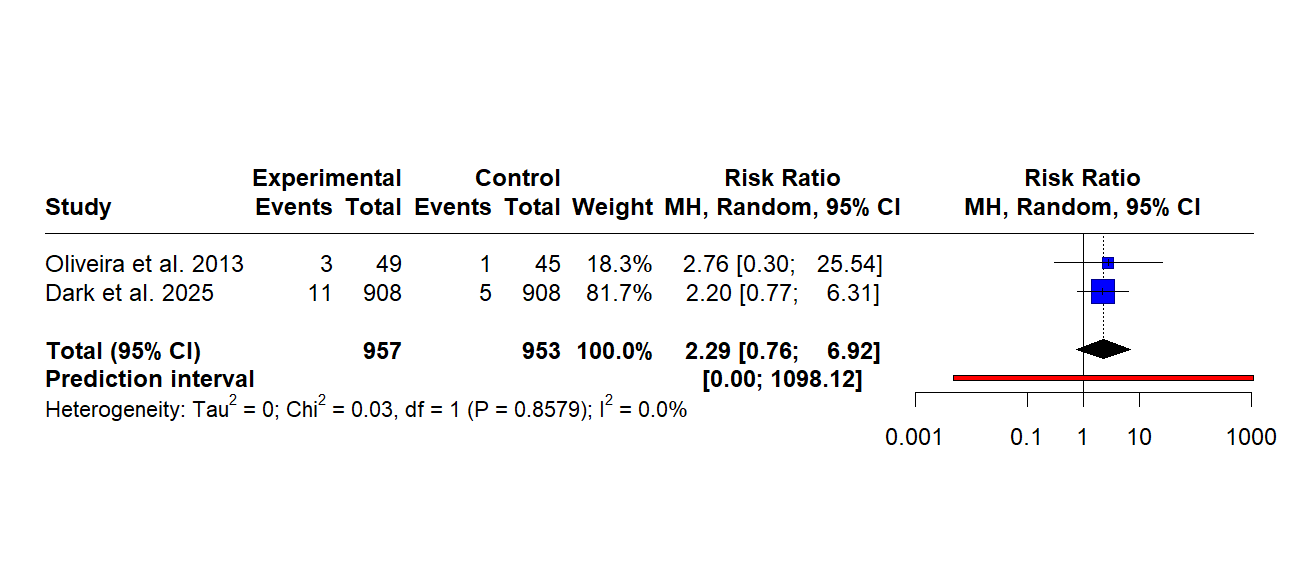


**Figure S31**: Infection recurrence in participants receiving procalcitonin compared to C-reactive protein


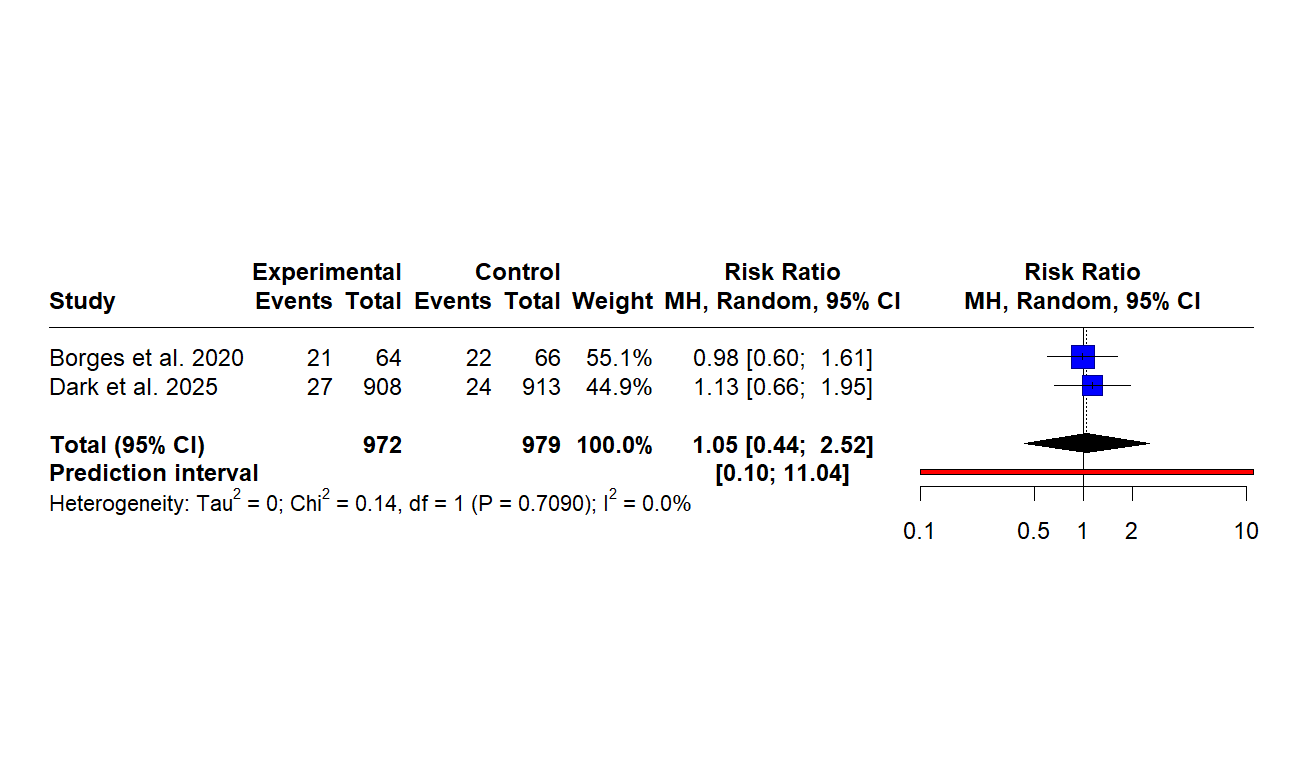


**Figure S32**: Secondary infections or superinfections in participants receiving C-reactive protein compared to standard care

For the comparison of procalcitonin versus C-reactive protein, only one study investigated secondary infection or superinfection. Therefore, no forest plot was generated.


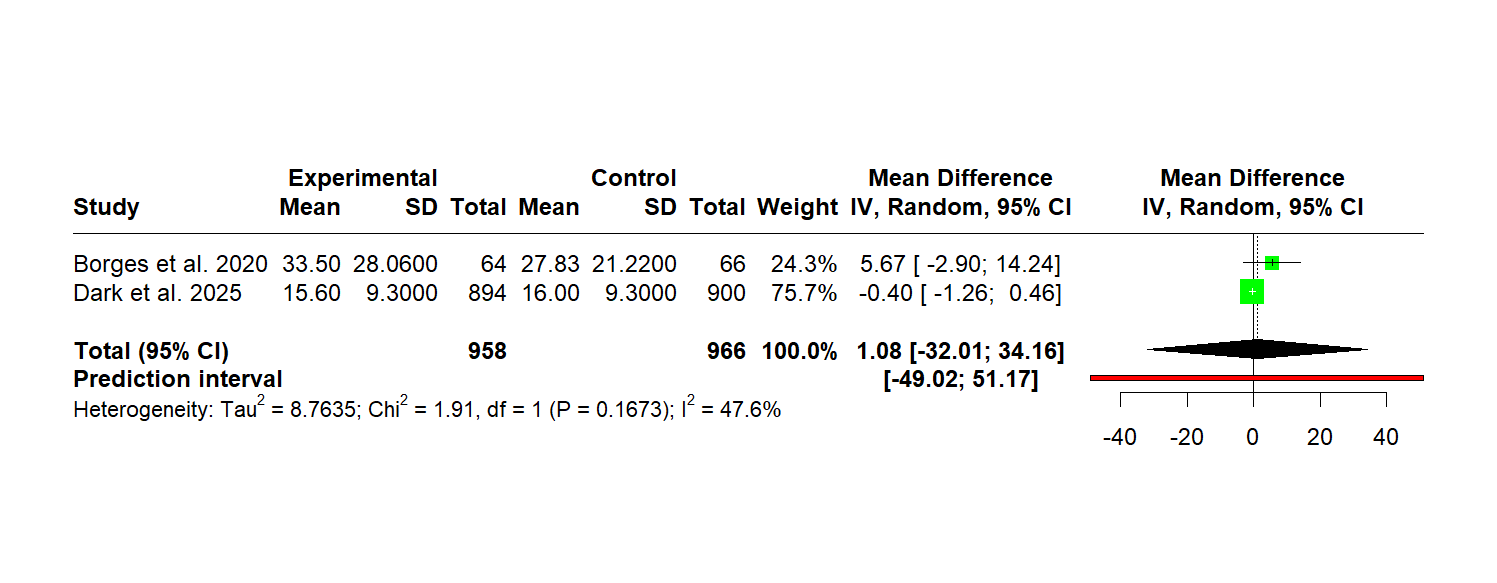


**Figure S33**: Duration of hospital stay in participants receiving C-reactive protein compared to standard care


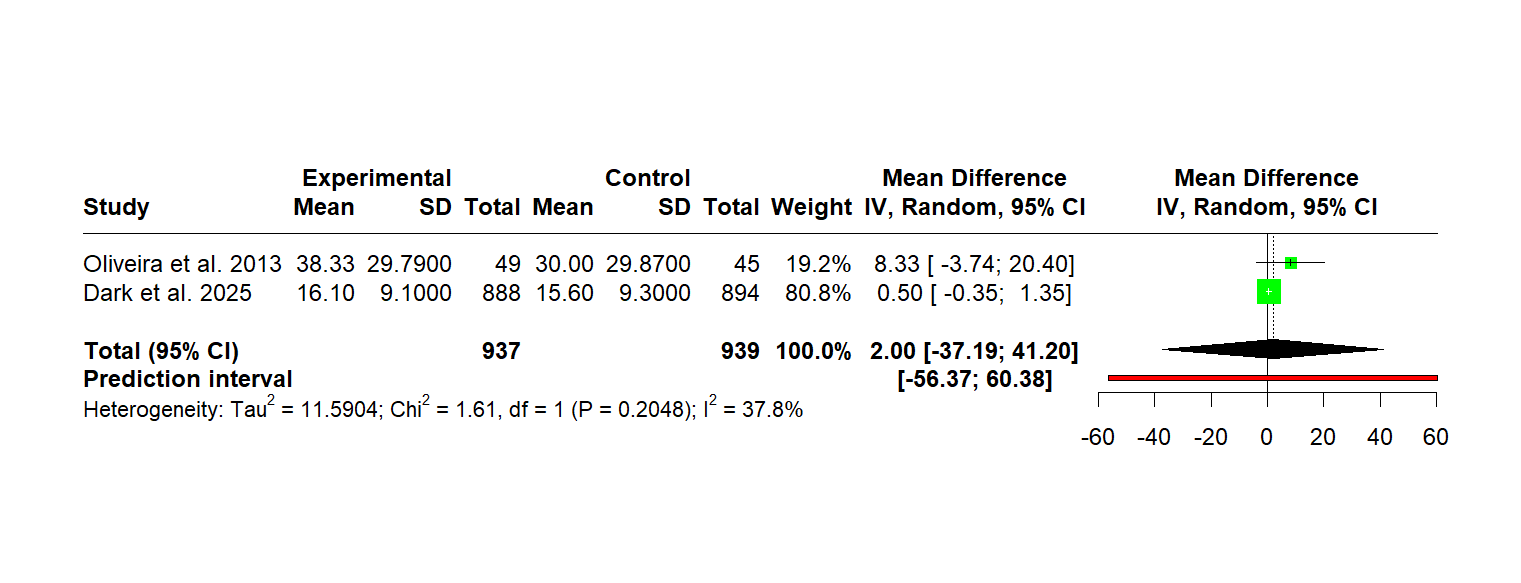


**Figure S34**: Duration of hospital stay in participants receiving procalcitonin compared to C-reactive protein


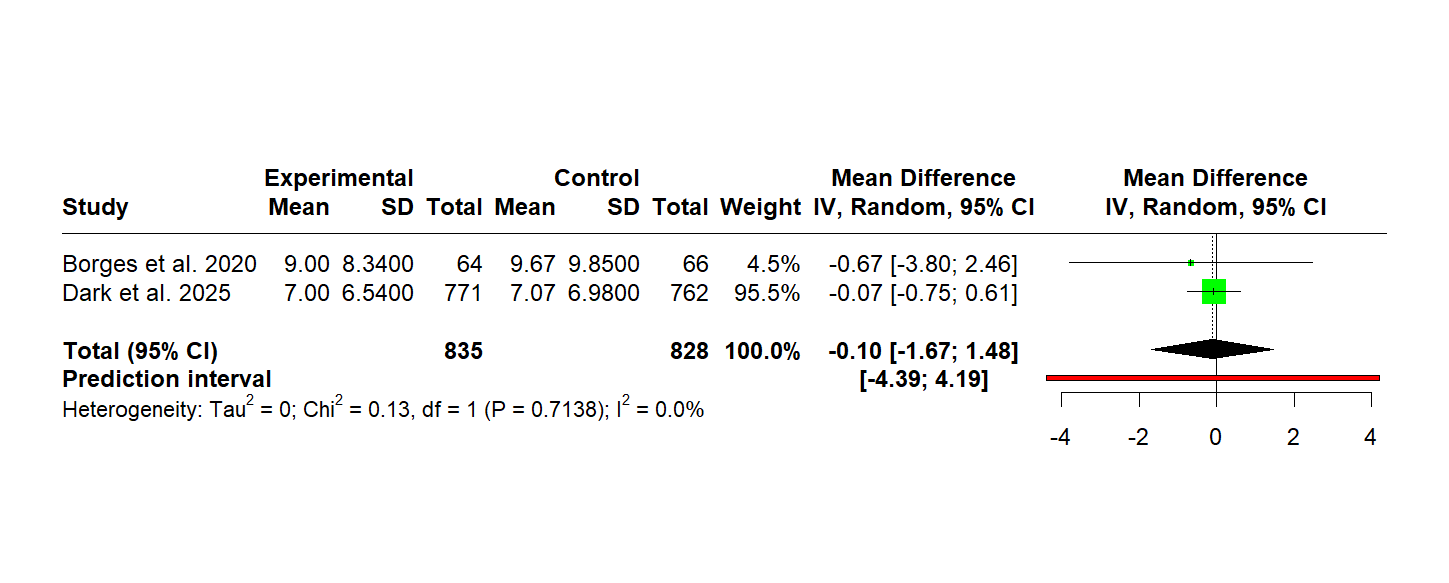


**Figure S35**: Duration of intensive care stay in participants receiving C-reactive protein compared to standard care


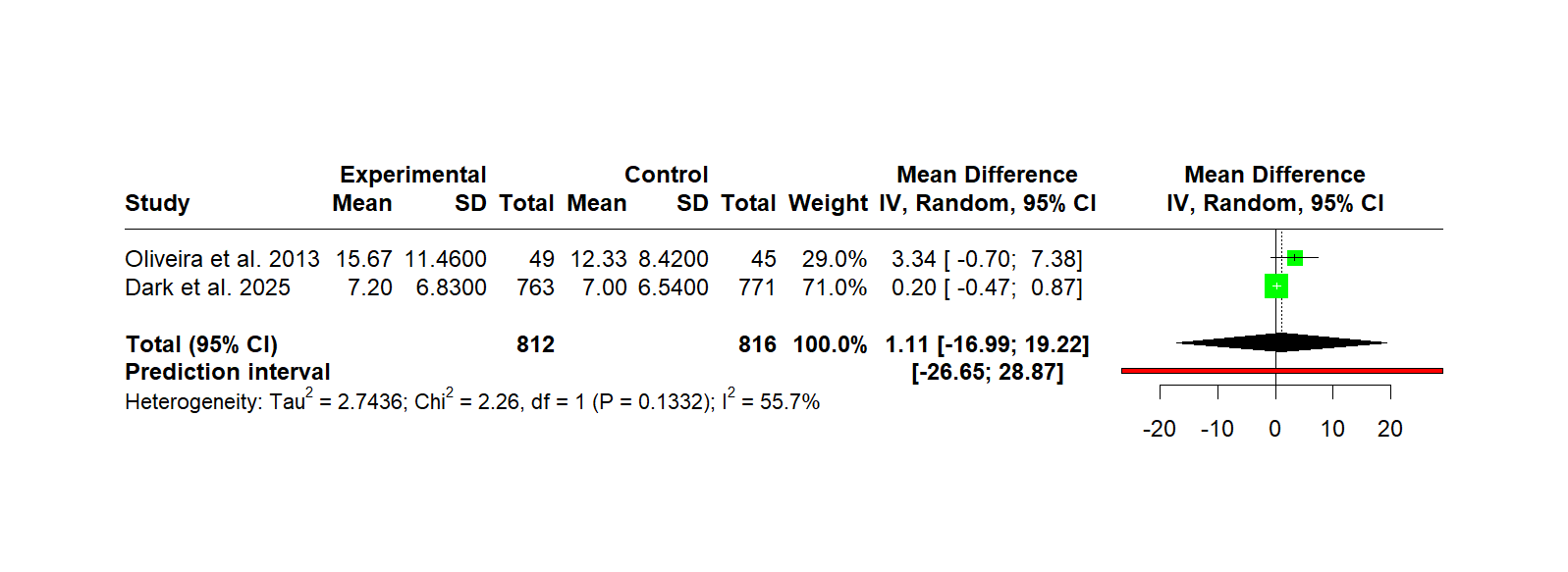


**Figure S36**: Duration of intensive care stay in participants receiving procalcitonin compared to C-reactive protein
