# Supplementary material for: Clinical effectiveness of procalcitonin‐ or C‐reactive protein‐guided antibiotic discontinuation protocols for adult patients who are critically ill with sepsis: a rapid systematic review and meta‐analysis
Source: Anaesthesia. 2026 Jan 8;81(4):556–69. doi: 10.1111/anae.70109 (PMC12973355; doi:10.1111/anae.70109)
Supplement: Supplementary file 3 — Appendix S1. Extended methods. [file ANAE-81-556-s002.docx]

**Appendix S1:** Extended methods

**Data extraction**

Following the Cochrane rapid review guidelines [1], a single reviewer (SR, CS, or SG) conducted data extraction using a predefined, piloted data extraction form. A second reviewer subsequently verified the accuracy and completeness of the extracted data. Our data extraction form included the following items: study characteristics (study authors, year of publication, study design, intensive care setting, and sample size); patient characteristics (age, sex, and sepsis-related information); intervention details (biomarker used, thresholds used for decision making, use of co-interventions including wider biomarker-based antibiotics discontinuation protocols, and adherence to the intervention); comparator details; and primary and secondary outcome details and data where reported. When necessary, we contacted study authors to obtain missing data that was not reported in the published articles.

**Risk of bias assessment**

We used the Cochrane Collaboration’s Risk of Bias version 2 (RoB 2) to assess the risk of bias in included studies [2]. We followed a streamlined approach consistent with rapid review guidelines [1]. For example, for studies with existing RoB 2 assessments from reviews like Kubo et al. [3], one author (SR) verified those assessments and conducted a reassessment if the reviewer disagreed with the existing assessment results, with a second reviewer verifying the reassessment results (CS). For studies without prior RoB2 assessments, two reviewers (SR and CS) independently assessed the risk of bias for each study, with a third reviewer (JD) available to resolve any disagreements. The risk of bias was assessed across the following domains: randomisation, deviations from the intended intervention, missing outcome data, outcome measurement, and selection of reported results, for each of the outcomes. Each study was categorised as having a low risk of bias, some concerns, or a high risk of bias for both primary and secondary outcomes, and traffic light plots of domain-level judgements were visualised using the *robvis* software [4].

We acknowledge the challenges clinical staff face in remaining masked to treatment allocation in RCTs focused on decision-making strategies, which risks performance bias from unintended interventions or differential co-interventions affecting outcome measures [5]. Thus, when assessing this risk of bias domain, we examined the masking strategies used in included studies to mitigate performance bias when making our judgements.

**The certainty of evidence assessment**

A single reviewer (SR) conducted the Grading of Recommendations, Assessment, Development, and Evaluation (GRADE) assessments to evaluate the certainty of evidence for each outcome, rating it as high, moderate, low, or very low certainty [34]. A second reviewer (CS) verified all assessments, with involvement of a third reviewer (JD) as required. The results of the GRADE assessment for each outcome are presented in the ‘Summary of Findings’ tables generated by *GRADEpro* (Evidence Prime, Canada and Poland).

GRADE classes the evidence from RCTs as being of high certainty for effectiveness questions. According to the GRADE rating criteria, we then assessed whether certainty needed to be downgraded for the following factors: imprecision, inconsistency, publication bias, indirectness, and risk of bias [6]. We did not downgrade the certainty level when the risk of bias of the included studies was rated as high, only due to the lack of masking among clinical staff and personnel. We based this decision on the understanding that masking often proves challenging in this type of study. Following Guyatt and colleagues’ recommendation, imprecision was assessed using the optimal information size (OIS) and 95% confidence intervals (CIs), or other indicators such as sample size and the number of events, when the OIS was not applicable [7]. Where appropriate, GRADE’s default thresholds for minimal important differences (RR = 1.25 or 0.75) were applied to determine if the confidence intervals suggested potential clinically important harm or benefit in binary outcomes [7]. Also, to assess whether the confidence intervals were wide enough to warrant a downgrade in certainty, we used the null effect thresholds (i.e., whether the confidence intervals include the null effect, or a risk difference of 0%), rather than minimal important difference thresholds [8]. This was a post hoc decision.

**References**

1. Garritty C, Hamel C, Trivella M, et al. Updated recommendations for the cochrane rapid review methods guidance for rapid reviews of effectiveness. *BMJ* 2024; **384:** e076335. https://doi.org/10.1136/bmj-2023-076335.

2. Sterne JAC, Savović J, Page MJ, et al. Rob 2: A revised tool for assessing risk of bias in randomised trials. *BMJ* 2019; **366:** l4898. https://doi.org/10.1136/bmj.l4898.

3. Kubo K, Sakuraya M, Sugimoto H, et al. Benefits and harms of procalcitonin- or c-reactive protein-guided antimicrobial discontinuation in critically ill adults with sepsis: A systematic review and network meta-analysis. *Crit Care Med* 2024; **52:** e522-e34. doi:<https://dx.doi.org/10.1097/CCM.0000000000006366>

4. McGuinness LA, Higgins JPT. Risk-of-bias visualization (robvis): An r package and shiny web app for visualizing risk-of-bias assessments. *Res Synth Methods* 2020; **12**: 55-61. https://doi.org/10.1002/jrsm.1411.

5. Monaghan TF, Agudelo CW, Rahman SN, et al. Blinding in clinical trials: Seeing the big picture. *Medicina (Kaunas)* 2021; **57**. https://doi.org/10.3390/medicina57070647.

6. Schünemann HJ, Higgins JPT, Vist GE, et al. Completing ‘summary of findings’ tables and grading the certainty of the evidence In: Higgins JPT, Thomas J, Chandler J, Cumpston M, Li T, Page MJ, Welch VA. *Cochrane Handbook for Systematic Reviews of Interventions. Cochrane*, 2019: 375-402.

7. Guyatt GH, Oxman AD, Kunz R, et al. Grade guidelines 6. Rating the quality of evidence–imprecision. *J Clin Epidemiol* 2011; **64:** 1283-93. https://doi.org/10.1016/j.jclinepi.2011.01.012.

8. Guyatt G, Zeng L, Brignardello-Petersen R, et al. Core grade 2: Choosing the target of certainty rating and assessing imprecision. *BMJ* 2025; **389:** e081904. https://doi.org/10.1136/bmj-2024-081904.
